# *Pseudo*-Tris(heteroleptic) Red Phosphorescent Iridium(III) Complexes Bearing a Dianionic *C*,*N*,*C*′,*N*′-Tetradentate Ligand

**DOI:** 10.1021/acs.inorgchem.1c01303

**Published:** 2021-07-22

**Authors:** Vadim Adamovich, Llorenç Benavent, Pierre-Luc T. Boudreault, Miguel A. Esteruelas, Ana M. López, Enrique Oñate, Jui-Yi Tsai

**Affiliations:** † Departamento de Química Inorgánica, Instituto de Síntesis Química y Catálisis Homogénea (ISQCH), Centro de Innovación en Química Avanzada (ORFEO-CINQA), Universidad de Zaragoza-CSIC, 50009 Zaragoza, Spain; ‡ Universal Display Corporation, Ewing, New Jersey 08618, United States

## Abstract

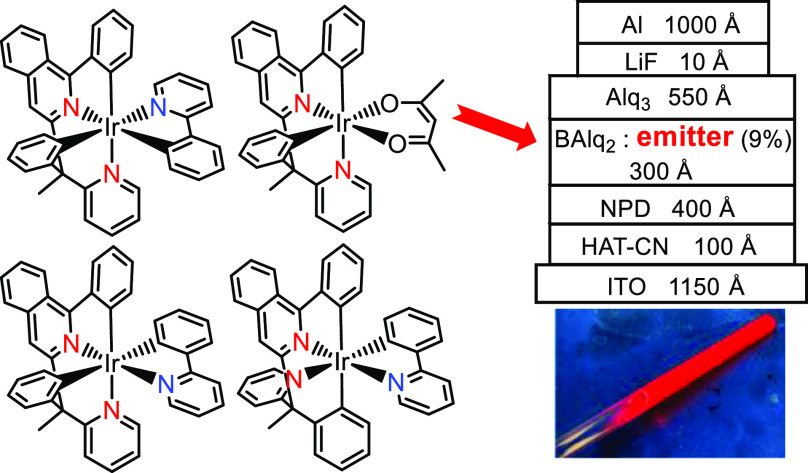

1-Phenyl-3-(1-phenyl-1-(pyridin-2-yl)ethyl)isoquinoline (H_2_MeL) has been prepared by Pd(*N*-XantPhos)-catalyzed “*deprotonative cross-coupling processes*” to synthesize new phosphorescent red iridium(III) emitters (601–732 nm), including the carbonyl derivative Ir(κ^4^-*cis*-*C*,*C*′-*cis*-*N*,*N*′-MeL)Cl(CO) and the acetylacetonate compound Ir(κ^4^-*cis*-*C*,*C*′-*cis*-*N*,*N*′-MeL)(acac). The tetradentate 6e-donor ligand (6tt′) of these complexes is formed by two different bidentate units, namely, an orthometalated 2-phenylisoquinoline and an orthometalated 2-benzylpyridine. The link between the bidentate units reduces the number of possible stereoisomers of the structures [6tt′ + 3b] (3b = bidentate 3e-donor ligand), with respect to a [3b + 3b′ + 3b″] emitter containing three free bidentate units, and it permits a noticeable stereocontrol. Thus, the isomers *fac*-Ir(κ^4^-*cis*-*C*,*C*′-*cis*-*N*,*N*′-MeL){κ^2^-*C*,*N*-(C_6_H_4_-py)}, *mer*-Ir(κ^4^-*cis*-*C*,*C*′-*cis*-*N*,*N*′-MeL){κ^2^-*C*,*N*-(C_6_H_3_R-py)}, and *mer*-Ir(κ^4^-*trans*-*C*,*C*′-*cis*-*N*,*N*′-MeL){κ^2^-*C*,*N*-(C_6_HR-py)} (R = H, Me) have also been selectively obtained. The new emitters display short lifetimes (0.7–4.6 μs) and quantum yields in a doped poly(methyl methacrylate) film at 5 wt % and 2-methyltetrahydrofuran at room temperature between 0.08 and 0.58. The acetylacetonate complex Ir(κ^4^-*cis*-*C*,*C*′-*cis*-*N*,*N*′-MeL)(acac) has been used as a dopant for a red PhOLED device with an electroluminescence λ_max_ of 672 nm and an external quantum efficiency of 3.4% at 10 mA/cm^2^.

## Abstract 2

The proligand 1-phenyl-3-(1-phenyl-1-(pyridine-2-yl)ethyl)isoquinoline is used to generate a new family of neutral phosphorescent red iridium(III) emitters containing a tetradentate ligand, formed by two different bidentate units, and a third bidentate ligand with a good stereocontrol of the resulting [6tt′ + 3b] products. One of the new emitters has been used in the fabrication of an OLED device.

## Introduction

Phosphorescent iridium(III) emitters currently receive a great deal of attention due to their ability to reach internal quantum efficiencies close to unity in their organic light-emitting diode (OLED) devices.^1^ Because their emissions are ligand-dependent, there is growing interest in heteroleptic complexes, particularly in those bearing three different ligands. The reason for this is that the emissive properties could be fine-tuned by an appropriate building of the metal coordination sphere by means of an adequate selection of the ligands; that is, it should be possible to design emitters according to the requirements of a given application.^1,2^

The building of iridium(III) complexes of type [3b + 3b′ + 3b″] with three different 3e-donor bidentate ligands is challenging. The preparation methods involving one-pot procedures give statistical mixtures of ligand distribution products, where the maximum yield of each one can become about 30%, before the necessary column chromatography separation.^3^ The synthesis through the sequential coordination of the different ligands is a tedious multistep procedure,^4^ which has some success if the three ligands are quite different. An additional problem is the existence of structural isomers, which display their own photophysical properties.^5^ An interesting approach to solve this dare is to bind two ligands, 3b and 3b′, to form a heteroleptic 6e-donor tetradentate ligand, 6tt′, with two different bidentate moieties. In this way, the ligand distribution possibilities in the resulting *pseudo*-tris(heteroleptic) [6tt′ + 3b″] compounds are reduced, which allows an increase of the reaction yield and facilitates the chromatographic separation. In addition, a better structural control should be reached as a result of the increase of the rigidity of the system, which provides a decrease in the number of feasible stereoisomers.

**Chart 1:**
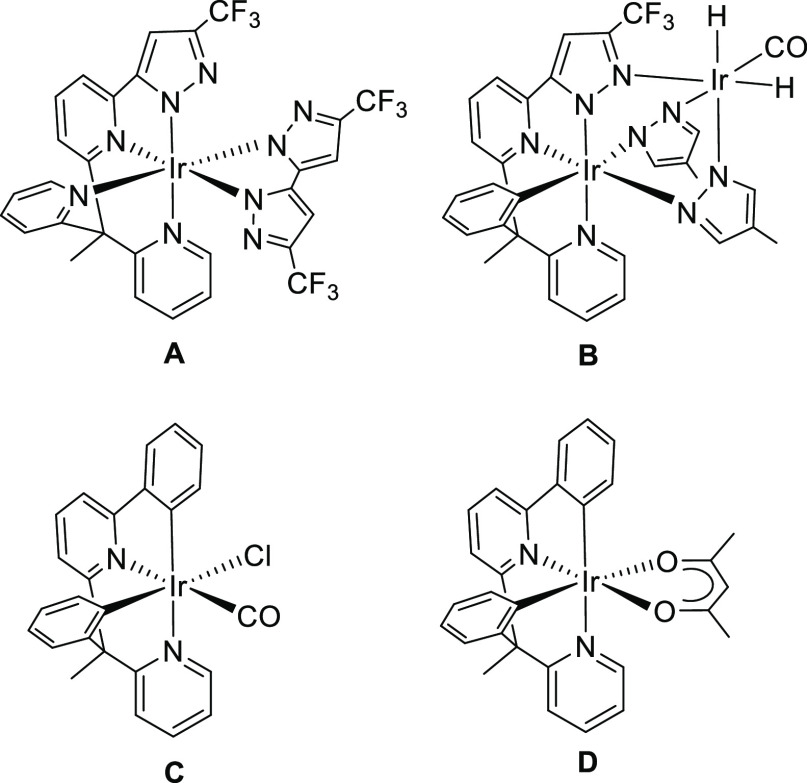
Iridium(III) Emitters Bearing Nonplanar Tetradentate Ligands Formed by Two Different Bidentate Moieties

Tetradentate ligands are less common than monodentate, bidentate, and tridentate. Macrocyclic and rigid acyclic dispositions providing a planar skeleton are the most frequently used.^6^ Iridium(III) emitters bearing nonplanar tetradentate ligands are very scarce,^7^ and particularly rare are those formed by two different bidentate moieties. As far as we know, only three ligands of this class have been previously used to prepare iridium(III) emitters (Chart 1). The 2,2′-(1-(6-(3-trifluoromethyl-1*H*-pyrazol-5-yl)pyridin-2-yl)ethane-1,1-diyl)dipyridine molecules afford monoanionic *N*,*N*′,*N*″,*N*″-tetradentate ligands (7tt′), which stabilize sky-blue [7tt′ + 2b] emitters **A**,^8^ whereas exchanging one of the peripheral pyridine for a phenyl group leads to 2-(3-trifluoromethyl-1*H*-pyrazol-5-yl)-6-(1-phenyl-1-(pyridyn-2-yl)ethyl)pyridine, which forms a dianionic *N*,*N*′,*C*,*N*″-tetradentate ligand 6tt′. This anion uses the free nitrogen atom of the pyrazolate group to generate the green binuclear emitters **B**.^9^ We have recently shown that the *ortho*-CH bond activation of both phenyl groups of 2-phenyl-6-(1-phenyl-1-(pyridin-2-yl)ethyl)pyridine gives a dianionic *C*,*N*,*C*′,*N*′-tetradentate 6tt′ ligand, which allows the access to blue-green and green iridium(III) emitters, **C** and **D**, of classes [6tt′ + 1m + 2m] and [6tt′ + 3b], respectively.^10^

Complex **D** can be viewed as a *pseudo*-tris(heteroleptic) iridium(III) emitter with the metal coordination sphere formed by three different bidentate moieties, an orthometalated 2-phenylpyridine, an orthometalated 2-benzylpyridine type ligand, and an acetylacetonate group (acac). Its lowest-unoccupied molecular orbital (LUMO) is mainly centered on the orthometalated 2-phenylpyridine moiety, specifically on the pyridyl group, whereas the highest-occupied molecular orbital (HOMO) – 1 and HOMO lie at the metal center, both metalated phenyl groups, and to a lesser extent at the acac group. The green emission was attributed to a T_1_ excited state, which is originated mainly by mixed HOMO – 1-to-LUMO and HOMO-to-LUMO charge-transfer transitions. Therefore, in order to modify the emission wavelength, two different actions could be performed: to introduce substituents at the phenyl groups or to replace the pyridyl group of the 2-phenylpyridine moiety with another heterocycle. In this context, it should be mentioned that the presence of fluorine substituents at the phenyl group of an orthometalated 2-phenylpyridine usually produces a blue shift with regard to the unsubstituted chromophore,^7c,11^ although their use is limited by issues involving partial defluorination during the OLED assembly.^12^ In contrast, increasing the conjugation of the heterocycle by fused aromatic rings gives rise to a red shift.^13^ According to this, we decided to replace the phenylpyridine unit of the tetradentate ligand of complex **D** with a phenylisoquinoline group in the search for red emitters with the structural rigidity of the latter. Furthermore, we wished to investigate how the rigidity of the tetradentate ligand predetermines the stereochemistry of the [6tt′ + 3b] compound when an orthometalated 2-phenylpyridine is employed as a 3b ligand, what isomers can be obtained, and under what experimental conditions.

**Chart 2:**
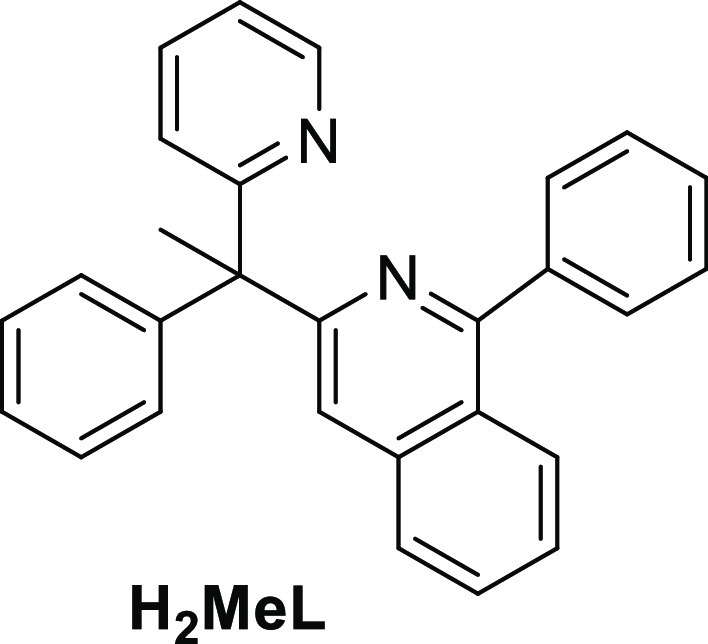
1-Phenyl-3-(1-phenyl-1-(pyridine-2-yl)ethyl)isoquinoline (H_2_MeL)

The present paper shows the preparation of an organic molecule, 1-phenyl-3-(1-phenyl-1-(pyridine-2-yl)ethyl)isoquinoline (Chart 2), which allows to generate a new dianionic *C*,*N*,*C*′,*N*′-tetradentate 6tt′ ligand, formed by two different bidentate moieties. It also describes its coordination to iridium and the stereocontrol in the formation of the [6tt′ + 3b] isomers when an orthometalated 2-phenylpyridine type ligand is used as the 3b unit. Furthermore, the photophysical properties of the new compounds, including the fabrication of a PhOLED device based on one of them, are reported.

## Results and Discussion

### Preparation of 1-Phenyl-3-(1-phenyl-1-(pyridine-2-yl)ethyl)isoquinoline (H_2_MeL)

**Scheme 1:**
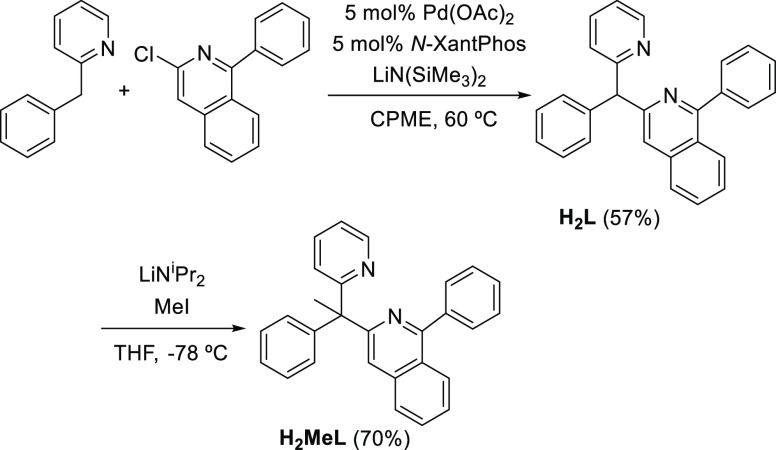
Synthesis of 1-Phenyl-3-(1-phenyl-1-(pyridine-2-yl)ethyl)isoquinoline (H_2_MeL)

This molecule was prepared according to Scheme 1. We initially performed Pd(*N*-XantPhos)-catalyzed “*deprotonative cross-coupling processes*”^14^ involving 3-chloro-1-phenylisoquinoline and 2-benzylpyridine in the presence of LiN(SiMe_3_)_2_ using cyclopentyl methyl ether (CPME) as a solvent. The catalysis afforded 1-phenyl-3-(phenyl(pyridin-2-yl)methyl)isoquinoline (H_2_L) as a yellow solid in 57% yield. The procedure had been previously proved to be efficient for a variety of aryl halides and substrates with weakly acidic C(sp^3^)–H bonds including diarylmethanes,^15^ allylbenzenes,^16^ sulfoxides,^17^ sulfones,^18^ amides,^19^ benzylic phosphine oxides,^20^ and η^6^-arene complexes of toluene derivatives and benzylic amines.^21^ Furthermore, it had facilitated rapid access to triarylmethanes with interesting biological activity.^22^ In order to prevent the formation of trityl-type radicals, the C(sp^3^)H-hydrogen atom was subsequently replaced with a methyl group through its abstraction with lithium diisopropylamide in tetrahydrofuran (THF) at −78 °C and posterior treatment of the resulting anion with methyl iodide. After purification of the reaction crude by silica gel column chromatography, the designed organic molecule 1-phenyl-3-(1-phenyl-1-(pyridine-2-yl)ethyl)isoquinoline (H_2_MeL) was obtained as a white solid in 70% yield.

### Coordination to Iridium

**Scheme 2:**
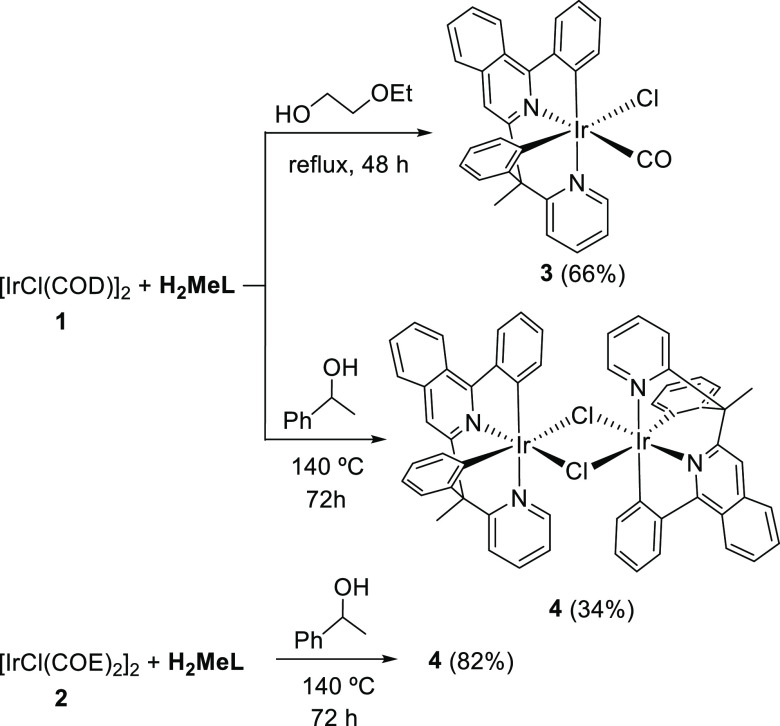
Synthesis of Complexes **3** and **4**

Once the desired organic molecule was generated, we investigated its coordination to iridium with the aim of preparing a dimer [Ir(μ-Cl)(6tt′)]_2_. It should allow us to enter in the chemistry of iridium(III) complexes with the designed ligand. We were inspired by our previous work on the related proligand 2-phenyl-6-(1-phenyl-1-(pyridin-2-yl)ethyl)pyridine.^10^ Thus, in the search for the optimization of the synthesis procedure, we selected the known dimers [Ir(μ-Cl)(η^4^-COD)]_2_ and [Ir(μ-Cl)(η^2^-COE)_2_]_2_ [COD = 1,5-cyclooctadiene (**1**), COE = cyclooctene (**2**)] as organometallic precursors and studied their reactions with H_2_MeL in two different alcohols, the usual one 2-ethoxyethanol and 1-phenylethanol (Scheme 2).

**Figure 1:**
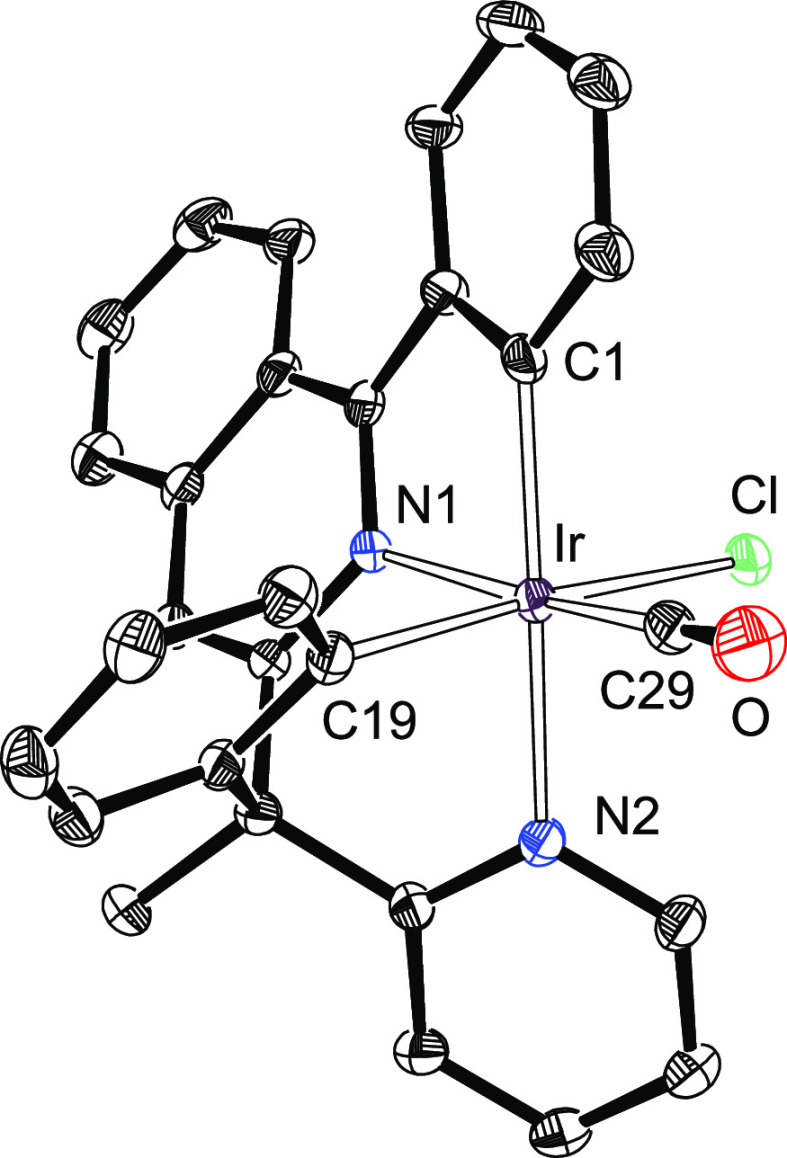
X-ray structure of **3** showing 50% thermal ellipsoid probability (hydrogen atoms have been omitted). Selected bond lengths (Å) and angles (degree): Ir–C(1) = 2.012(4), Ir–C(19) = 2.035(3), Ir–C(29) = 1.849(4), Ir–N(1) = 2.054(3), Ir–N(2) = 2.124(3), Ir–Cl = 2.4651(9); N(1)–Ir–N(2), 91.72(11); C(1)–Ir–N(2) = 168.31(13), C(29)–Ir–N(1) = 169.88(14), Cl–Ir–C(19) = 170.53(10), N(1)–Ir–N(2) = 91.72(11), C(1)–Ir–C(19) = 98.95(14), C(1)–Ir–N(1) = 79.07(13), C(19)–Ir–N(1) = 81.92(13), C(19)–Ir–N(2) = 86.73(13).

Treatment of complex **1** with 1.0 equiv of H_2_MeL in 2-ethoxyethanol under reflux for 48 h leads to the carbonyl derivative Ir(κ^4^-*cis*-*C*,*C*′-*cis*-*N*,*N*′-MeL)Cl(CO) (**3**), which was isolated as an orange solid in 66% and characterized by X-ray diffraction analysis. Figure 1 displays a view of the complex. The structure proves the presence of a carbonyl group coordinated to iridium and the generation of a tetradentate 6tt′ ligand as a result of the N-coordination of both heterocycles of the organic precursor and the activation of an *ortho*-CH bond of both phenyl groups. The polyhedron around the iridium atom is the expected octahedron with the phenyl substituent of the 2-phenylisoquinoline moiety disposed trans to the pyridyl ring of the 2-benzylpyridine moiety [C(1)–Ir–N(2) = 168.31(13)°]. The carbonyl group and the chloride anion lie in the plane perpendicular to the C(1)–Ir–N(2) direction. They are disposed trans to the isoquinolyl unit and the remaining phenyl group, with angles C(29)–Ir–N(1) and Cl–Ir–C(19) of 169.88(14) and 170.53(10)°, respectively. In accordance with the presence of the carbonyl ligand, the infrared (IR) spectrum of the complex contains a ν(CO) band at 2023 cm^–1^, whereas the ^13^C{^1^H} NMR spectrum in dichloromethane-*d*_2_ shows a singlet at 172.6 ppm.

The formation of **3** and its structure agree well with those of the dipyridine counterpart complex **C** (Chart 1), which was prepared under similar conditions by reaction of **1** with 2-phenyl-6-(1-phenyl-1-(pyridin-2-yl)ethyl)pyridine. In both cases, the carbonyl ligand comes from the solvent of the reaction. The facility of iridium and platinum group metals to promote the dehydrogenation of primary alcohols to aldehydes^23^ and the abstraction of the CO group from aldehydes^24^ is well known. In contrast to 2-ethoxyethanol, the secondary alcohol 1-phenylethanol does not undergo decarbonylation. Thus, the reaction of **1** with H_2_MeL in this alcohol under reflux for 3 days affords a brown solid, which corresponds to the desired dimer [Ir(μ-Cl)(κ^4^-*cis*-*C*,*C*′-*cis*-*N*,*N*′-MeL)]_2_ (**4**), according to its MALDI-TOF spectrum ([M/2]^+^ 612.2) and C, H, N-elemental analysis. The yield of the preparation is modest (34%). However, a significant improvement up to 82% is achieved when, under the same conditions, COE-dimer **2** is used as the organometallic precursor instead of complex **1**.

### Reactions and [6tt′ + 3b] Complexes Keeping the Disposition of the Tetradentate Ligand

**Scheme 3:**
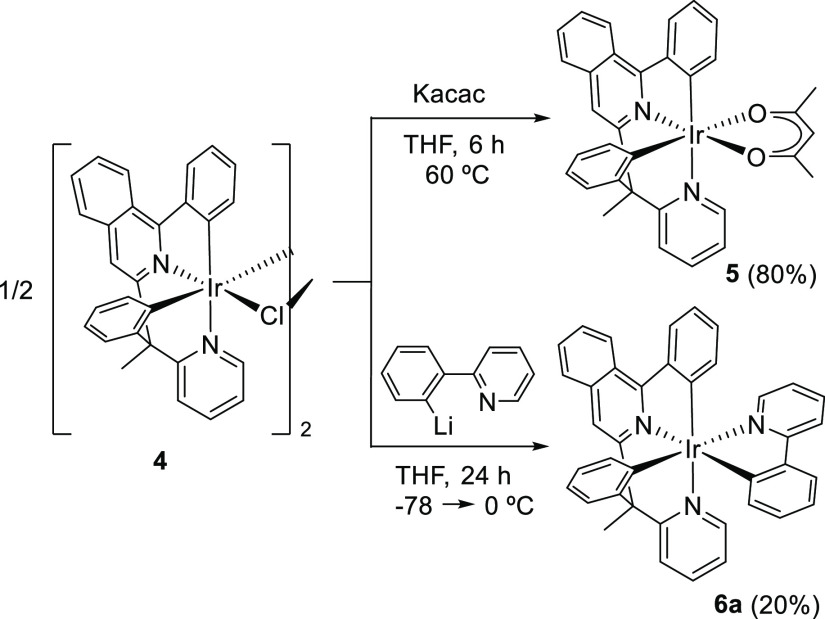
Preparation of Complexes **5** and **6a**

Having obtained the desired starting compound [Ir(μ-Cl)(6tt′)]_2_, we next addressed the task of replacing the chloride anion of the mononuclear unit with a 3b ligand. The aim was to generate new species [6tt′ + 3b], which would really be [3b + 3b′ + 3b″], since the 6tt′ ligand is certainly a [3b + 3b′] moiety. For this purpose, we selected Kacac and Li[py-2-C_6_H_4_] as precursors of the 3b ligand (Scheme 3).

**Figure 2:**
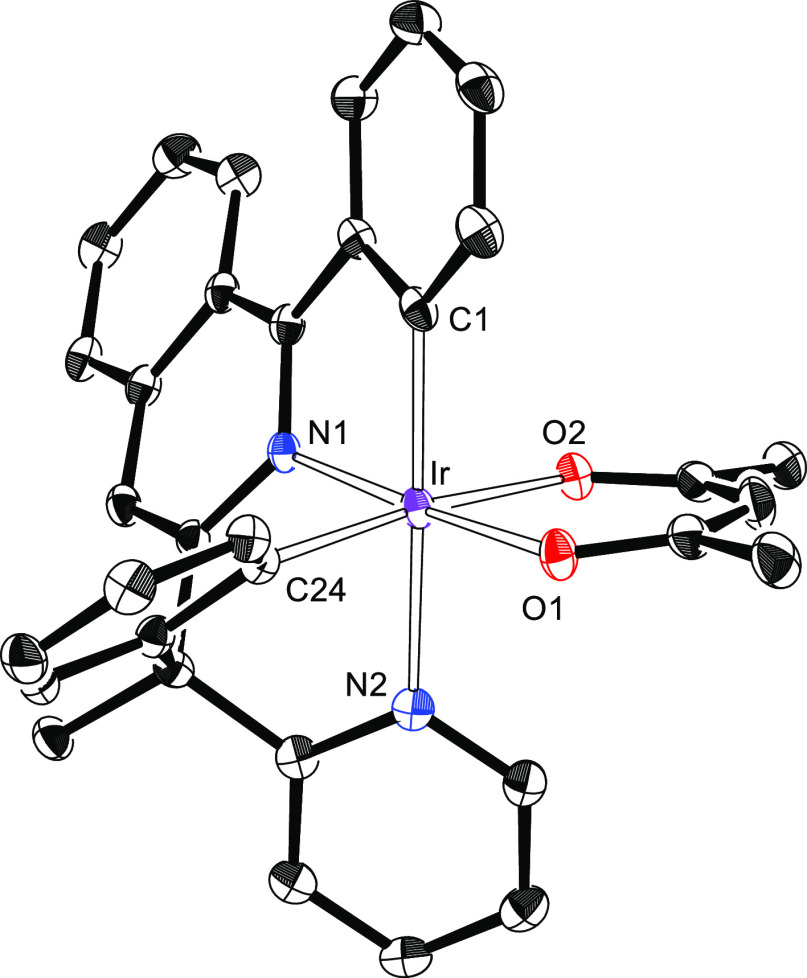
X-ray structure of **5**, showing 50% thermal ellipsoid probability (hydrogen atoms have been omitted). Selected bond lengths (Å) and angles (degree): Ir–C(1) = 1.991(4), Ir–C(24), 1.996(4), Ir–N(1) = 1.970(3), Ir–N(2) = 2.128(3), Ir–O(1) = 2.049(2), Ir–O(2), 2.135(3); C(1)–Ir–N(2) = 170.90(13), O(1)–Ir–N(1) = 174.77(11), O(2)–Ir–C(24) = 172.29(13).

Treatment of dimer **4** with Kacac in THF at 60 °C for 6 h leads to the acetylacetonate derivative Ir(κ^4^-*cis*-*C*,*C*′-*cis*-*N*,*N*′-MeL)(acac) (**5**), which was isolated as a reddish brown solid in 80% yield after silica column chromatography purification and characterized by X-ray diffraction analysis. Its structure (Figure 2) displays the same disposition for the donor atoms of the tetradentate ligand as that observed in **3**. Thus, the polyhedron around the metal center can be rationalized as a distorted octahedron with the phenyl substituent of the 2-phenylisoquinoline moiety disposed trans to the pyridyl ring of the 2-benzylpyridine moiety [C(1)–Ir–N(2) = 170.90(13)°], whereas the acac ligand lies at a perpendicular plane with the oxygen atoms O(1) and O(2) situated trans to the isoquinolyl group [O(1)–Ir–N(1) = 174.77(11)°] and to the phenyl of the benzyl moiety [O(2)–Ir–C(24) = 172.29(13)°], respectively.

**Figure 3:**
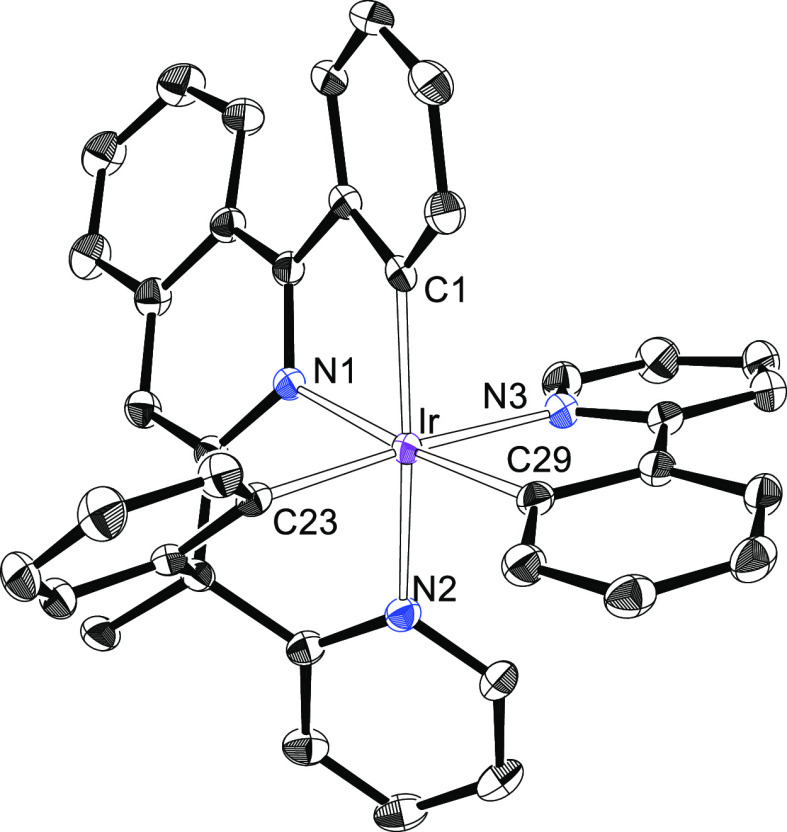
X-ray structure of **6a** showing 50% thermal ellipsoid probability (hydrogen atoms have been omitted). Selected bond lengths (Å) and angles (degree): Ir–C(1) = 2.005(3), Ir–C(23) = 2.023(3), Ir–C(29) = 2.032(3), Ir–N(1) = 2.067(2), Ir–N(2) = 2.110(2), Ir–N(3) = 2.110(2); N(1)–Ir–C(29) = 174.26(10), N(2)–Ir–C(1) = 169.59(10) N(3)–Ir–C(23) = 175.77(10).

The chloride anion of the mononuclear units of **4** can be similarly replaced with an orthometalated 2-phenylpyridine ligand. In contrast to acac, this C,N-bidentate group is asymmetrical. Thus, keeping the disposition of the tetradentate ligand, its coordination can in principle afford two different isomers: one of them with the N-heterocycles in fac disposition (**6a**) and the other bearing the N-heterocycles in the mer position with the isoquinolyl moiety of the tetradentate ligand trans disposed to the pyridyl ring of the bidentate group (**6b**). Treatment of the dimer with Li[py-2-C_6_H_4_] in THF at room temperature for 24 h produces the expected substitution and regioselectively gives only one of the possible isomers of Ir(κ^4^-*cis*-*C*,*C*′-*cis*-*N*,*N*′-MeL){κ^2^-*C*,*N*-(C_6_H_4_-py)} (**6**), according to its ^1^H and ^13^C{^1^H} NMR spectra (Figures S58 and S59). This isomer was isolated as dark-red crystals, suitable for X-ray diffraction analysis, in 20% yield after the purification of the reaction crude by neutral-alumina column chromatography. Figure 3 shows a view of its structure, which reveals a fac disposition for the N-heterocycles and therefore proves the formation **6a**. Thus, the coordination polyhedron around the iridium center can be rationalized as a distorted octahedron with the pyridyl ring of the 3b ligand trans disposed to the phenyl group of the benzyl moiety [N(3)–Ir–C(23) = 175.77(10)°], the pyridyl ring of the 2-benzylpyridine moiety situated in the trans position with respect to the phenyl substituent of the 2-phenylisoquinoline moiety [N(2)–Ir–C(1) = 169.59(10)°], and the latter trans disposed to the phenyl substituent of the 3b ligand [N(1)–Ir–C(29) = 174.26(10)°].

The regioselective formation of **6a** must be highlighted. Homoleptic emitters bearing the N-heterocycles in the fac position are usual since this disposition appears to afford the most stable isomer.^25^ However, the heteroleptic emitters of the class [3b + 3b + 3b′] with the N-heterocycles disposed in position fac are very scarce,^4a,26^ most probable because the N-heterocycles are trans disposed in the starting compounds, [Ir(μ-Cl)(3b)_2_]_2_, and once the kinetically favored mer-emitters are formed, their mer–fac isomerization has too high activation energy. In this context, it should be noted that six-coordinate iridium(III) complexes exhibit a high octahedral Δ_0_ splitting.^27^ Thus, the ligand-field stabilization energy makes these emitters inert toward processes initiated by ligand dissociation reactions. As far as we know, heteroleptic emitters [3b + 3b′ + 3b″] bearing three different bidentate units with N-heterocycles fac disposed are unknown until now.

### Pyridyl-Benzyl Position Exchange in the Tetradentate Ligand

We have previously reported that the acetonitrile-solvate cation [Ir{κ^4^-*C*,*C*,*C*′,*C*′-[C_6_H_4_Im(CH_2_)_4_ImC_6_H_4_]}(CH_3_CN)_2_]^+^ (Im = imidazolylidene) facilitates the pyridyl-supported heterolytic *ortho*-CH bond activation of the phenyl group of 2-phenylpyridines to yield the corresponding [6tt + 3b] emitters using a base such as (piperidinomethyl)polystyrene. This bis(solvento) cation was prepared by abstraction of the iodide ligand of the dimer [Ir(μ-I){κ^4^-*C*,*C*,*C*′,*C*′-[C_6_H_4_Im(CH_2_)_4_ImC_6_H_4_]}]_2_ with a silver salt in acetone–dichloromethane, followed by the addition of acetonitrile.^7c^ This precedent encouraged us to extend the methodology to [6tt′ + 3b] emitters of heteroleptic tetradentate ligands, with the aim of comparing the stereochemistry of the formed compounds with that generated through Scheme 3.

**Scheme 4:**
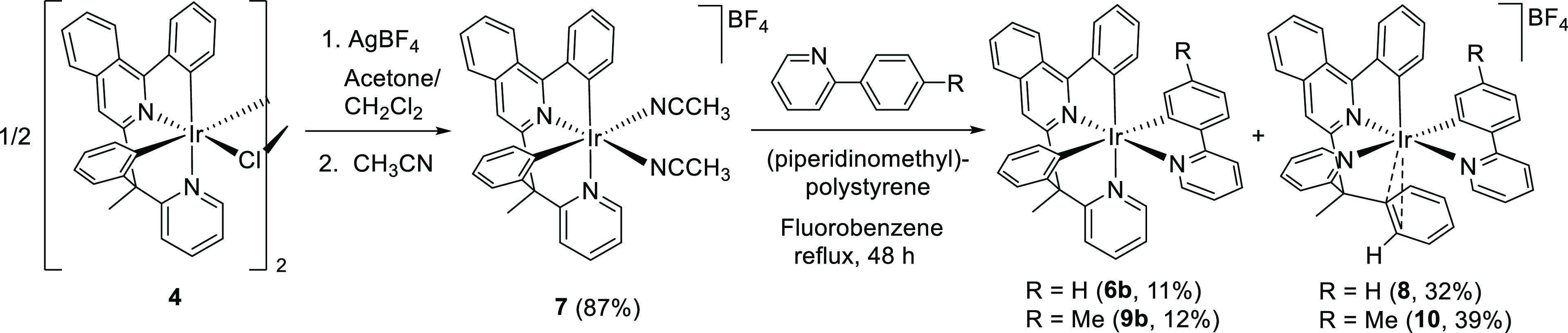
Preparation of Complexes **6b**, **7**, **8**, **9b**, and **10**

The same procedure starting from **4** affords the salt [Ir(κ^4^-*cis*-*C*,*C*′-*cis*-*N*,*N*′-MeL)(CH_3_CN)_2_]BF_4_ (**7**), which was isolated as an orange solid in 87% yield. The presence of two inequivalent acetonitrile ligands in the cation is supported by the ^1^H and ^13^C{^1^H} NMR spectra of the solid in dichloromethane-*d*_2_. The first spectrum displays two singlets at 2.81 and 2.11 ppm corresponding to the methyl groups, whereas the second one displays two singlets at 118.4 and 118.1 ppm due to C(sp)–carbon atoms and two singlets at 4.7 and 3.5 ppm for the methyl groups. Although complex **7** is a 6tt′-counterpart of the cation [Ir{κ^4^-*C*,*C*,*C*′,*C*′-[C_6_H_4_Im(CH_2_)_4_ImC_6_H_4_]}(CH_3_CN)_2_]^+^, they do not display the same behavior (Scheme 4). Treatment of fluorobenzene solutions of **7** with 1.0 equiv of 2-phenylpyridine in the presence of (piperidinomethyl)polystyrene under reflux for 48 h leads to a mixture of the mer isomer *mer*-Ir(κ^4^-*cis*-*C*,*C*′-*cis*-*N*,*N*′-MeL){κ^2^-*C*,*N*-(C_6_H_4_-py)} (**6b**) and the salt [Ir(κ^3^-*C*,*N*,*N*′;η^2^-*C*,*C*)-MeHL)(κ^2^-*C*,*N*-C_6_H_4_-py)]BF_4_ (**8**). Under the same conditions, 2-(*p*-tolyl)pyridine affords the mixture of the analogous-*p*-tolyl compounds: the mer isomer Ir(κ^4^-*cis*-*C*,*C*′-*cis*-*N*,*N*′-MeL){κ^2^-*C*,*N*-(C_6_H_3_Me-py)} (**9b**) and the salt [Ir(κ^3^-*C*,*N*,*N*’;η^2^-*C*,*C*)-MeHL)(κ^2^-*C*,*N*-C_6_H_3_Me-py)]BF_4_ (**10**). In the absence of the base, using propan-2-ol under reflux as a solvent, salts **8** and **10** were selectively formed.

**Figure 4:**
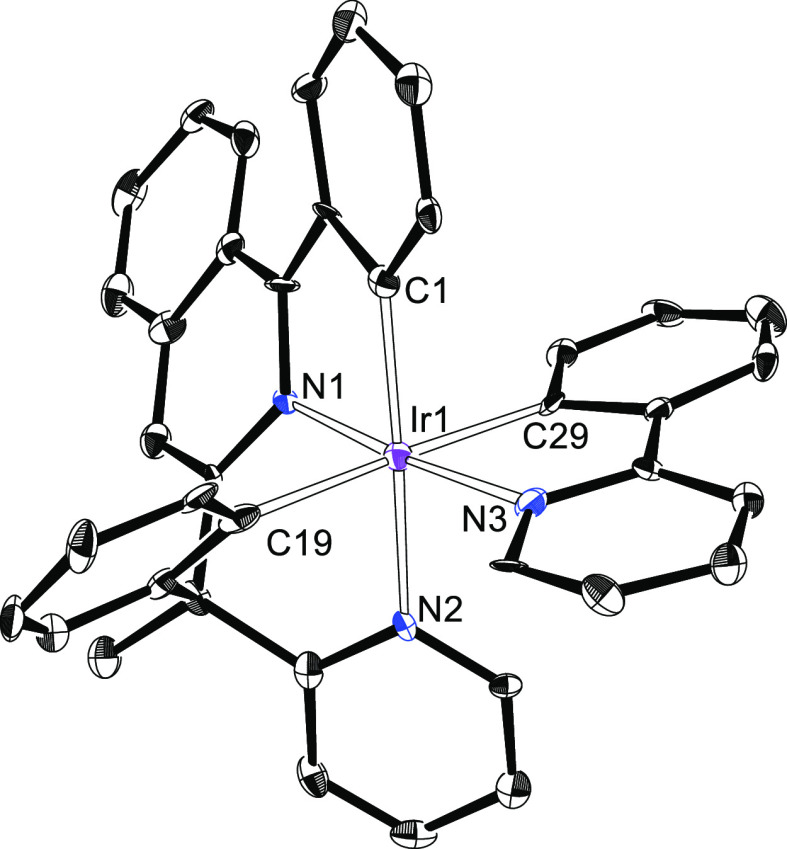
X-ray structure of one of the two independent molecules of **6b** showing 50% thermal ellipsoid probability (hydrogen atoms have been omitted). Selected bond lengths (Å) and angles (degree) for both molecules: Ir(1)–C(1) = 1.993(9), 1.996(9), Ir(1)–C(19) = 2.096(9), 2.118(9), Ir(1)–C(29) = 2.080(9), 2.071(9), Ir(1)–N(1) = 1.984(7), 1.956(8), Ir(1)–N(2) = 2.126(7), 2.138(7), Ir(1)–N(3) = 2.072(7), 2.063(7); N(1)–Ir(1)–N(3) = 175.0(3), 175.6(3), N(2)–Ir(1)–C(1) = 170.1(3), 171.1(3), C(19)–Ir(1)–C(29) = 177.8(4), 178.9(3).

**Figure 5:**
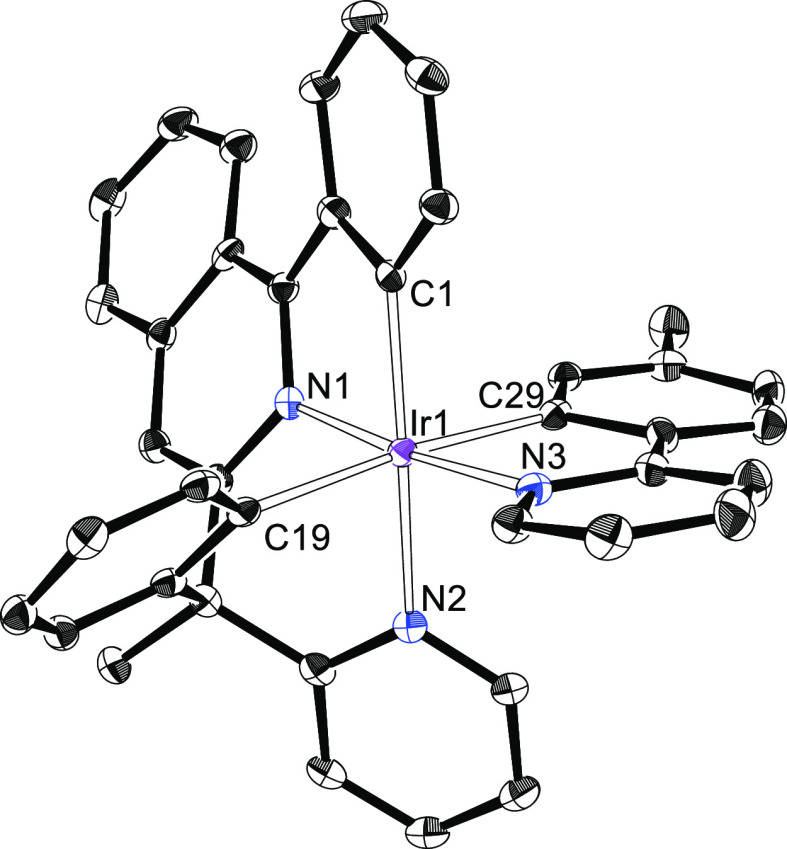
X-ray structure of one of the two independent molecules of **9b** showing 50% thermal ellipsoid probability (hydrogen atoms have been omitted). Selected bond lengths (Å) and angles (degree) for both molecules: Ir(1)–C(1) = 1.994(3), 1.998(3), Ir(1)–C(19) = 2.098(3), 2.085(3), Ir(1)–C(29) = 2.083(3), 2.098(3), Ir(1)–N(1) = 1.996(3), 2.000(3), Ir(1)–N(2) = 2.124(3), 2.126(3), Ir(1)–N(3) = 2.073(3), 2.061(3); N(1)–Ir(1)–N(3) = 174.27(11), 175.49(10), N(2)–Ir–C(1) = 171.28(12), 170.79(12), C(19)–Ir(1)–C(29) = 173.84(12), 176.32(12).

Complexes **6b** and **9b** were separated from the respective mixtures by basic-alumina column chromatography, employing dichloromethane as the eluent, and isolated as dark-red solids in 11 and 12% yield, respectively. Both compounds were characterized by X-ray diffraction analysis. Their structures demonstrate the mer disposition of the N-heterocycles and reveal that the arrangement of the donor atoms of the tetradentate ligand at the metal coordination sphere does not change with respect to that observed in **3**, **5**, and **6a**. A view of one of the two chemically equivalent but crystallographically independent molecules of **6b** and **9b**, which are present in the respective asymmetric units, is provided in Figures 4 and 5. For both compounds, the polyhedron around the iridium center can be described as a distorted octahedron with the pyridyl ring of the 3b ligand trans disposed to the isoquinolyl ring [N(1)–Ir(1)–N(3) = 175.0(3) and 175.6(3)° (**6b**), 174.27(11) and 175.49(10)° (**9b**)], the pyridyl group of the 2-benzylpyridine fragment situated in the trans position with respect to the phenyl substituent of the 2-phenylisoquinoline moiety [N(2)–Ir(1)–C(1) = 170.1(3) and 171.1(3)° (**6b**), 171.28(12) and 170.79(12)° (**9b**)], and phenyl unit of the benzyl group trans disposed to the phenyl substituent of the 3b ligand [C(19)–Ir(1)–C(29) = 177.8(4) and 178.9(3)° (**6b**), 173.84(12) and 176.32(12)° (**9b**)].

**Figure 6:**
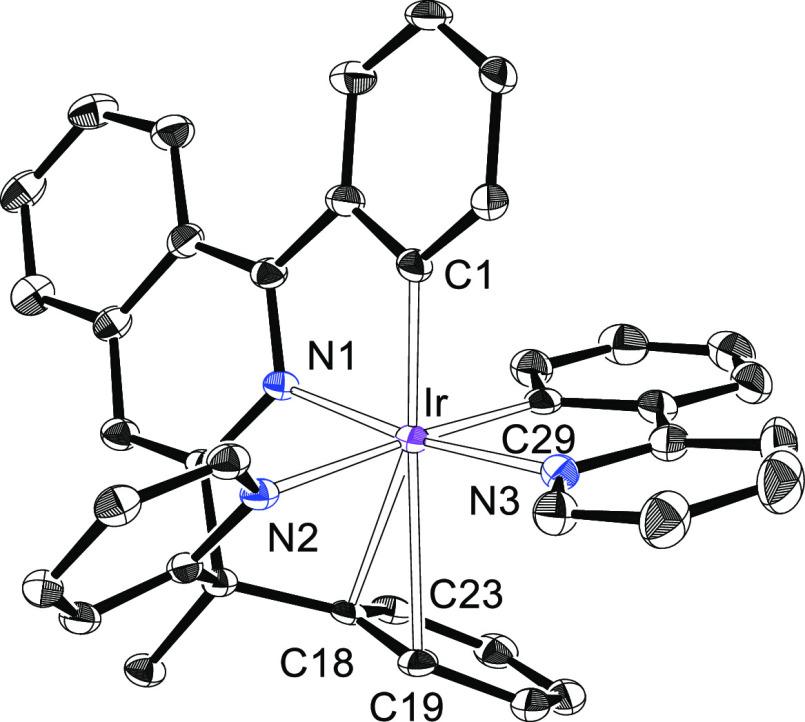
X-ray structure of the cation of **8** showing 50% thermal ellipsoid probability (hydrogen atoms have been omitted). Selected bond lengths (Å) and angles (degree): Ir–C(1) = 1.987(3), Ir–C(18) = 2.443(3), Ir–C(19) = 2.559 (3), Ir–C(23) = 3.225(3), Ir–C(29) = 2.012(3), Ir–N(1) = 2.016(3), Ir–N(2) = 2.144(3), Ir–N(3) = 2.063(3); N(2)–Ir–C(29) = 175.07(12), N(3)–Ir–N(1) = 172.17(11), C(1)–Ir–C(18) = 155.22(13), C(1)–Ir–C(19) = 171.53(13).

Cations of salts **8** and **10** are the result of a hydrogen-transfer reaction at the metal coordination sphere from the aryl substituent of the incoming pyridine ligand to the phenyl unit of the benzyl group of the tetradentate ligand. In addition, a position exchange between the pyridyl and phenyl rings of the 2-benzylpyridine moiety takes place; that is, in contrast to the previous complexes of this work, the phenyl substituent of the 2-phenylisoquinoline moiety and the phenyl unit of the benzyl group are mutually trans disposed. Both features were confirmed by the X-ray diffraction structure of the cation of **8** (Figure 6). Furthermore, the structure reveals that the incoming pyridyl ring coordinates trans to the isoquinolyl group [N(3)–Ir–N(1) = 172.17(11)°] as in mer isomers **6b** and **9b**. Thus, the octahedral environment of the iridium center is completed with the phenyl substituent of the 3b ligand trans disposed to the pyridyl ring of the 2-benzylpyridine moiety. The η^2^ coordination of the phenyl ring of the benzyl group to the iridium atom is strongly supported by the bond lengths Ir–C(18) and Ir–C(19) of 2.443(3) and 2.559(3) Å, *d*_1_ and *d*_2_, respectively, and the Ir–C(23) separation of 3.225 Å (*d*_3_). It has been proposed that to calibrate low hapticities of coordinated arene ligands, the three shortest M–C distances, *d*_1_ < *d*_2_ < *d*_3_, should be analyzed via the ρ_1_ and ρ_2_ parameters (eqs 1 and 2). For an η^2^ coordination, it is fulfilled that *d*_1_ ≈ *d*_2_ < *d*_3_, and therefore, ρ_2_ > ρ_1_ ≈ 1.^28^ For **8**, the calculated ρ_2_ and ρ_1_ values are 1.32 and 1.05, respectively, in agreement with that observed in the few previously reported Ir(η^2^-arene) complexes.^29^
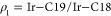
1
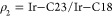
2

**Scheme 5:**
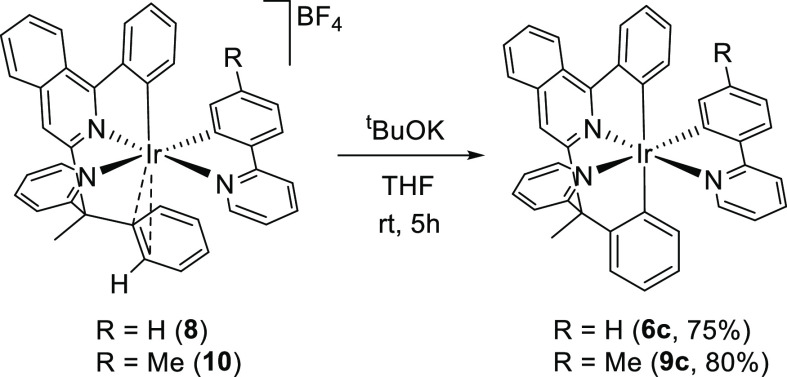
Preparation of Complexes **6c** and **9c**

The metal center of **8** and **10** increases the acidity of the *ortho*-hydrogen atom of the coordinated C–C double bond as a result of a transference of electrophilicity, which makes it quite acidic. Thus, the treatment of the THF solutions of the salts with 4.0 equiv of KO^*t*^Bu at room temperature for 5 h causes its abstraction and the formation of the respective [6tt′ + 3b] isomers **6c** and **9c** (Scheme 5), which were isolated as dark-red solids in 75 and 80% yield, respectively.

**Figure 7:**
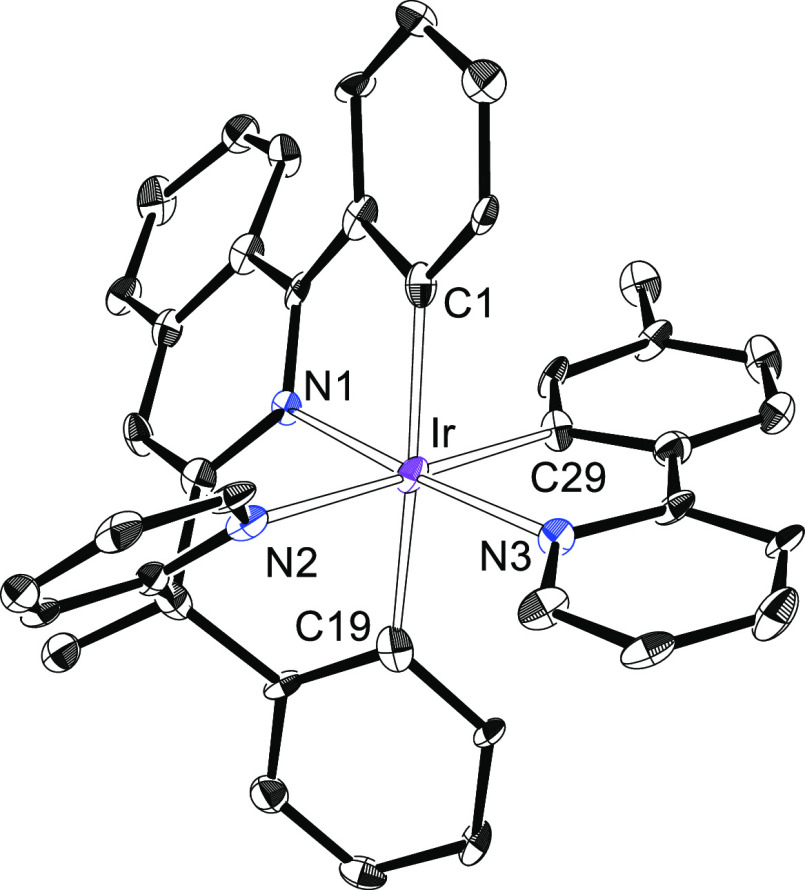
X-ray structure of **9c** showing 50% thermal ellipsoid probability (hydrogen atoms have been omitted). Selected bond lengths (Å) and angles (degree): Ir–C(1) = 2.079(10), Ir–C(19) = 2.078(10), Ir–C(29) = 2.026(9), Ir–N(1) = 2.020(7), Ir–N(2) = 2.140(8), Ir–N(3) = 2.060(7); N(1)–Ir–N(3) = 176.0(3), C(29)–Ir–N(2) = 178.3(4), C(1)–Ir–C(19) = 169.8(4).

The hydrogen abstraction is a stereochemically clean process, which does not modify the mer disposition observed for the N-heterocycles of the cation, as proved by the X-ray structure of **9c** (Figure 7). Thus, the coordination polyhedron around the iridium atom of these other mer isomers can be seen as a distorted octahedron with the isoquinolyl group trans disposed to the pyridyl ring of the 3b ligand [N(1)–Ir–N(3) = 176.0(3)°], whereas the phenyl substituent of the latter lies trans to the pyridyl ring of the 2-benzylpyridine moiety [C(29)–Ir–N(2) = 178.3(4)°]. The phenyl groups of the tetradentate ligand are also mutually trans disposed [C(1)–Ir–C(19) = 169.8(4)°].

Isomers **a–c** of these [6tt′ + 3b] emitters are kinetically inert, and isomerization between them is not observed in toluene, at reflux, after days. This is consistent with the previously mentioned inertia of the iridium(III) octahedral complexes.

### Photophysical and Electrochemical Properties of the New Emitters

Figures S1–S9 show the UV–vis spectra of 2-methyltetrahydrofuran (2-MeTHF) 1 × 10^–4^ M solutions of complexes **3**, **5**, **6a–c**, **8**, **9b,c**, and **10**. To their rationalization, time-dependent density functional theory (DFT) (TD-DFT) calculations [B3LYP-GD3//SDD(f)/6-31G**]^5,7c,10^ were also carried out, considering THF as a solvent. Selected absorptions are listed in Tables 1 and S1–S18, whereas frontier molecular orbitals are given in Figures S10–S19 and Tables S19–S27. The HOMO spreads out over the metal center (30–50%), the phenyl groups of the tetradentate ligand (25–50%), and the orthometalated 2-arylpyridine ligand (10–25%) for isomers **6** and **9** and cations **8** and **10**. The LUMO is located on the phenylisoquinoline moiety, about 70% on the heterocycle and close to 20% on the phenyl substituent.

**Table 1:** Summary of UV–vis Absorption Data for Complexes **3**, **5**, **6a–c**, **8**, **9b,c**, and **10** (in 2-MeTHF) and Computed TD-DFT (in THF) Vertical Excitation Energies

λ_exp_ (nm)	ε (M^–1^ cm^–1^)	excitation energy (nm)	oscillator strength (*f*)	transition	assignment
Complex **3**					
265	6708	262	0.118	HOMO – 9 → LUMO (31%), HOMO – 3 → LUMO + 2 (17%)	6tt′ + Cl → 6tt′
365	1700	379	0.0894	HOMO – 1 → LUMO (92%)	Ir + 6tt′ → 6tt′
416	8140	424 (S_1_)	0.0565	HOMO → LUMO (96%)	Ir + 6tt′ → 6tt′
550	90	549 (T_1_)	0	HOMO → LUMO (44%)	Ir + 6tt′ → 6tt′
				HOMO – 1 → LUMO (42%)	
Complex **5**					
242	31060	264	0.1216	HOMO – 9 → LUMO (46%)	6tt′ → 6tt′
442	3740	449	0.0782	HOMO – 1 → LUMO (82%)	Ir + 6tt′ + acac → 6tt′
484	2900	491 (S_1_)	0.0205	HOMO → LUMO (84%)	Ir + 6tt′ → 6tt′
600	170	606 (T_1_)	0	HOMO – 1 → LUMO (72%)	Ir + 6tt′ + acac → 6tt′
Complex **6a**					
267	7930	288	0.095	HOMO – 4 → LUMO (51%)	6tt′ + 3b → 6tt′ + 3b
435	1540	447	0.0564	HOMO – 1 → LUMO (89%)	Ir + 6tt′ + 3b → 6tt′
503	350	515 (S_1_)	0.0044	HOMO → LUMO (98%)	Ir + 6tt′ + 3b → 6tt′
590	70	590 (T_1_)	0	HOMO → LUMO (48%)	Ir + 6tt′ + 3b → 6tt′
				HOMO – 2 → LUMO (24%)	
Complex **6b**					
260	7055	266	0.0289	HOMO – 7 → LUMO + 2 (49%)	6tt′ + 3b → 6tt′
390	903	403	0.0491	HOMO – 2 → LUMO (93%)	Ir + 6tt′ + 3b → 6tt′
502	140	511 (S_1_)	0.0304	HOMO → LUMO (98%)	Ir + 6tt′ → 6tt′
574	33	593 (T_1_)	0	HOMO → LUMO (45%)	Ir + 6tt′ → 6tt′
				HOMO – 1 → LUMO (37%)	
Complex **6c**					
251	20000	267	0.0619	HOMO – 8 → LUMO + 2 (45%)	6tt′ + 3b → 6tt′+3b
399	5100	406	0.0932	HOMO – 2 → LUMO (89%)	Ir + 6tt′ → 6tt′
503	840	533 (S_1_)	0.0092	HOMO → LUMO (98%)	Ir + 6tt′ → 6tt′
600	220	608 (T_1_)	0	HOMO → LUMO (59%)	Ir + 6tt′ → 6tt′
				HOMO – 2 → LUMO (32%)	
Complex **8**					
254	10225	254	0.0107	HOMO – 6 → LUMO + 2 (54%)	6tt′ + 3b → 6tt′+3b
375	1860	387	0.0801	HOMO – 1 → LUMO (85%)	Ir + 3b → 6tt′
460	530	479 (S_1_)	0.0274	HOMO → LUMO (95%)	Ir + 3b → 6tt′
566	65	572 (T_1_)	0	HOMO → LUMO (53%)	Ir + 3b → 6tt′
				HOMO – 1 → LUMO (36%)	
Complex **9b**					
262	8104	268	0.016	HOMO – 7 → LUMO + 2 (56%)	6tt′ + 3b → 6tt′
394	1332	405	0.0517	HOMO – 2 → LUMO (92%)	Ir + 6tt′ + 3b → 6tt′
501	236	514 (S_1_)	0.0293	HOMO → LUMO (98%)	Ir + 6tt′ → 6tt′
569	84	594 (T_1_)	0	HOMO → LUMO (47%)	Ir + 6tt′ → 6tt′
				HOMO – 1 → LUMO (35%)	
Complex **9c**					
253	10440	267	0.0672	HOMO – 8 → LUMO + 2 (72%)	6tt′ + 3b → 6tt′ + 3b
410	1960	407	0.0868	HOMO – 2 → LUMO (86%)	Ir + 6tt′ → 6tt′
526	230	537 (S_1_)	0.0087	HOMO → LUMO (98%)	Ir + 6tt′ → 6tt′
590	60	611 (T_1_)	0	HOMO → LUMO (59%)	Ir + 6tt′ → 6tt′
				HOMO – 2 → LUMO (32%)	
Complex **10**					
233	16120	263	0.0513	HOMO – 8 → LUMO + 1 (47%)	6tt′ + 3b → 6tt′+3b
387	2420	387	0.0732	HOMO – 1 → LUMO (84%)	Ir + 3b → 6tt′
490	620	483 (S_1_)	0.0277	HOMO → LUMO (94%)	Ir + 3b → 6tt′
560	280	573 (T_1_)	0	HOMO → LUMO (53%)	Ir + 3b → 6tt′
				HOMO – 1 → LUMO (36%)	

The spectra can be properly analyzed by means of their division in three different regions: < 300, 300–550, and >550 nm. The absorptions at the highest energy region are assignable to π–π* intra- and interligand transitions. Bands between 300 and 500 nm result from spin-allowed metal to ligand charge transfer along with intraligand and ligand to ligand charge transfer. The very weak absorption tails after 550 nm are ascribed to formally spin forbidden transitions, mainly HOMO-to-LUMO and HOMO – 1-to-LUMO (**3**, **5**, **6b**, **8**, **9b**, and **10**) or HOMO – 2-to-LUMO (**6a**, **6c**, and **9c**), caused by the large spin–orbit coupling associated with the metal ion.

The redox properties of complexes **3**, **5**, **6a–c**, **8**, **9b**,**c**, and **10** were also evaluated by cyclic voltammetry to obtain more information on their frontier orbitals. Oxidation and reduction potentials were measured under an argon atmosphere in acetonitrile solutions, and the potentials were referenced versus Fc/Fc^+^. Figure S20 provides the cyclic voltammetry traces, whereas Table 2 lists the potential values. The table also includes the HOMO energy levels estimated from the oxidation potentials and LUMO estimated from both the reduction potential and the optical gap obtained from the onset of emission, as well as DFT-calculated values.

**Table 2:** Electrochemical and DFT MO Energy Data for Complexes **3**, **5**, **6a–c**, **8**, **9b,c**, and **10**

			obs (eV)				calcd (eV)		
complex	*E*^ox^ (V)	*E*^red^ (V)	HOMO^a^	LUMO^b^	*E*_00_^c^	LUMO from *E*_00_	HOMO	LUMO	HLG
**3**	1.13	–1.93	–5.93	–2.87	2.17	–3.76	–5.70	–2.09	3.61
**5**	0.42^d^		–5.22		1.95	–3.27	–5.06	–1.79	3.27
**6a**	0.27^d^		–5.07		1.99	–3.08	–4.90	–1.77	3.13
**6b**	0.21	–2.28	–5.01	–2.52	2.00	–3.01	–4.91	–1.75	3.16
**6c**	0.11	–2.18	–4.91	–2.62	1.97	–2.94	–4.87	–1.82	3.05
**8**	1.04	–1.88, −2.42	–5.84	–2.92	2.13	–3.71	–5.73	–2.38	3.35
**9b**	0.20	–2.28	–5.00	–2.52	2.00	–3.00	–4.88	–1.74	3.14
**9c**	0.09	–2.19	–4.89	–2.61	1.97	–2.92	–4.85	–1.81	3.04
**10**	1.01	–1.88, −2.40	–5.81	–2.92	2.13	–3.68	–5.69	–2.37	3.32

a HOMO = −[*E*^ox^ vs Fc/Fc^+^ + 4.8] eV.

b LUMO = −[*E*^red^ vs Fc/Fc^+^ + 4.8] eV.

c *E*_00_ = onset of emission in THF at 77 K.

d *E*_1/2_^ox^.

All compounds exhibit an Ir(III)/Ir(IV) oxidation peak. The nature of the process and the potential value depend upon the compound class and its stereochemistry. The oxidation of carbonyl derivative **3** is irreversible and takes place at 1.13 V. Cations **8** and **10** also undergo irreversible oxidation at slight lower potentials, 1.01 and 1.04 V, respectively. In contrast, the oxidation of acac-derivative **5** is reversible with *E*_1/2_^ox^ = 0.42 V. The oxidation potential of the 2-phenylpyridine-type compounds **6a–c** and **9b**,**c** is between 0.09 and 0.27 V, being reversible for fac isomer **6a** (*E*_1/2_^ox^ = 0.27 V) and irreversible for the rest. The irreversible character of the oxidation could be associated to some structural change in the resulting unsaturated d^5^-species. It should be noted that the trigonal prism is a usual polyhedron for unsaturated six-coordinated compounds;^30^ nevertheless, it could be also a simple distortion of the original octahedron. These complexes do not degrade in the cyclic voltammetry experiments. Multiple scans provide similar voltammograms with a slight decrease of the oxidation peak intensity, which is generally due to adsorption of the compound on the electrode (Figure S21). Carbonyl complex **3** displays irreversible reduction at −1.93 V, whereas cations **8** and **10** undergo two irreversible reductions at about −2.41 and −1.88 V. Mer isomers **6b**,**c** and **9b**,**c** show an irreversible reduction between −2.18 and −2.28 V. On the other hand, reduction is not observed for acac compond **5** and fac isomer **6a**. Both the experimental and DFT-calculated HOMO–LUMO gaps decrease in the sequence of **3** > **8** ≈ **10** > **5** > **6b** ≈ **9b** ≈ **6a** > **6c** ≈ **9c**.

Complexes **3**, **5**, **6a–c**, **8**, **9b,c**, and **10** are red phosphorescent emitters (601–732 nm) when photoexcited in a doped poly(methylmethacrylate) (PMMA) film at 5 wt % at room temperature and 2-methyltetrahydrofuran at room temperature and at 77 K (Figures S23–S49). Table 3 gathers the experimental and calculated wavelengths, observed lifetimes, quantum yields, and radiative and nonradiative rate constants.

**Table 3:** Emission Data for Complexes **3**, **5**, **6a–c**, **8**, **9b,c**, and **10**

complex	calcd λ_em_ (nm)	media (*T*/K)	λ_em_ (nm)	τ (μs)	Φ	*k*_r_^a^ (s^–1^)	*k*_nr_^a^ (s^–1^)	*k*_r_/*k*_nr_
**3**	626	PMMA (298)	645	1.4	0.08	5.7 × 10^4^	6.6 × 10^5^	0.1
		2-MeTHF (298)	645	2.6	0.13	5.0 × 10^4^	3.4 × 10^5^	0.2
		2-MeTHF (77)	601, 647	3.8				
**5**	691	PMMA (298)	681	0.7	0.57	8.1 × 10^5^	6.1 × 10^5^	1.3
		2-MeTHF (298)	682	0.8	0.58	7.4 × 10^5^	5.3× 10^5^	1.4
		2-MeTHF (77)	665, 715	1.2				
**6a**	672	PMMA (298)	679, 720	0.9	0.17	1.9 × 10^5^	9.2 × 10^5^	0.2
		2-MeTHF (298)	676	1.5	0.25	1.7 × 10^5^	5.0 × 10^5^	0.3
		2-MeTHF (77)	650, 701	1.8				
**6b**	677	PMMA (298)	663	1.2	0.29	2.4 × 10^5^	5.9 × 10^5^	0.4
		2-MeTHF (298)	668	2.3	0.22	9.6 × 10^4^	3.4 × 10^5^	0.3
		2-MeTHF (77)	646, 699					
**6c**	699	PMMA (298)	694, 715	4.6	0.16	3.5 × 10^4^	1.8 × 10^5^	0.2
		2-MeTHF (298)	692	2.0	0.12	0.6 × 10^4^	4.4 × 10^5^	0.1
		2-MeTHF (77)	668, 709	1.3				
**8**	645	PMMA (298)	669	1.4	0.18	1.3 × 10^5^	5.9 × 10^5^	0.2
		2-MeTHF (298)	663	1.6	0.17	1.1 × 10^5^	5.2 × 10^5^	0.2
		2-MeTHF (77)	617, 647	2.7				
**9b**	676	PMMA (298)	663	1.6	0.23	1.4 × 10^5^	4.8 × 10^5^	0.3
		2-MeTHF (298)	668	1.5	0.38	2.5 × 10^5^	4.1 × 10^5^	0.6
		2-MeTHF (77)	649, 698	2.2				
**9c**	695	PMMA (298)	681	2.1	0.13	6.2 × 10^4^	4.1 × 10^5^	0.2
		2-MeTHF (298)	732	1.4	0.14	1.0 × 10^5^	6.1 × 10^5^	0.2
		2-MeTHF (77)	671, 729					
**10**	652	PMMA (298)	678	1.2	0.16	1.3 × 10^5^	7.0 × 10^5^	0.2
		2-MeTHF (298)	666	1.8	0.19	1.1 × 10^5^	4.5 × 10^5^	0.2
		2-MeTHF (77)	608, 652	3.6				

a Calculated according to *k*_r_ = ϕ/τ and *k*_nr_ = (1 – ϕ)/τ.

**Figure 8:**
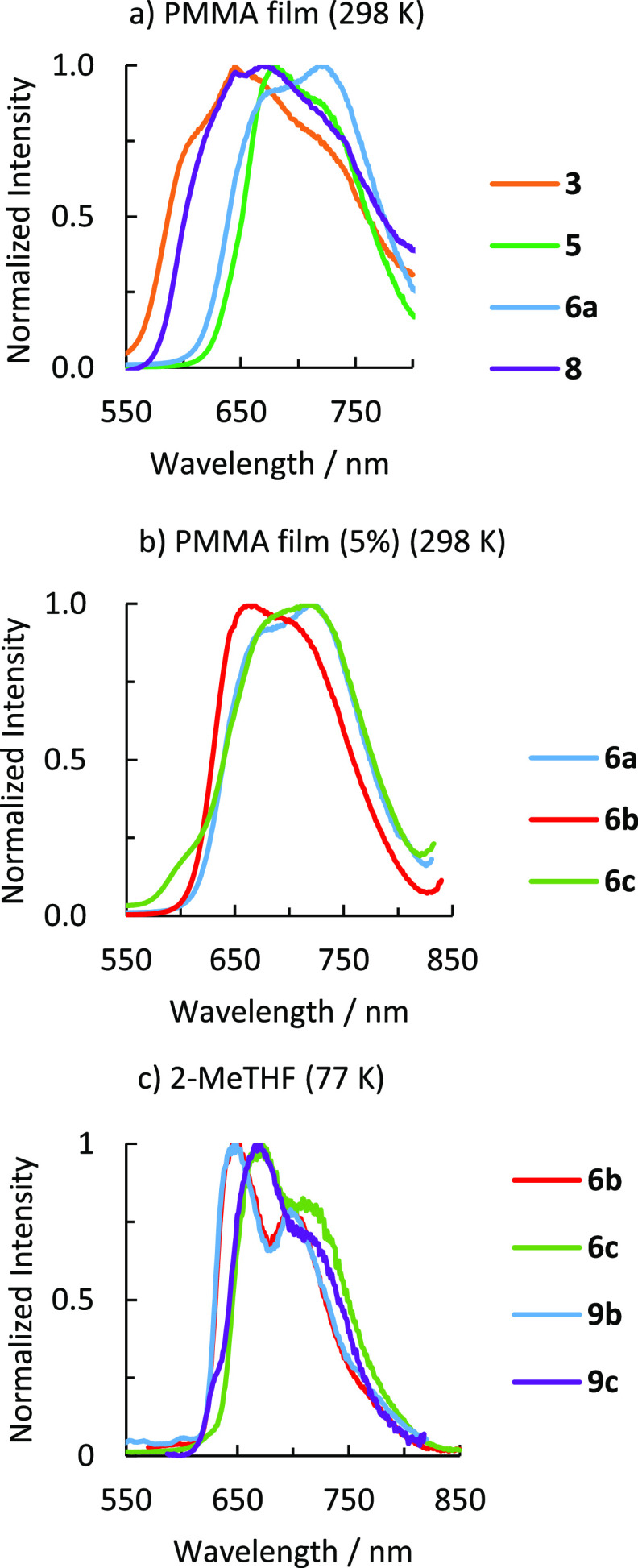
(a) Emission spectra of **3**, **5**, **6a**, and **8** in 5 wt % PMMA films at 298 K. (b) Emission spectra of isomers **6a−c** in 5 wt % PMMA films at 298 K. (b) Emission spectra of **6b,c**, and **9b,c** in 2-Me-THF at 77K.

There is good agreement between the experimental wavelengths and those obtained by estimating the difference in energy between the optimized triplet states T_1_ and the singlet states S_0_ in THF, suggesting that the emissions can be ascribed to T_1_ excited states. The emission depends upon the chemical nature of the emitter and the stereochemistry of the isomer (Figure 8). Thus, the wavelength of the emission maximum is slightly orange-shifted in the sequence of **6** ≈ **5** < **8** < **3**, in good agreement with the observed increase of the HOMO–LUMO gap; that is, phenylpyridine complexes ≈ acac derivative < cationic species < carbonyl compound (Figure 8a). The emission maximum of isomers **b** also undergoes orange shift with regard to those of isomers **a** and **c** (Figure 8b,c).

In contrast, the incorporation of a methyl substituent at the phenyl group of the 3b phenylpyridine ligand does not affect the wavelength of the emission (Figure 8c). A similar result has been observed for complexes Ir(acac){κ^2^-*C*,*N*-(C_6_RH_3_-py)}{κ^2^-*C*,*N*-(C_6_H_4_-py)} (R = Me, Ph), which display almost identical emissions despite the different substitution at the phenyl group of one of the orthometalated 2-phenylpyridine ligand.^4d^ The lifetimes are short and lie in a narrow range of 0.7–4.6 μs. The quantum yields also depend upon the chemical nature of the emitter. Those of acac derivative **5** are particularly noticeable, about 0.60 in both 5 wt % PMMA film and 2-methyltetrahydrofuran, which compare well with the quantum yields reported for complex Ir{κ^2^-*C*,*N*-(C_6_H_4_-isoqui)}_2_{κ^2^-*O*,*O*-[OC(CO_2_CH_3_)CHC(OCH_3_)O]}, bearing two orthometalated 2-phenyl-isoquinoline ligands and an asymmetrical acac group with an electron-acceptor carboxylate and an electron-donor methoxy as substituents at the carbonyl groups.^13k^ Salts **8** and **10** as well as the isomers of the 2-arylpyridine derivatives **6** and **9** display quantum yields in the range of 0.38–0.12. This significant decrease appears to be a consequence of the decrease of the radiative constant as a result of the replacement of the acac group with the C,N-donor ligand.

### Electroluminescence Properties of an OLED Device Based on 5

**Figure 9:**
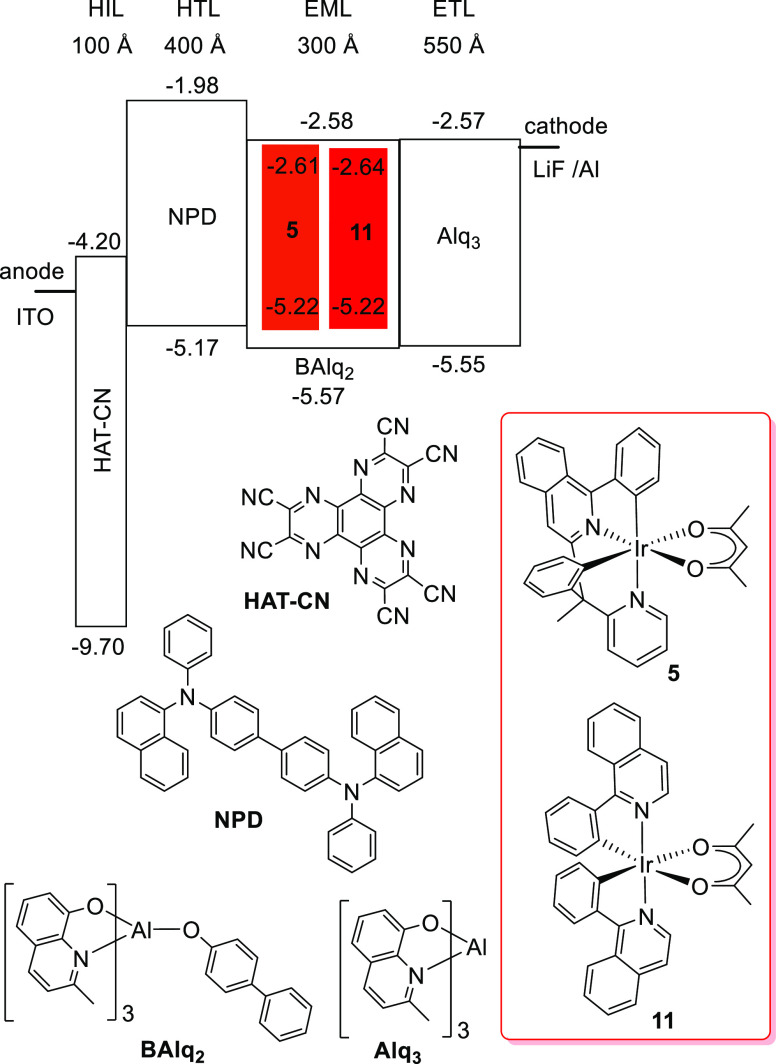
Device structure, energy levels (eV), and molecular structures of the materials used.

Since acac derivative **5** exhibits the highest photoluminescence quantum yield of the prepared [6tt′ + 3b] compounds, we decided to evaluate it as an emitter in a PhOLED device and to compare it with one based on the known [3b + 3b + 3b′] red emitter Ir{κ^2^-*C*,*N*-(C_6_H_4_-isoqui)}_2_(acac) (**11**).^13a−13c,13f−13h,13l^ The emitters have been tested in bottom emission OLED structures. The devices were fabricated by high-vacuum thermal evaporation. The anode was 1150 Å of indium tin oxide (ITO). The cathode comprised 10 Å of LiF, followed by 1000 Å of aluminum. The organic stack of the devices consisted of, sequentially from the anode, 100 Å of HAT-CN (dipyrazino[2,3-*f*:2′,3′-*h*]-quinoxaline-2,3,6,7,10,11-hexacarbonitrile) as the hole injection layer (HIL), 400 Å of NPD [*N*,*N*′-bis(naphthalen-1-yl)-*N*,*N*′-bis(phenyl)benzidine] as a hole-transporting layer (HTL), 300 Å of an emissive layer (EML) containing BAlq_2_ (bis(2-methyl-8-quinolinolate)-4-(phenylphenolato)aluminum) as a host doped with the red emitter (9%), and 550 Å of Alq_3_ as an electron-transporting layer (ETL). Red emitters **5** and **11** were compared side by side in the same structure. Figure 9 shows the schematic structure and energy levels of the devices and the molecular structures of the materials used.

**Figure 10:**
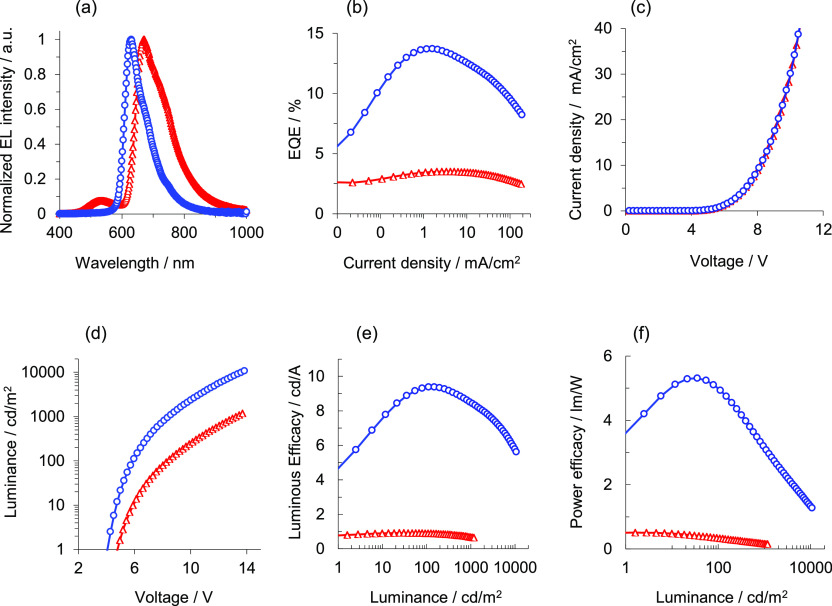
Performance of the devices based on complex **5** (red triangles) and 11 (blue circles): (a) EL spectra. (b) EQE vs *J*, (c) *J* vs *V*, (d) L vs *V*, (e) LE vs L, (f) PE vs L.

**Figure 11:**
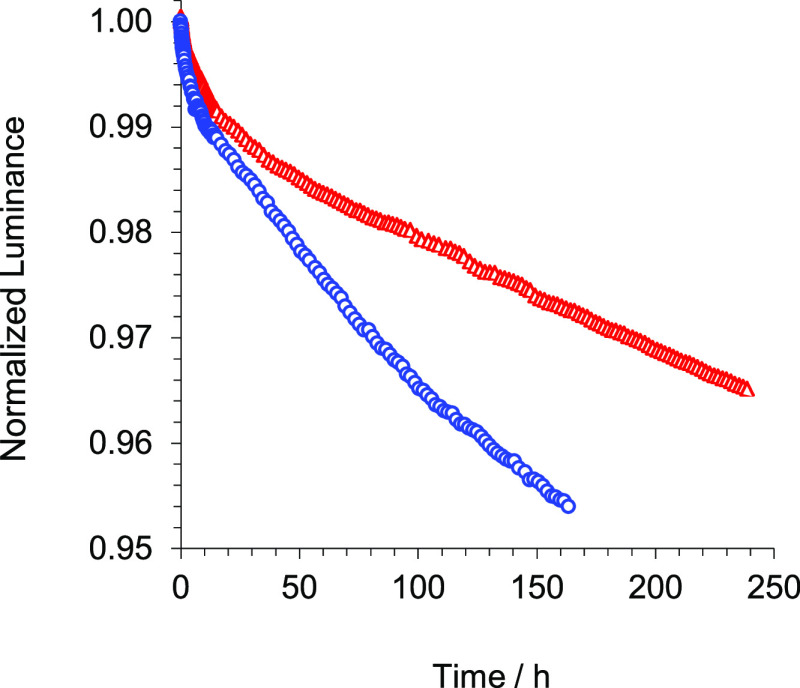
Normalized luminance of the devices based on complex **5** (red triangles) and **11** (blue circles) vs time at a constant current density of 80 mA/cm^2^.

The electroluminescence (EL) and current density–voltage–luminance (*J*–*V*–*L*) characteristics of the devices were tested upon manufacture. The performance data of both devices are shown in Table 4 and Figure 10. The EL spectrum of the device doped with **5** shows a peak at 672 nm, which is in agreement with its photoluminescence spectra. It is 42 nm red-shifted and 40 nm broader with respect to that of reference compound **11** (Figure 10a). The EL spectrum of the device based on **5** contains some emission with a peak wavelength of 530 nm. It can be attributed to emission form Alq_3_ ETL. Therefore, likely due to the fairly deep HOMO level of **5**, −5.22 eV, the holes cannot be trapped efficiently in the EML and can lead to Alq_3_ ETL causing some recombination and emission in this layer. An external quantum efficiency (EQE) of 3.4% at 10 mA/cm^2^ was achieved in the device with **5** as the emitter versus 12.4% for **11** (Figure 10b). Both devices display a very similar profile for current density (*J*) versus voltage (*V*) (Figure 10c). However, the brightness (*L*) is lower for the device doped with **5** compared to that of the device doped with **11** (Figure 10d). Due to the fact that a significant part of the **5** emission is outside the visible range (>780 nm), the luminance efficacy (LE, Figure 10e) and power efficacy (PE, Figure 10f) of this emitter device are expected to be low. Recent OLED devices based on [3b + 3b + 3b′] iridium emitters, with similar CIE coordinates, exhibit EQEs and brightnesses in the ranges of 0.2–31.2% and 288–48617 cd/m^2^, respectively.^5,31^ Both devices were life-tested at room temperature under accelerated conditions of a current density of 80 mA/cm^2^. The time at which luminance falls to 95% of its initial value, LT_95%_, at 10 mA/cm^2^ was calculated assuming an acceleration factor 2. As can be seen in Table 4 and Figure 11, the LT_95%_ at the same operating current density is notably higher for the OLED based on **5** (393 h for **5** vs 186 h for **11** at 80 mA/cm^2^). The LT_95%_ improvement of **5** with regard to **11** could be explained by the significantly lower exciton energy for **5**, which is red-shifted with regard to **11**. In this context, it should be noted that a higher-energy exciton causes more damage to the device.^32^

**Table 4:** EL Performance of the Devices Based on **5** and **11**

			1931 CIE		at 10 mA/cm^2^					at 80 mA/cm^2^	
emitter (9%)	λ_max_ (nm)	fwhm^a^ (nm)	CIE *x*	CIE *y*	voltage (V)	LE^b^ (cd/A)	EQE^c^ (%)	PE^d^ (lm/W)	LT_95%_^e^ (h)	*L*_0_^f^ (cd/m^2^)	LT_95%_^e^ (h)
**5**	672	118	0.556	0.390	8.2	0.9	3.4	0.3	17724	603	393
**11**	630	78	0.674	0.323	8.4	8.5	12.4	3.2	7522	5458	186

a Full width at half-maximum of the emission peak in the electroluminescence spectrum.

b Luminous efficacy.

c External quantum efficiency.

d Power efficacy.

e Lifetime as the time the luminance falls to 95% of its initial value.

f Initial luminance.

## Concluding Remarks

This study has shown that the new organic molecule 1-phenyl-3-(1-phenyl-1-(pyridine-2-yl)ethyl)isoquinoline, which was prepared by means of a palladium-catalyzed “*deprotonative cross*-*coupling process*”, reacts with the iridium-diolefin precursor [Ir(μ-Cl)(η^2^-COE)_2_]_2_ to afford an [Ir(μ-Cl)(6tt′)]_2_ dimer as a consequence of the orthometalation of the phenyl groups and the coordination of the N-heterocycles. This dimer allows to access iridium(III) red emitters of the type [6tt′ + 3b], which really are tris-heteroleptic [3b + 3b′ + 3b″], since the tetradentate 6tt′ ligand is certainly a [3b + 3b′] ensemble formed by two different units: an orthometalated 2-phenylisoquinoline and an orthometalated 2-benzylpyridine. The bidentate 3b donor is an acac group or an orthometalated 2-phenylpyridine-type ligand.

The link between the orthometalated 2-phenylisoquinoline and 2-benzylpyridine units reduces the number of possible stereoisomers of the structure [6tt′ + 3b] with respect to a [3b + 3b′ + 3b″] emitter bearing three free bidentate 3b units, and further, it permits a noticeable stereocontrol. Thus, from the four possible dispositions that are conceivable for a [3b + 3b′] ensemble formed by free 3b and 3b′ ligands such as an orthometalated 2-phenylisoquinoline and an orthometalated 2-benzylpyridine (phenyl-*trans*-pyridine, phenyl-*trans*-phenyl, phenyl-*trans*-isoquinoline, and pyridine-*trans*-isoquinoline), only the first two are observed for the 6tt′ ligand in the [6tt′ + 3b] emitters, with clearly the first of them being the most common. The phenyl-*trans*-phenyl disposition is generated from the phenyl-*trans*-pyridine one and involves a position exchange between the pyridyl and phenyl rings of the 2-benzylpyridine unit. The exchange is produced in reactions of a cationic solvate precursor [Ir(6tt′)S_2_]^+^ with 2-phenylpyridine-type molecules. These reactions involve a hydrogen transfer from the aryl substituent of the incoming pyridine ligand to the phenyl unit of the benzyl group of the tetradentate ligand on the metal coordination sphere. The hydrogen transfer affords an η^2^-arene synthetic intermediate, which yields the final product by deprotonation of the coordinated double bond of the arene. It occurs at a moderate temperature, about 80 °C, which favors the formation of mer isomers with the incoming heterocycle trans disposed to the isoquinoline moiety. At room temperature, the preparation of fac isomers [6tt′ + 3b] is also possible through direct substitution of the chloride anion of the dimer [Ir(μ-Cl)(6tt′)]_2_ with an orthometalated 2-phenylpyridine ligand.

The phosphorescent emitter resulting from the replacement of the chloride anion of the dimer [Ir(μ-Cl)(6tt′)]_2_ with an acac ligand, which displays a phenyl-*trans*-pyridine disposition for the tetradentate ligand, is notable, and its quantum yield of about 0.60 should be highlighted. Furthermore, it proves to have applicability to the fabrication of OLED devices. The OLED with such an emitter (λ_max_ 672 nm) revealed a 3.4% EQE at an operating current density of 10 mA/cm^2^.

In summary, here, we describe the synthesis of a new organic molecule that allows the preparation of red phosphorescent emitters of iridium(III), with three different bidentate units, and a better stereocontrol of the resulting structures. Its coordination to iridium, the synthesis of the emitters, their photophysical properties, and the applicability to the fabrication of OLED devices of one of them are also included as a proof-of-concept validation.

## Experimental Section

The starting compounds [Ir(μ-Cl)(η^4^-COD)]_2_ (**1**),^33^ [Ir(μ-Cl)(η^2^-COE)_2_]_2_ (**2**),^34^ and 3-chloro-1-phenylisoquinoline^35^ were prepared by published methods. Chemical shifts and coupling constants in the NMR spectra (Figures S50–S73) are given in ppm and Hz, respectively.

### Synthesis of 1-Phenyl-3-(phenyl(pyridin-2-yl)methyl)isoquinoline (H_2_L)

Lithium bis(trimethylsilyl)amide (1 M, 2.635 mL, 2.635 mmol) was added dropwise over 10 min to a mixture of palladium(II) acetate (14.09 mg, 0.063 mmol) and 4,6-bis(diphenylphosphino)phenoxazine (N-XantPhos, 34.6 mg, 0.063 mmol) in CPME (6 mL). 2-Benzylpyridine (201 μL, 1.255 mmol) was added to this reaction mixture, followed by 3-chloro-1-phenylisoquinoline (300 mg, 1.255 mmol) in 5 mL of CPME, and it was heated to 60 °C for 2 h. The reaction mixture was cooled to room temperature and quenched slowly with 3 M HCl/MeOH to a pH of 7. Then, it was concentrated, and the crude was purified by column chromatography (silica gel) using *n*-hexane/dichloromethane (gradient elution from 100 to 30% *n*-hexane). The pure fractions were combined and concentrated to give a foamy yellow solid (266.3 mg, 57%). HRMS (electrospray, *m*/*z*): calcd for C_27_H_21_N_2_ [M + H]^+^, 373.1699; found, 373.1682. ^1^H NMR NMR (400 MHz, CDCl_3_, 298 K): δ 8.65 (ddd, *J* = 4.9, 1.9, 0.9, 1H), 8.11 (d, *J* = 8.5, 1H), 7.81 (d, *J* = 8.4, 1H), 7.71–7.63 (m, 4H), 7.57–7.49 (m, 5H), 7.42–7.36 (m, 5H), 7.32–7.27 (m, 1H), 7.19 (ddd, *J* = 7.5, 4.9, 1.1, 1H), 6.12 (s, 1H). ^13^C NMR (75 MHz, CDCl_3_, 298 K): δ 162.8, 160.2, 154.7 (all C_q_), 149.3 (CH), 142.3, 139.8, 137.6 (all C_q_), 136.5, 130.3 (2C), 129.9, 129.6 (2C), 128.5 (3C), 128.3 (2C), 127.4, 126.9, 126.7 (all CH), 125.4 (C_q_), 124.5, 121.6, 119.6, 61.6 (all CH).

### Synthesis of 1-Phenyl-3-(1-phenyl-1-(pyridine-2-yl)ethyl)isoquinoline (H_2_MeL)

Lithium chloride (68 mg, 1.62 mmol) was added to a solution of H_2_L (300 mg, 0.81 mmol) in 5 mL of THF, and the reaction mixture was cooled to −78 °C. Lithium diisopropylamide (2 M, 3.24 mL, 1.62 mmol) was added dropwise over 10 min; then, the reaction mixture was kept at −78 °C for 1 h. Methyl iodide (100 μL, 1.62 mmol) was added dropwise over 5 min. The mixture was stirred at −78 °C for 30 min, warmed to room temperature, quenched with saturated aqueous NH_4_Cl, and extracted with EtOAc. The organic fractions were dried with MgSO_4_ and concentrated. The crude was purified by column chromatography (silica gel) using 0–30% EtOAc/*n*-hexane to give a white solid (217.9 mg, 70%). HRMS (electrospray, *m*/*z*): calcd for C_28_H_23_N_2_ [M + H]^+^, 387.1856; found, 387.1840. ^1^H NMR (300 MHz, CD_2_Cl_2_): δ 8.61 (m, 1H), 8.14 (m, 1H), 7.77 (m, 1H), 7.71–7.67 (m, 2H), 7.65–7.62 (m, 1H), 7.60–7.57 (m, 1H), 7.56–7.49 (m, 4H), 7.40 (s, 1H), 7.37–7.22 (m, 6H), 7.17–7.14 (m, 1H), 2.43 (s, 3H). ^13^C NMR (75 MHz, CD_2_Cl_2_): δ 167.5, 159.7, 159.6 (all C_q_), 149.2 (CH), 148.8, 140.4, 137.8 (all C_q_), 136.2, 130.7 (2C), 130.3, 129.4 (2C), 129.0, 128.7 (2C), 128.5 (2C), 127.9, 127.6, 127.5, 126.6 (all CH), 125.4 (C_q_), 124.5, 121.6, 119.2 (all CH), 58.1 (C_q_), 28.6 (CH_3_).

### Preparation of IrCl(κ^4^-*cis*-*C*,*C*′-*cis*-*N*,*N*′-MeL)(CO) (**3**)

Complex **1** (250 mg, 0.372 mmol) and H_2_MeL (and 287.7 mg, 0.744 mmol) in 5 mL of 2-ethoxyethanol were heated under reflux. After 48 h, an orange solid precipitated, which was separated by decantation, washed with methanol (3 × 5 mL), and dried under vacuum. Yield: 314 mg (66%). Crystals of **3** suitable for X-ray diffraction analysis were formed by diffusion of pentane into a dichloromethane solution of the precipitate at 4 °C. Anal. Calcd for C_29_H_20_IrClN_2_O: C, 54.41; H, 3.15; N, 4.38. Found: C, 54.73; H, 3.19; N, 4.12. HRMS (electrospray, *m*/*z*): calcd for C_30_H_23_IrN_3_ [M–Cl–CO + CH_3_CN]^+^, 618.1517; found, 618.1513. *T*_d5_ = 385 °C.^35^ IR (cm^–1^): ν (CO) 2023 (s). ^1^H NMR (300 MHz, CD_2_Cl_2_, 298 K): δ 9.31 (d, *J* = 8.1, 1H), 8.89 (d, *J* = 7.8, 1H), 8.20 (d, *J* = 7.9, 1H), 8.16 (dd, *J* = 7.6, 1.2, 1H), 8.03–7.90 (m, 3H), 7.81 (s, 1H), 7.81–7.69 (m, 3H), 7.42 (dd, *J* = 8.0, 1.4, 1H), 7.33–7.24 (m, 2H), 7.17 (m, 1H), 6.98 (m, 1H), 6.84 (m, 1H), 2.72 (s, 3H, *Me*L).^13^C{^1^H} NMR (101 MHz, CD_2_Cl_2_): δ 172.6 (CO), 169.4, 159.4 (both C_q_), 157.1 (CH), 152.5, 147.8, 147.1 (all C_q_), 140.4 (CH), 139.5 (C_q_), 138.9 (CH), 138.8 (C_q_), 137.7 (CH), 133.3 (C_q_), 132.7, 131.1, 130.7, 129.2, 128.5, 127.4, 126.7 (all CH), 125.6 (C_q_), 125.0, 124.8, 124.7, 123.7, 121.9, 115.6 (all CH), 59.0 (C_q_), 23.1 (*Me*L).

### Preparation of [Ir(μ-Cl)(κ4-*cis*-*C*,*C*′-*cis*-*N*,*N*′-MeL)]_2_ (**4**)

Complex **1** (375 mg, 0.558 mmol) or **2** (500 mg, 0.558 mmol) and H_2_MeL (430 mg, 1.11 mmol) in 7 mL of 1-phenylethanol were stirred at 140 °C. After 72 h, a brown solid was formed, separated by decantation, and washed with diethyl ether until mother liquors were colorless. Yield: 230 mg (34%) starting from **1**, 557 mg (82%) starting from **2**. Anal. Calcd for C_56_H_40_Cl_2_Ir_2_N_4_: C, 54.94; H, 3.29; N, 4.58. Found: C, 55.03; H, 3.10; N, 4.38. MS (MALDI-TOF, *m*/*z*): calcd for C_28_H_20_ClIrN_2_ [M/2]^+^, 612.1; found, 612.2.

### Preparation of Ir(κ^4^-*cis*-*C*,*C*′-*cis*-*N*,*N*′-MeL)(acac) (**5**)

Acetylacetone (350 μL, 3.41 mmol) and KOH (225 mg, 3.41 mmol) in 4 mL of methanol were poured into a suspension of **4** (557 mg, 0.45 mmol) in 15 mL of THF. The mixture was stirred at 60 °C for 6 h. The solvent was removed under vacuum to afford an orange residue, which was treated with 15 mL of dichloromethane. The suspension formed was filtered over Celite, and the resulting solution was concentrated under vacuum. The addition of 5 mL of pentane yielded a reddish brown solid, which was purified by column chromatography (silica gel) using dichloromethane as the eluent. Yield: 492 mg (80%). X-ray quality crystals were obtained by evaporation in dichloromethane at 4 °C. Anal. Calcd for C_33_H_27_IrN_2_O_2_: C, 58.65; H, 4.03; N, 4.15. Found: C, 58.31; H, 3.99; N, 4.25. HRMS (electrospray, *m*/*z*): calcd for C_33_H_27_IrN_2_O_2_ [M + Na]^+^, 699.1596; found, 699.1601. *T*_d5_ = 360 °C.^36^ IR (cm^–1^): ν(C=O) 1574 (s), 1508 (s). ^1^H NMR (400 MHz, CD_2_Cl_2_, 298 K): δ 8.81 (m, 1H), 8.25 (m, 1H), 8.18 (d, *J* = 7.8, 1H), 7.89–7.80 (m, 3H), 7.78 (dd, *J* = 7.4, 1.5, 1H), 7.65 (s, 1H), 7.62 (m, 2H), 7.55 (dd, *J* = 6.7, 2.3, 1H), 7.24 (m, 2H), 7.15 (ddd, *J* = 7.3, 7.3, 1.3, 1H), 7.08 (ddd, *J* = 7.8, 1.6, 1H), 6.85–6.76 (m, 2H), 5.48 (s, 1H, CH acac), 2.68 (*Me*L), 2.20, 1.58 (both s, 3H each, CH_3_ acac). ^13^C{^1^H} NMR (101 MHz, CD_2_Cl_2_): δ 185.3, 184.9 (both CO acac), 171.7, 163.7, 161.5, 152.0 (all C_q_), 151.5 (CH), 149.1, 140.6 (both C_q_), 138.0 (CH), 137.4, 137.0 (both C_q_), 136.2, 134.1, 130.7, 129.5, 128.5, 128.2, 127.9, 125.6, 125.3 (all CH), 125.2 (C_q_), 124.4, 122.9, 122.8, 122.1, 121.3, 114.4 (all CH), 101.4 (CH acac), 58.6 (C_q_), 28.6, 28.3 (both CH_3_ acac), 23.3 (*Me*L).

### Preparation of *fac*-Ir(κ^4^-*cis*-*C*,*C*′-*cis*-*N*,*N*′-MeL)(κ^2^-*C*,*N*-C_6_H_4_-py) (**6a**)

A solution of 2-(2-bromophenyl)pyridine (81.6 μL, 0.488 mmol) in 5 mL of THF was cooled to −78 °C, and *n*-BuLi (321 μL, 1.6 M in hexanes, 0.512 mmol) was added dropwise. After stirring at the same temperature for 1 h, a precooled (−78 °C) suspension of **4** (0.122 mmol) in 5 mL of THF was cannula-transferred into the lithiation flask, and the mixture was allowed to slowly warm to room temperature over 18 h. The solvent was then evaporated. The residue was extracted with dichloromethane (3 × 10 mL) and purified by flash column chromatography using a 3:1 mixture of dichloromethane/pentane, affording compound **6a** as a dark-red solid. Yield: 35 mg (20%). X-ray quality crystals of **6a** were obtained in dichloromethane by evaporation at room temperature. Anal. Calcd for C_39_H_28_IrN_3_: C, 64.09; H, 3.86; N, 5.75. Found: C, 64.17; H, 4.02; N, 5.98. HRMS (electrospray, *m*/*z*): calcd for C_39_H_29_IrN_3_ [M + H]^+^, 732.1963; found, 732.1924. *T*_d5_ = 305 °C.^36^^1^H NMR (400 MHz, CD_2_Cl_2_, 298 K): δ 8.86 (m, 1H), 8.15 (d, *J* = 7.3, 1H), 8.09 (d, *J* = 7.5, 1H), 7.99 (m, 1H), 7.96–7.87 (m, 5H), 7.72–7.63 (m, 3H), 7.56–7.50 (m, 2H), 7.37 (m, 1H), 7.24–7.16 (m, 3H), 7.09 (d, *J* = 7.5, 1H), 6.87–6.80 (m, 3H), 6.75 (t, *J* = 7.3, 1H), 6.63–6.58 (m, 2H), 2.76 (s, 3H, *Me*L). ^13^C NMR (75 MHz, CD_2_Cl_2_): δ 168.8, 167.7, 167.1, 164.7, 161.6 (all C_q_), 151.2 (CH), 150.8, 150.0, 147.8 (all C_q_), 147.7 (CH), 146.1, 140.7 (both C_q_), 139.1 (CH), 137.8 (C_q_), 137.7, 137.0, 136.9, 136.7, 130.7, 130.1, 130.1, 129.1, 128.5, 127.9, 126.0, 125.9, 125.3, 123.3, 122.8 (all CH), 122.7 (C_q_), 122.0, 121.6, 121.4, 120.8, 119.2, 114.6 (all CH), 59.6 (C_q_) 24.1 (*Me*L).

### Preparation of [Ir(κ^4^-*cis*-*C*,*C*′-*cis*-*N*,*N*′-MeL)(CH_3_CN)_2_]BF_4_ (**7**)

Silver tetrafluoroborate (95.4 mg, 0.490 mmol) dissolved in acetone (5 mL) was added to **4** (300 mg, 0.245 mmol) in dichloromethane (15 mL). The mixture protected from light was stirred for 5 h and filtered through Celite to remove the formed silver chloride. The solution was concentrated under vacuum. The addition of 3 mL of diethyl ether afforded an orange solid, which was dissolved in acetonitrile (5 mL) and filtered off. The resulting solution was concentrated until about 0.5 mL. The subsequent addition of 3 mL of diethyl ether gave an orange solid. Yield: 319 mg (87%). Anal. Calcd for C_32_H_26_BF_4_IrN_4_: C, 51.55; H, 3.51; N, 7.51. Found: C, 51.23; H, 3.71; N, 7.80. HRMS (electrospray, *m*/*z*): calcd for C_30_H_23_IrN_3_ [M–BF_4_–CH_3_CN]^+^, 618.1517; found, 618.1478. ^1^H NMR (300 MHz, CD_2_Cl_2_, 298 K): δ 9.23 (d, *J* = 7.9, 1H), 8.83 (m, 1H), 8.16 (d, *J* = 8.1, 1H), 8.03–7.91 (m, 4H), 7.83 (s, 1H), 7.80–7.72 (m, 2H), 7.55–7.50 (m, 2H), 7.31 (d, *J* = 9.2, 1H), 7.23 (m, 1H), 7.08 (m, 1H), 6.93–6.76 (m, 2H), 2.81 (s, 3H, CH_3_CN), 2.71 (s, 3H, *Me*L), 2.11 (s, 3H, CH_3_CN). ^13^C{^1^H} NMR (101 MHz, CD_2_Cl_2_): δ 171.3 (C_q_), 159.7 (C_q_), 153.9 (CH), 150.0, 148.6, 140.5 (all C_q_), 139.9 (CH), 138.0 (C_q_), 134.7, 134.2 (both CH), 134.0 (C_q_), 132.4 (CH), 130.4 (2 CH), 129.0, 128.5, 127.0 (all CH), 125.7 (2 CH), 125.0 (C_q_), 124.5, 124.1, 123.7, 122.0 (all CH), 118.4, 118.1 (both CH_3_*C*N) 115.9 (CH), 58.8 (C_q_), 23.1 (*Me*L), 4.7, 3.5 (both *C*H_3_CN). One of the C_q_ signals of the tetradentate ligand is not observed because it is overlapped with other signals.

### Reaction of 7 with 2-Phenylpyridine: Formation of *mer*-Ir(κ^4^-*cis*-*C*,*C*′-*cis*-*N*,*N*′-MeL){κ^2^-*C*,*N*-(C_6_H_4_-py)} (6b) and [Ir(κ^3^-*C*,*N*,*N*′;η^2^-*C*,*C*)-MeHL)(κ^2^-*C*,*N*-C_6_H_4_-py)]BF_4_ (**8**)

Complex **7** (300 mg, 0.402 mmol), 2-phenylpyridine (58.6 μL, 0.402 mmol), and (piperidinomethyl)polystyrene (115 mg, 0.402 mmol) were stirred in 10 mL of fluorobenzene under reflux. After 48 h, the mixture was cooled to room temperature and filtered through Celite, and the resulting solution was concentrated. The residue was extracted with dichloromethane. Addition of pentane (5 mL) led to a mixture of **6b** and **8**, which were separated by column chromatography (neutral aluminum oxide) using dichloromethane as the eluent to afford **6b** [dark-red solid, yield: 32 mg (11%)] and then acetonitrile to obtain **8** [orange solid, yield: 51.2 mg (32%)]. X-ray quality crystals of **6b** were formed in dichloromethane by evaporation at room temperature. Analytical and spectroscopic data of **6b**: Anal. Calcd for C_39_H_28_IrN_3_: C, 64.09; H, 3.86; N, 5.75. Found: C, 64.21; H, 4.03; N, 5.71. HRMS (electrospray, *m*/*z*): calcd for C_39_H_29_IrN_3_ [M + H]^+^, 732.1987; found, 732.1960. *T*_d5_ = 336 °C.^36^^1^H NMR (400 MHz, CD_2_Cl_2_, 298 K): δ 9.43 (d, *J* = 5.6, 1H), 8.85 (d, *J* = 7.8, 1H), 8.11 (m, 2H), 7.97–7.86 (m, 4H), 7.83 (s, 1H), 7.77 (d, *J* = 7.7, 1H), 7.72–7.62 (m, 3H), 7.53 (m, 1H), 7.31 (dd, *J* = 5.5, 1.1, 1H), 7.18 (ddd, *J* = 7.3, 5.9, 1.5, 1H), 6.95–6.77 (m, 5H), 6.65 (m, 1H), 6.61 (m, 1H), 6.22 (d, *J* = 7.2, 1H), 2.80 (s, 3H, *Me*L). ^13^C NMR (101 MHz, CD_2_Cl_2_): δ 178.9, 171.1, 169.0, 168.3, 165.2, 161.9 (all C_q_), 153.1 (CH), 150.9 (C_q_), 150.2 (CH), 146.5, 145.0, 141.5 (all C_q_), 138.3, 137.1, 137.0 (all CH), 136.5 (C_q_), 133.3, 132.0, 130.3, 130.2, 129.3, 129.1, 128.2, 127.8, 125.9, 125.7 (all CH), 125.4 (C_q_), 124.7, 123.2, 123.0, 122.8, 122.7, 121.8 (2C), 120.4, 119.7, 114.4 (all CH), 60.7 (C_q_), 24.6 (*Me*L). For analytical and spectroscopic data of **8**, see below.

### Preparation of [Ir(κ^3^-*C*,*N*,*N*′;η^2^-*C*,*C*)-MeHL)(κ^2^-*C*,*N*-C_6_H_4_-py)]BF_4_ (8)

A mixture of **7** (200 mg, 0.268 mmol) and 2-phenylpyridine (42.5 μL, 0.268 mmol) was stirred in 10 mL of 2-propanol under reflux. After 48 h, an orange solid appeared, which was separated by decantation, and it was washed with diethyl ether (3 × 5 mL). Yield: 134 mg (61%). X-ray quality crystals were obtained from dichloromethane–diethyl ether by diffusion at 4 °C. Anal. Calcd for C_39_H_29_BF_4_IrN_3_: C, 57.22; H, 3.57; N, 5.13. Found: C, 56.92; H, 3.45; N, 5.08. HRMS (electrospray, *m*/*z*): calcd for C_39_H_29_IrN_3_ [M-BF_4_]^+^, 732.1987; found, 732.1973. IR (cm^–1^): ν (BF_4_) 1049 (s). ^1^H NMR (300 MHz, CD_2_Cl_2_, 298 K): δ 9.02 (d, *J* = 5.6, 1H), 8.93 (d, *J* = 8.2, 1H), 8.81 (d, *J* = 5.1, 1H), 8.20 (m, 2H), 8.14 (m, 1H), 8.06 (s, 1H), 8.03–7.83 (m, 5H), 7.58 (m, 1H), 7.52 (m, 1H), 7.34 (d, *J* = 7.9, 1H), 7.15 (d, *J* = 8.0, 1H), 7.00 (dd, *J* = 7.2, 7.2, 1H), 6.86–6.73 (m, 2H), 6.68–6.54 (m, 3H), 6.42 (d, *J* = 7.7, 1H), 6.28 (m, 2H), 5.97 (d, *J* = 7.3, 1H), 2.34 (s, 3H, *Me*L). ^19^F NMR (282.38 MHz, CD_2_Cl_2_, 298 K): δ −153.17 (s). ^13^C NMR (100 MHz, CD_2_Cl_2_): δ 167.6, 167.0, 161.7, 156.2 (all C_q_), 150.5, 149.4 (both CH), 148.3, 146.8, 145.1, 142.6 (all C_q_), 141.1, 139.3 (both CH), 137.8 (C_q_), 133.3, 132.9, 132.4, 131.4, 131.1 (3C), 131.0, 129.7, 129.7 (all CH), 129.0 (C_q_), 128.9 (2C), 126.1, 125.9, 125.6 (all CH), 125.0 (C_q_), 124.0 (2C), 123.2, 123.1, 120.8, 117.4, 117.0 (all CH), 60.2 (C_q_), 25.3 (*Me*L).

### Preparation of *mer*-Ir(κ^4^-*trans*-*C*,*C*′-*cis*-*N*,*N*′-MeL){κ^2^-*C*,*N*-(C_6_H_4_-py)} (**6c**)

KO^*t*^Bu (41.5 mg, 0.368 mmol) dissolved in 5 mL of THF was slowly added (5–10 min) to **8** (76 mg, 0.093 mmol) in 5 mL of THF. The initial orange/red suspension became a reddish-brown solution. After 5 h, the solvent was removed under vacuum and the product was extracted with dichloromethane (3 × 10 mL). The dichloromethane solution was concentrated under vacuum. The addition of pentane yielded a brown solid that was purified by column chromatography (basic alumina) using dichloromethane as the eluent. A reddish-brown solid was obtained. Yield: 51 mg (75%). Anal. Calcd for C_39_H_28_IrN_3_: C, 64.09; H, 3.86; N, 5.75. Found: C, 63.86; H, 3.99; N, 5.54. HRMS (electrospray, *m*/*z*): calcd for C_39_H_29_IrN_3_ [M + H]^+^, 732.1987; found, 732.1946. *T*_d5_ = 322 °C.^36^^1^H NMR (300 MHz, CD_2_Cl_2_, 298 K): δ 9.16 (d, *J* = 5.4, 1H), 8.85 (d, *J* = 9.0, 1H), 8.61 (d, *J* = 7.2, 1H), 8.15 (dd, *J* = 7.0, 1.5, 1H), 8.08 (d, *J* = 8.0, 1H), 7.93 (dd, *J* = 7.6, 1.6, 1H), 7.88–7.63 (m, 7H), 7.61 (d, *J* = 7.8, 1H), 7.17–7.09 (m, 2H), 7.06 (ddd, *J* = 7.2, 5.8, 1.5, 1H), 6.94–6.85 (m, 2H), 6.84–6.76 (m, 2H), 6.53 (dd, *J* = 8.1, 8.1, 1H), 6.46 (dd, *J* = 7.1, 7.1, 1H), 6.08–6.03 (m, 2H), 2.80 (s, 3H, *Me*L). ^13^C NMR (75 MHz, CD_2_Cl_2_): δ 182.0, 173.6, 169.1, 163.7, 161.5, 161.3 (all C_q_), 153.3 (CH), 152.7, 150.5 (both C_q_), 148.7 (CH), 143.3, 142.1 (both C_q_), 138.3 (CH), 137.7 (C_q_), 135.6, 135.4, 134.0, 131.4, 131.2, 130.8, 129.1, 128.9, 128.0, 127.9, 126.2 (all CH), 125.6 (C_q_), 125.3, 124.3, 123.9, 122.6, 122.4, 122.3, 121.8, 121.2, 120.1, 120.0, 114.3 (all CH), 61.9 (C_q_), 24.4 (*Me*L).

### Formation of *mer*-Ir(κ^4^-*cis*-*C*,*C*′-*cis*-*N*,*N*′ -MeL){κ^2^-*C*,*N*-(C_6_H_3_Me-py)} (**9b**) and [Ir(κ^3^-*C*,*N*,*N*′;η^2^-*C*,*C*)-MeHL)(κ^2^-*C*,*N*-C_6_H_3_Me-py)]BF_4_ (**10**)

These compounds were obtained following the procedure described for **6b** and **8** starting from **7** (275 mg, 0.368 mmol), 2-(*p*-tolyl)pyridine (63 μL, 0.368 mmol), and (piperidinomethyl)polystyrene (105 mg, 0.368 mmol). Yield: **9b** (red solid), 34 mg (12%); **10** (orange solid), 120 mg (39%). X-ray quality crystals of **9b** were formed from dichloromethane by evaporation at room temperature. Analytical and spectroscopic data of **9b**: Anal. Calcd for C_40_H_30_IrN_3_: C, 64.50; H, 4.06; N, 5.64. Found: C, 64.35; H, 3.99; N, 5.42. HRMS (electrospray, *m*/*z*): calcd for C_40_H_30_IrN_3_ [M + H]^+^, 746.2144; found, 746.2168. *T*_d5_ = 350 °C.^36^^1^H NMR (300 MHz, CD_2_Cl_2_, 298 K): δ 9.38 (d, *J* = 5.8, 1H), 8.84 (dd, *J* = 7.5, 1.5, 1H), 8.08 (m, 2H), 7.98–7.84 (m, 4H), 7.83 (s, 1H), 7.73–7.60 (m, 4H), 7.53 (m, 1H), 7.32 (dd, *J* = 5.6, 1.4, 1H), 7.13 (ddd, *J* = 7.3, 5.8, 1.4, 1H), 6.94–6.88 (m, 2H), 6.88–6.75 (m, 3H), 6.70 (dd, *J* = 7.9, 1.2, 1H), 6.65 (ddd, *J* = 7.1, 5.6, 1.1, 1H), 6.04 (s, 1H), 2.80 (s, *Me*L), 1.85 (s, 3H, C_6_H_3_*Me*). ^13^C NMR (75 MHz, CD_2_Cl_2_): δ 178.9, 171.1, 169.1, 168.4, 165.3, 161.8 (all C_q_), 152.9 (CH), 150.7 (C_q_), 150.2 (CH), 146.6, 142.3, 141.4, 139.2 (all C_q_), 138.3, 137.0 (2C) (all CH), 136.4 (C_q_), 134.1, 132.0, 130.2 (2C), 129.0, 128.1, 127.7, 125.9, 125.5 (all CH), 125.4 (C_q_), 124.6, 123.1, 123.0, 122.9, 122.7, 122.2, 121.88 (2C), 120.0, 119.5, 114.3 (all CH), 60.6 (C_q_), 24.6 (*Me*L), 22.0 (C_6_H_3_*Me*). For analytical and spectroscopic data of **10**, see below.

### Preparation of [Ir(κ^3^-*C*,*N*,*N*′;η^2^-*C*,*C*)-MeHL)(κ^2^-*C*,*N*-C_6_H_3_Me-py)]BF_4_ (10)

This compound was prepared as **8** starting from **7** (95 mg, 0.127 mmol) and 2-(*p*-tolyl)pyridine (21.8 μL, 0.127 mmol). Orange solid. Yield: 58 mg (55%). Anal. Calcd for C_40_H_31_BF_4_IrN_3_: C, 57.69; H, 3.75; N, 5.05. Found: C, 57.38; H, 3.64; N, 5.15. HRMS (electrospray, *m*/*z*): calcd for C_40_H_31_IrN_3_ [M-BF_4_]^+^ 746.2144; found, 746.2134. IR (cm^–1^): ν (BF_4_) 1049 (s). ^1^H NMR (400 MHz, CD_2_Cl_2_, 298 K): δ 8.96–8.91 (m, 2H), 8.78 (m, 1H), 8.23–8.17 (m, 2H), 8.14 (ddd, *J* = 7.9, 7.9, 1.6, 1H), 8.04 (s, 1H), 8.02–7.87 (m, 4H), 7.81 (m, 1H), 7.56 (m, 1H), 7.45 (ddd, *J* = 7.3, 5.9, 1.5, 1H), 7.23 (d, *J* = 7.9, 1H), 7.17 (m, 1H), 7.00 (ddd, *J* = 8.6, 7.3, 1.2, 1H), 6.83 (m, 1H), 6.69–6.53 (m, 3H), 6.41 (dd, *J* = 7.9, 1.1, 1H), 6.35–6.25 (m, 2H), 5.75 (s, 1H), 2.34 (s, 3H, *Me*L), 1.96 (s, 3H, C_6_H_3_*Me*). ^19^F NMR (282.38 MHz, CD_2_Cl_2_, 298 K): δ −152.64 (s). ^13^C NMR (75 MHz, CD_2_Cl_2_): δ 167.8, 167.3, 161.9, 156.4 (all C_q_), 150.6, 149.3 (both CH), 148.5, 147.1, 145.5 (all C_q_), 141.2 (CH), 140.2, 140.1 (both C_q_), 139.3 (CH), 137.9 (C_q_), 134.1, 133.0, 132.4, 131.5, 131.2 (2C), 131.0, 130.7, 129.9 (all CH), 129.2 (C_q_), 129.1, 129.0, 126.2, 125.9, 125.7 (all CH), 125.2 (C_q_), 124.4, 124.1, 123.6, 123.2, 120.6, 118.0, 116.9 (all CH), 60.4 (C_q_), 25.4 (*Me*L), 21.8 (C_6_H_3_*Me*).

### Preparation of *mer*-Ir(κ^4^-*trans*-*C*,*C*′-*cis*-*N*,*N*′-MeL){κ^2^-*C*,*N*-(C_6_H_3_Me-py)} (**9c**)

This compound was obtained by a similar procedure to that described for **6c**, starting from **10** (120 mg, 0.144 mmol) and KOtBu (64.8 mg, 0.576 mmol). Dark-red solid, yield: 85 mg (80%). Anal. Calcd for C_40_H_30_IrN_3_: C, 64.50; H, 4.06; N, 5.64. Found: C, 64.23; H, 4.10; N, 5.53. HRMS (electrospray, *m*/*z*): calcd for C_40_H_30_IrN_3_ [M + H]^+^ 746.2139; found, 746.2115. *T*_d5_ = 334 °C.^36^^1^H NMR (300 MHz, CD_2_Cl_2_, 298 K): δ 9.16 (dd, *J* = 5.3, 1.5, 1H), 8.84 (d, *J* = 7.8, 1H), 8.58 (d, *J* = 5.7, 1H), 8.14 (dd, *J* = 7.2, 1.7, 1H), 8.03 (d, *J* = 8.0, 1H), 7.94 (dd, *J* = 7.6,1.7, 1H), 7.85 (d, *J* = 8.0, 1H), 7.83 (s, 1H), 7.81–7.65 (m, 4H), 7.61 (d, *J* = 7.9, 2H), 7.20–7.09 (m, 2H), 7.02 (ddd, *J* = 7.3, 5.9, 1.4, 1H), 6.96–6.78 (m, 2H), 6.81 (ddd, *J* = 7.5, 7.2, 1.5, 1H), 6.64 (dd, *J* = 7.9, 1.1, 1H), 6.47 (ddd, *J* = 7.1, 7.1, 1.0, 1H), 6.07 (dd, *J* = 7.2, 1.5, 1H), 5.90 (s, 1H), 2.80 (s, 3H, *Me*L), 1.85 (s, 3H, C_6_H_3_*Me*). ^13^C NMR (75 MHz, CD_2_Cl_2_): δ 182.2, 173.6, 169.0, 163.8, 161.4, 161.3 (all C_q_), 153.3 (CH), 152.6, 150.7 (both C_q_), 148.6 (CH), 142.2, 140.6, 139.1 (all C_q_), 138.2 (CH), 137.7 (C_q_), 135.7, 135.3, 134.1, 132.0, 131.1, 130.8, 128.8, 128.0, 127.8, 126.0 (all CH), 125.7 (C_q_), 125.3, 124.1, 123.8, 122.6, 122.3 (2C), 121.3, 121.2 (2C), 119.6, 114.2 (allCH), 61.8 (C_q_), 24.4 (*Me*L), 22.0 (C_6_H_3_*Me*).

## Supporting Information Available

General information for the experimental section, structural analysis, computational details and energies of optimized structures, experimental and computed UV/vis spectra, cyclic voltammograms, frontier orbitals, natural transition orbitals, normalized excitation and emission spectra, NMR spectra, and TGA curves (PDF)
Cartesian coordinates of the optimized structures (XYZ)

## Supplementary Material

ic1c01303_si_001.pdf

ic1c01303_si_002.xyz
