# Supplementary material for: Pseudo-Tris(heteroleptic) Red Phosphorescent Iridium(III) Complexes Bearing a Dianionic C,N,C′,N′-Tetradentate Ligand
Source: Inorg Chem. 2021 Jul 22;60(15):11347–63. doi: 10.1021/acs.inorgchem.1c01303 (PMC9179949; doi:10.1021/acs.inorgchem.1c01303)
Supplement: Supplementary file 1 — ic1c01303_si_001.pdf [file ic1c01303_si_001.pdf]

Supporting Information for

Pseudo-Tris(heteroleptic) Red Phosphorescent Iridium(III) Complexes Bearing a Dianionic C,N,C′,N′-Tetradentate Ligand

Vadim Adamovich,[b] Llorenç Benavent,[a] Pierre-Luc T. Boudreault,[b] Miguel A. Esteruelas,[,a] Ana M. López,[a] Enrique Oñate,[a] and Jui-Yi Tsai[b]*

[a] Departamento de Química Inorgánica, Instituto de Síntesis Química y Catálisis Homogénea (ISQCH), Centro de Innovación en Química Avanzada (ORFEO-CINQA), Universidad de Zaragoza-CSIC, 50009 Zaragoza, Spain.

[b] Universal Display Corporation, Ewing, New Jersey 08618, United States.

*Corresponding author's e-mail address: maester@unizar.es

CONTENTS

Experimental Section: General Information S3

Structural Analysis of Complexes 3, 5, 6a, 6b, 8, 9b and 9c S3

Computational Details S6

Energies of Optimized Structures S6

UV-vis Spectra (Observed and Calculated) S11

Analysis of Computed UV/Vis Data S15

Theoretical Analysis of Molecular Orbitals S25

Cyclic Voltammograms S45

Normalized Excitation and Emission Spectra S46

NMR Spectra S56

TGA curves S80

References S81

Experimental Section: General information. All reactions were performed with rigorous exclusion of air at an argon/vacuum manifold using standard Schlenk-tube or glovebox techniques. Solvents were dried by the usual procedures and distilled under argon prior to use or obtained oxygen- and water-free from an MBraun solvent purification apparatus. NMR spectra were recorded on a Bruker ARX 300, Bruker Avance 300 MHz, or a Bruker Avance 400 MHz instruments. Chemical shifts (expressed in parts per million) are referenced to residual solvent peaks (^{1}H, $^{13}C\{^{1}H\}$). Coupling constants *J* are given in Hertz. C, H, and N analyses were carried out in a Perkin-Elmer 2400-B Series II CHNS-Analyzer. Thermal gravimetric analysis (TGA) were run with a thermobalance from TA Instruments (model SDT 2960) under N_2 at a heating rate of 10 ºC/min. High-resolution electrospray (HRMS) and MALDI-TOF mass spectra were acquired using a MicroTOF-Q hybrid quadrupole time-of-flight spectrometer and a Bruker Autoflex III, MALDITOF/TOF equipped with a DCTB matrix, respectively (Bruker Daltonics, Bremen, Germany). Attenuated total reflection infrared spectra (ATR-IR) of solid samples were run on a Perkin-Elmer Spectrum 100 FT-IR spectrometer. UV-visible spectra were registered on an Evolution 600 spectrophotomer. Steady-state photoluminescence spectra were recorded on a Jobin-Yvon Horiba Fluorolog FL-3-11 spectrofluorimeter. Lifetimes were measured using an IBH 5000F coaxial nanosecond flash lamp. Quantum yields were measured using the Hamamatsu Absolute PL Quantum Yield Measurement System C11347-11. Cyclic voltammetry measurements were performed using a Voltalab PST050 potentiostat with Pt wire as working electrode, Pt wire as counter electrode, and saturated calomel (SCE) as reference electrode. The experiments were carried out under argon in acetonitrile solutions (10^{-3} M), with Bu_4NPF_6 as supporting electrolyte (0.1 M). Scan rate was 100 $mV \cdot s^{-1}$. The potentials were referenced to the ferrocene/ferrocenium (Fc/Fc^{+}) couple. The emitters have been tested in bottom emission OLED structures with the device pixel area 2 mm^2. The glass coated ITO substrates were patterned by photolithography. The device pixel area was defined by the photoresist grid. The devices were fabricated by high vacuum ($<10^{-7}$ Torr) thermal evaporation (VTE). Organic layers have been deposited with the deposition rate 2Å/s.

Structural Analysis of Complexes 3, 5, 6a, 6b, 8, 9b and 9c. X-ray data were collected for the complexes on a Bruker Smart APEX and Bruker APEX DUO diffractometers equipped with a normal or fine focus, and 2.4 kW sealed tube source (Mo radiation, $\lambda = 0.71073$ Å). Data were collected over the complete sphere covering 0.3° in ω. Data were corrected for absorption by using a multiscan method applied with the SADABS program.[1] The structures were solved by Patterson or direct methods and refined by full-matrix least squares on F^2 with

SHELXL2016,[2] including isotropic and subsequently anisotropic displacement parameters. The hydrogen atoms were observed in the last Fourier Maps or calculated, and refined freely or using a restricted riding model. The disordered solvent molecules of crystallization were refined with restrained geometry, complementary occupancy factors and isotropic displacement factors.

Crystal data for **3** (CCDC 2077159): $C_{29}H_{20}Cl_1IrN_2O$, CH_2Cl_2, Mw 725.04, yellow, irregular block (0.300 x 0.086 x 0.073 mm^3), triclinic, space group P-1, *a*: 9.1869(8) Å, *b*: 11.3570(10) Å, *c*: 13.2440(12) Å, *α*: 92.5520(10)°, *β*: 104.7660(10)°, *γ*: 100.2950(10)°, *V* = 1308.8(2) Å^3, *Z* = 2, *Z'* = 1, D_{calc}: 1.840 g cm^{-3}, F(000): 704, *T* = 120(2) K, *μ* 5.435 mm^{-1}. 20428 measured reflections (2θ: 3-57°, ω scans 0.3°), 6829 unique (R_{int} = 0.0270); min./max. transm. Factors 0.600/0.862. Final agreement factors were R^1 = 0.0277 (6201 observed reflections, I > 2*σ*(I)) and wR^2 = 0.0694; data/restraints/parameters 6829/6/329; GoF = 1.033. Largest peak and hole 2.420 (close to iridium atoms) and -2.342e/ Å^3.

Crystal data for **5** (CCDC 2077165): $C_{33}H_{27}IrN_2O_2$, CH_2Cl_2, Mw 760.69, red, irregular block (0.156 x 0.116 x 0.115 mm^3), monoclinic, space group P2$_1$/n, *a*: 12.1867(6) Å, *b*: 16.0864(8) Å, *c*: 14.8000(8) Å, *β*: 101.8510(10)°, *V* = 2839.6(3) Å^3, *Z* = 4, *Z'* = 1, D_{calc}: 1.779 g cm^{-3}, F(000): 1496, *T* = 100(2) K, *μ* 4.926 mm^{-1}. 49593 measured reflections (2θ: 3-57°, ω scans 0.3°), 7014 unique (R_{int} = 0.0418); min./max. transm. Factors 0.718/0.862. Final agreement factors were R^1 = 0.0269 (5971 observed reflections, I > 2*σ*(I)) and wR^2 = 0.0670; data/restraints/parameters 7014/0/376; GoF = 1.018. Largest peak and hole 1.632 (close to iridium atoms) and -1.707 e/ Å^3.

Crystal data for **6a** (CCDC 2077163): $C_{39}H_{28}IrN_3$, 0.5(CH_2Cl_2), Mw 773.31, red, irregular block (0.241 x 0.125 x 0.076 mm^3), monoclinic, space group P2$_1$/n, *a*: 10.6373(12) Å, *b*: 19.680(2) Å, *c*: 14.4133(17) Å, *β*: 96.734(2)°, *V* = 2996.4(6) Å^3, *Z* = 4, *Z'* = 1, D_{calc}: 1.714 g cm^{-3}, F(000): 1524, *T* = 120(2) K, *μ* 4.580 mm^{-1}. 71828 measured reflections (2θ: 3-57°, ω scans 0.3°), 8343 unique (R_{int} = 0.0350); min./max. transm. Factors 0.676/0.862. Final agreement factors were R^1 = 0.0251 (7216 observed reflections, I >2*σ*(I)) and wR^2 = 0.0611; data/restraints/parameters 8343/2/400; GoF = 1.028. Largest peak and hole 1.879 (close to iridium atoms) and -1.870 e/ Å^3.

Crystal data for **6b** (CCDC 2077160): $C_{40}H_{30}Cl_2IrN_3$, Mw 815.77, red, irregular block (0.207 x 0.020 x 0.020 mm^3), monoclinic, space group P2$_1$/n, *a*: 8.8621(18) Å, *b*: 27.976(6) Å, *c*: 25.797(5) Å, *β*: 94.779(4)°, *V* = 6374(2) Å^3, *Z* = 8, *Z'* = 2, D_{calc}: 1.700 g cm^{-3}, F(000): 1.700, T

= 100(2) K, μ 4.392 mm^{-1}. 54547 measured reflections (2θ: 3-57°, ω scans 0.3°), 18482 unique (R_{int} = 0.1864); min./max. transm. Factors 0.681/0.862. Final agreement factors were R^1 = 0.0638 (8369 observed reflections, I >2σ(I)) and wR^2 = 0.1168; data/restraints/parameters 18482/78/831; GoF = 1.018. Largest peak and hole 2.410 (close to iridium atoms) and -1.799 e/ Å^3.

Crystal data for **8** (CCDC 2077164): $C_{39}H_{29}IrN_3$, BF_4, 1.5(CH_2Cl_2), M_W 946.05, red, irregular block (0.139 x 0.088 x 0.074 mm^3), triclinic, space group P-1, *a*: 10.4854(6) Å, *b*: 10.6732(6) Å, *c*: 16.4325(9) Å, α: 93.5670(10)°, β: 105.0910(10)°, γ: 93.2830(10)°, *V* = 1766.99(17) Å^3, *Z* = 2, *Z'* = 1, D_{calc}: 1.778 g cm^{-3}, F(000): 930, *T* = 100(2) K, μ 4.062 mm^{-1}. 31380 measured reflections (2θ: 3-57°, ω scans 0.3°), 8468 unique (R_{int} = 0.0319); min./max. transm. Factors 0.744/0.862. Final agreement factors were R^1 = 0.0286 (7803 observed reflections, I > 2σ(I)) and wR^2 = 0.0692; data/restraints/parameters 8468/12/492; GoF = 1.056. Largest peak and hole 1.863 (close to iridium atoms) and -1.930 e/ Å^3.

Crystal data for **9b** (CCDC 2077161): $C_{40}H_{30}IrN_3$, 2(CH_2Cl_2), M_W 914.72, red, irregular block (0.203 x 0.108 x 0.030 mm^3), triclinic, space group P-1, *a*: 10.5475(10) Å, *b*: 15.8828(14) Å, *c*: 21.2022(19) Å, α: 98.3830(10)°, β: 92.3710(10)°, γ: 95.7250(10)°, *V* = 3490.8(5) Å^3, *Z* = 4, *Z'* = 2, D_{calc}: 1.741 g cm^{-3}, F(000): 1808, *T* = 100(2) K, μ 4.168 mm^{-1}. 61506 measured reflections (2θ: 3-57°, ω scans 0.3°), 16688 unique (R_{int} = 0.0396); min./max. transm. Factors 0.627/0.862. Final agreement factors were R^1 = 0.0289 (13663 observed reflections, I > 2σ(I)) and wR^2 = 0.0679; data/restraints/parameters 16688/12/923; GoF = 1.014. Largest peak and hole 2.187 (close to iridium atoms) and -1.341 e/ Å^3.

Crystal data for **9c** (CCDC 2077162): $C_{40}H_{30}IrN_3$, 0.5(C_7H_8), M_W 790.93, red, irregular block (0.293 x 0.040 x 0.018 mm^3), monoclinic, space group P-1, *a*: 8.9737(12) Å, *b*: 12.0332(17) Å, *c*: 15.723(2) Å, α: 75.192(2)°, β: 80.628(2)°, γ: 84.594(2)°, *V* = 1617.0(4) Å^3, *Z* = 2, *Z'* = 1, D_{calc}: 1.624 g cm^{-3}, F(000): 786, *T* = 100(2) K, μ 4.166 mm^{-1}. 14496 measured reflections (2θ: 3-57°, ω scans 0.3°), 5699 unique (R_{int} = 0.0817); min./max. transm. Factors 0.584/0.862. Final agreement factors were R^1 = 0.0541 (4442 observed reflections, I > 2σ(I)) and wR^2 = 0.1119; data/restraints/parameters 5699/45/384350; GoF = 1.079. Largest peak and hole 2.119 (close to iridium atoms) and -3.215 e/ Å^3.

Computational Details. All calculations in the mechanistic studies were performed at the DFT level using the B3LYP B3LYP functional[3] supplemented with the Grimme's dispersion correction D3[4] as implemented in Gaussian09.[5] Ir atoms were described by means of an effective core potential SDD for the inner electron[6] and its associated double-ζ basis set for the outer ones, complemented with a set of f-polarization functions for iridium.[7] The 6-31G** basis set was used for the H, C, N, O and Cl.[8] All minima were verified to have no negative frequencies. The geometries were fully optimized in THF (ε = 7.4257) solvent using the continuum SMD model.[9] We performed TD-DFT calculations at the same level of theory in THF calculating the lowest 50 singlet-singlet excitations at the ground state S_0. It has to be noticed that the singlet-triplet excitations are set to zero due to the neglect of spin-orbit coupling in the TDDFT calculations as implemented in G09. The UV/vis absorption spectra were obtained by using the GaussSum 3 software.[10] The phosphorescence emission compares well with the 0-0 transition calculated taking into account the zero point energies (zpe) of the geometries of both the optimized S_0 and T_1 states in THF.

Energies of Optimized Structures of 3, 5, 6a-c, 8, 9b,c and 10

3, S_0 (THF)

Zero-point correction= 0.414828 (Hartree/Particle)
Thermal correction to Energy= 0.441437
Thermal correction to Enthalpy= 0.442381
Thermal correction to Gibbs Free Energy= 0.359606
Sum of electronic and zero-point Energies= -1866.343485
Sum of electronic and thermal Energies= -1866.316876
Sum of electronic and thermal Enthalpies= -1866.315931
Sum of electronic and thermal Free Energies= -1866.398707

3, T_1 (THF)

Zero-point correction= 0.411300 (Hartree/Particle)
Thermal correction to Energy= 0.438341
Thermal correction to Enthalpy= 0.439286
Thermal correction to Gibbs Free Energy= 0.354665
Sum of electronic and zero-point Energies= -1866.270711
Sum of electronic and thermal Energies= -1866.243670
Sum of electronic and thermal Enthalpies= -1866.242726
Sum of electronic and thermal Free Energies= -1866.327346

5, S_0 (THF)

Zero-point correction= 0.517324 (Hartree/Particle)
Thermal correction to Energy= 0.548598
Thermal correction to Enthalpy= 0.549542

Thermal correction to Gibbs Free Energy= 0.456787
Sum of electronic and zero-point Energies= -1637.890009
Sum of electronic and thermal Energies= -1637.858736
Sum of electronic and thermal Enthalpies= -1637.857792
Sum of electronic and thermal Free Energies= -1637.950546

5, T_1 (THF)

Zero-point correction= 0.513804 (Hartree/Particle)
Thermal correction to Energy= 0.545637
Thermal correction to Enthalpy= 0.546581
Thermal correction to Gibbs Free Energy= 0.451262
Sum of electronic and zero-point Energies= -1637.824149
Sum of electronic and thermal Energies= -1637.792316
Sum of electronic and thermal Enthalpies= -1637.791372
Sum of electronic and thermal Free Energies= -1637.886690

6a, S_0 (THF)

Zero-point correction= 0.564176 (Hartree/Particle)
Thermal correction to Energy= 0.596257
Thermal correction to Enthalpy= 0.597202
Thermal correction to Gibbs Free Energy= 0.503095
Sum of electronic and zero-point Energies= -1771.394914
Sum of electronic and thermal Energies= -1771.362833
Sum of electronic and thermal Enthalpies= -1771.361889
Sum of electronic and thermal Free Energies= -1771.455996

6a, T_1 (THF)

Zero-point correction= 0.560888 (Hartree/Particle)
Thermal correction to Energy= 0.593441
Thermal correction to Enthalpy= 0.594385
Thermal correction to Gibbs Free Energy= 0.497995
Sum of electronic and zero-point Energies= -1771.327196
Sum of electronic and thermal Energies= -1771.294643
Sum of electronic and thermal Enthalpies= -1771.293699
Sum of electronic and thermal Free Energies= -1771.390089

6b, S_0 (THF)

Zero-point correction= 0.564310 (Hartree/Particle)
Thermal correction to Energy= 0.596319
Thermal correction to Enthalpy= 0.597263
Thermal correction to Gibbs Free Energy= 0.503468
Sum of electronic and zero-point Energies= -1771.384340
Sum of electronic and thermal Energies= -1771.352331
Sum of electronic and thermal Enthalpies= -1771.351387
Sum of electronic and thermal Free Energies= -1771.445182

6b, T_1 (THF)

Zero-point correction= 0.560773 (Hartree/Particle)
Thermal correction to Energy= 0.593306
Thermal correction to Enthalpy= 0.594251
Thermal correction to Gibbs Free Energy= 0.498054
Sum of electronic and zero-point Energies= -1771.317028
Sum of electronic and thermal Energies= -1771.284494
Sum of electronic and thermal Enthalpies= -1771.283550
Sum of electronic and thermal Free Energies= -1771.379747

6c, S_0 (THF)

Zero-point correction= 0.564205 (Hartree/Particle)
Thermal correction to Energy= 0.596181
Thermal correction to Enthalpy= 0.597125
Thermal correction to Gibbs Free Energy= 0.503567
Sum of electronic and zero-point Energies= -1771.382130
Sum of electronic and thermal Energies= -1771.350154
Sum of electronic and thermal Enthalpies= -1771.349210
Sum of electronic and thermal Free Energies= -1771.442768

6c, T_1 (THF)

Zero-point correction= 0.560587 (Hartree/Particle)
Thermal correction to Energy= 0.593162
Thermal correction to Enthalpy= 0.594106
Thermal correction to Gibbs Free Energy= 0.497772
Sum of electronic and zero-point Energies= -1771.316936
Sum of electronic and thermal Energies= -1771.284361
Sum of electronic and thermal Enthalpies= -1771.283416
Sum of electronic and thermal Free Energies= -1771.379751

8, S_0 (THF)

Zero-point correction= 0.576966 (Hartree/Particle)
Thermal correction to Energy= 0.609515
Thermal correction to Enthalpy= 0.610459
Thermal correction to Gibbs Free Energy= 0.515411
Sum of electronic and zero-point Energies= -1771.866288
Sum of electronic and thermal Energies= -1771.833740
Sum of electronic and thermal Enthalpies= -1771.832795
Sum of electronic and thermal Free Energies= -1771.927843

8, T_1 (THF)

Zero-point correction= 0.573868 (Hartree/Particle)
Thermal correction to Energy= 0.606778
Thermal correction to Enthalpy= 0.607722

Thermal correction to Gibbs Free Energy= 0.510773
Sum of electronic and zero-point Energies= -1771.795690
Sum of electronic and thermal Energies= -1771.762780
Sum of electronic and thermal Enthalpies= -1771.761835
Sum of electronic and thermal Free Energies= -1771.858785

9b, S_0 (THF)

Zero-point correction= 0.591428 (Hartree/Particle)
Thermal correction to Energy= 0.625405
Thermal correction to Enthalpy= 0.626349
Thermal correction to Gibbs Free Energy= 0.527648
Sum of electronic and zero-point Energies= -1810.681824
Sum of electronic and thermal Energies= -1810.647847
Sum of electronic and thermal Enthalpies= -1810.646903
Sum of electronic and thermal Free Energies= -1810.745603

9b, T_1 (THF)

Zero-point correction= 0.588236 (Hartree/Particle)
Thermal correction to Energy= 0.622662
Thermal correction to Enthalpy= 0.623606
Thermal correction to Gibbs Free Energy= 0.522897
Sum of electronic and zero-point Energies= -1810.614390
Sum of electronic and thermal Energies= -1810.579964
Sum of electronic and thermal Enthalpies= -1810.579020
Sum of electronic and thermal Free Energies= -1810.679728

9c, S_0 (THF)

Zero-point correction= 0.591325 (Hartree/Particle)
Thermal correction to Energy= 0.625258
Thermal correction to Enthalpy= 0.626203
Thermal correction to Gibbs Free Energy= 0.527737
Sum of electronic and zero-point Energies= -1810.679700
Sum of electronic and thermal Energies= -1810.645767
Sum of electronic and thermal Enthalpies= -1810.644823
Sum of electronic and thermal Free Energies= -1810.743289

9c, T_1 (THF)

Zero-point correction= 0.588443 (Hartree/Particle)
Thermal correction to Energy= 0.622717
Thermal correction to Enthalpy= 0.623661
Thermal correction to Gibbs Free Energy= 0.523649
Sum of electronic and zero-point Energies= -1810.614143
Sum of electronic and thermal Energies= -1810.579869
Sum of electronic and thermal Enthalpies= -1810.578925
Sum of electronic and thermal Free Energies= -1810.678937

10, S_0 (THF)

Zero-point correction= 0.604765 (Hartree/Particle)
Thermal correction to Energy= 0.639240
Thermal correction to Enthalpy= 0.640184
Thermal correction to Gibbs Free Energy= 0.540034
Sum of electronic and zero-point Energies= -1811.162632
Sum of electronic and thermal Energies= -1811.128157
Sum of electronic and thermal Enthalpies= -1811.127213
Sum of electronic and thermal Free Energies= -1811.227363

10, T_1 (THF)

Zero-point correction= 0.600775 (Hartree/Particle)
Thermal correction to Energy= 0.635796
Thermal correction to Enthalpy= 0.636740
Thermal correction to Gibbs Free Energy= 0.534658
Sum of electronic and zero-point Energies= -1811.092799
Sum of electronic and thermal Energies= -1811.057777
Sum of electronic and thermal Enthalpies= -1811.056833
Sum of electronic and thermal Free Energies= -1811.158915

UV-vis Spectra of Complexes 3, 5, 6a-c, 8, 9b,c and 10 (Observed and Calculated)

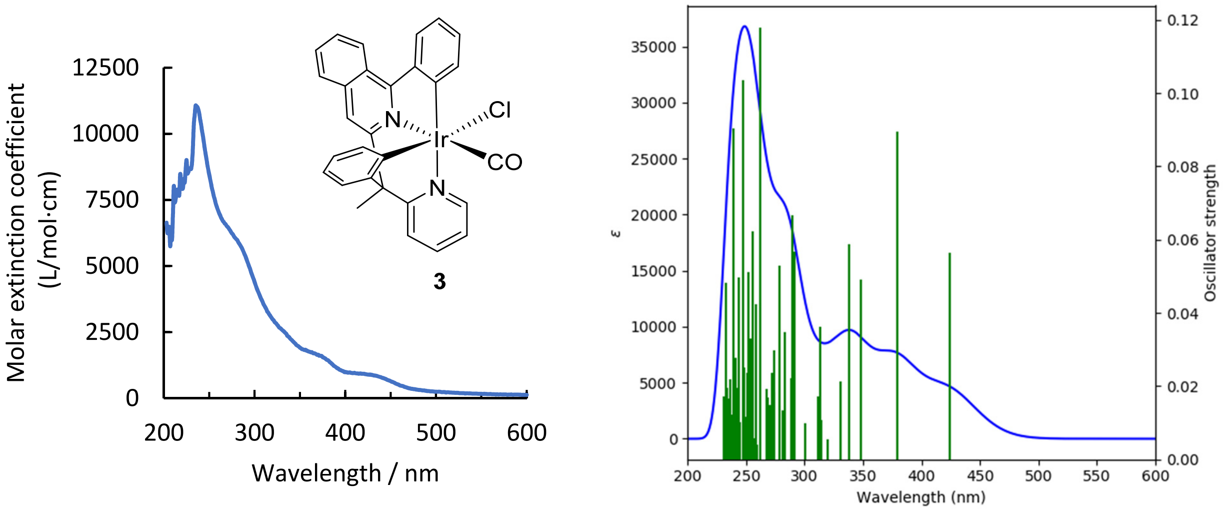

Figure S1. Observed UV-vis spectrum of complex **3** in 2-MeTHF (1.0 x 10^{-4} M) and calculated (B3LYP(GD3)//SDD(f)/6-31G**) in THF

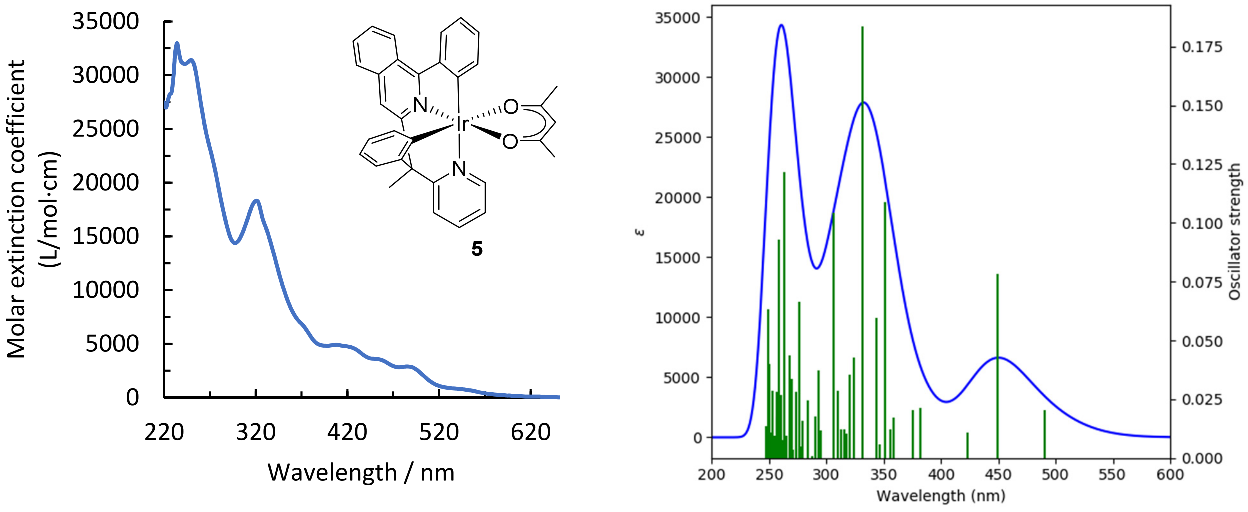

Figure S2. Observed UV-vis spectrum of complex **5** in 2-MeTHF (1.0 x 10^{-4} M) and calculated (B3LYP(GD3)//SDD(f)/6-31G**) in THF.

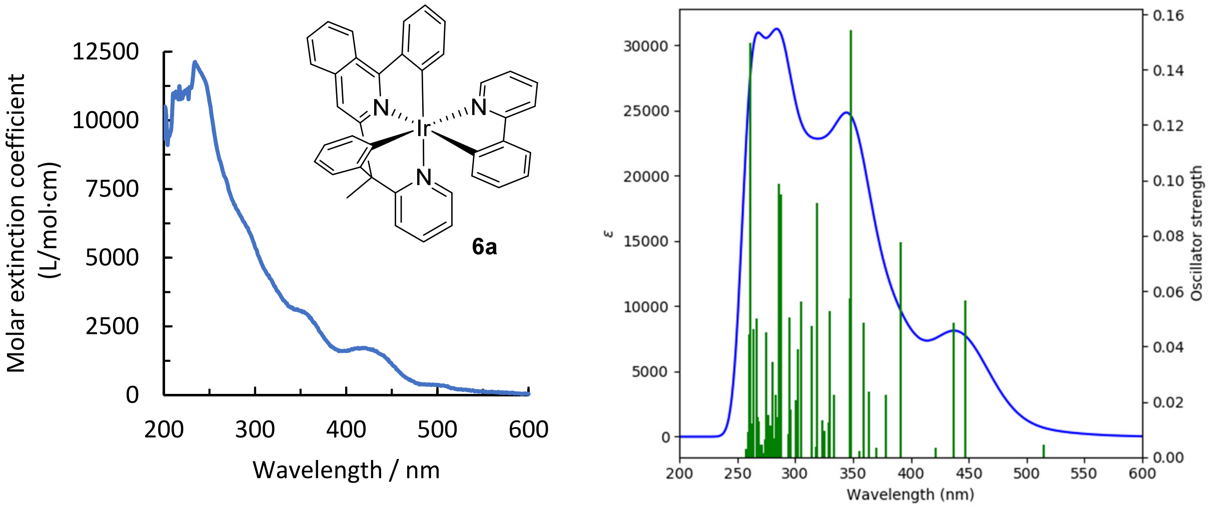

Figure S3. Observed UV-vis spectrum of complex **6a** in 2-MeTHF(1.0 x 10^{-4} M) and calculated (B3LYP(GD3)//SDD(f)/6-31G**) in THF

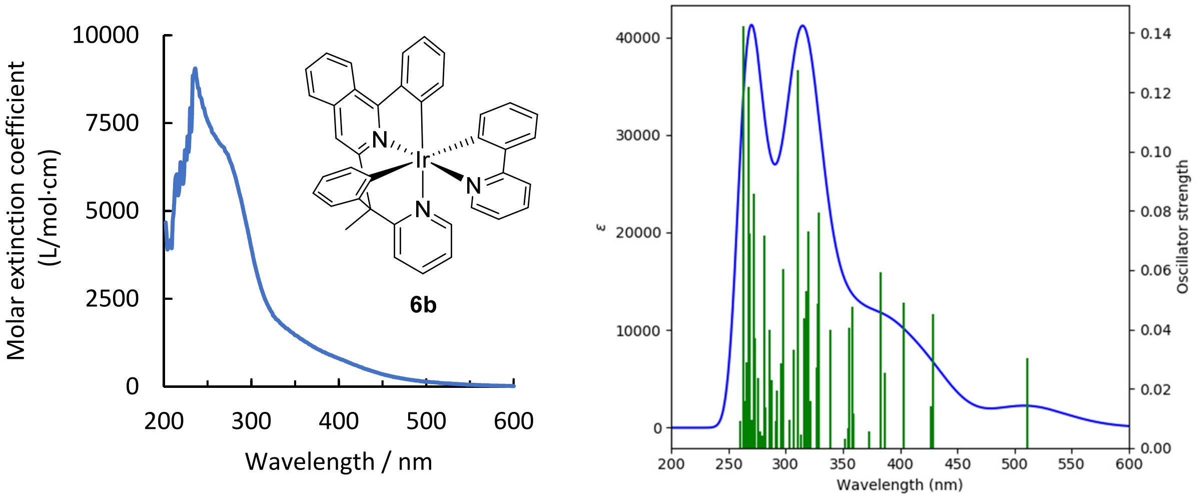

Figure S4. Observed UV-vis spectrum of complex **6b** in 2-MeTHF (1.0 x 10^{-4} M) and calculated (B3LYP(GD3)//SDD(f)/6-31G**) in THF.

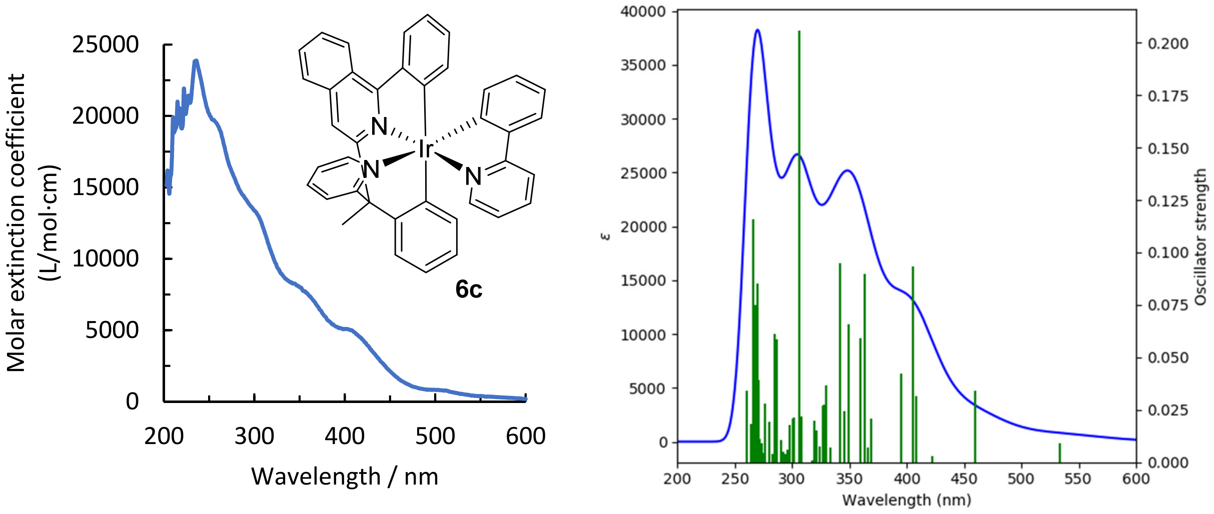

Figure S5. Observed UV-vis spectrum of complex **6c** in 2-MeTHF (1.0 x 10^{-4} M) and calculated (B3LYP(GD3)//SDD(f)/6-31G**) in THF.

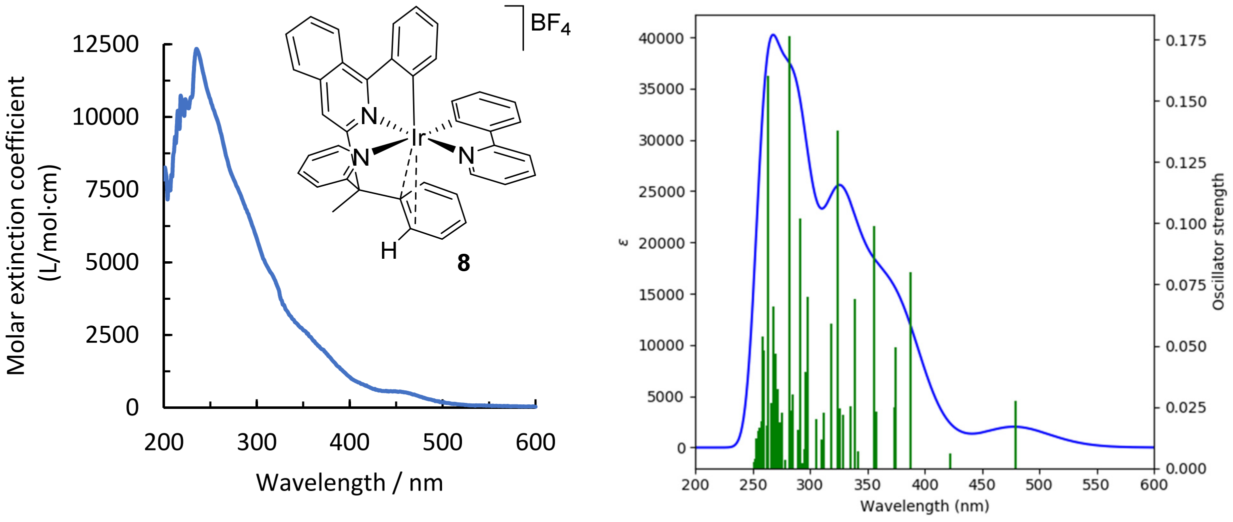

Figure S6. Observed UV-vis spectrum of complex **8** in 2-MeTHF (1.0 x 10^{-4} M) and calculated (B3LYP(GD3)//SDD(f)/6-31G**) in THF.

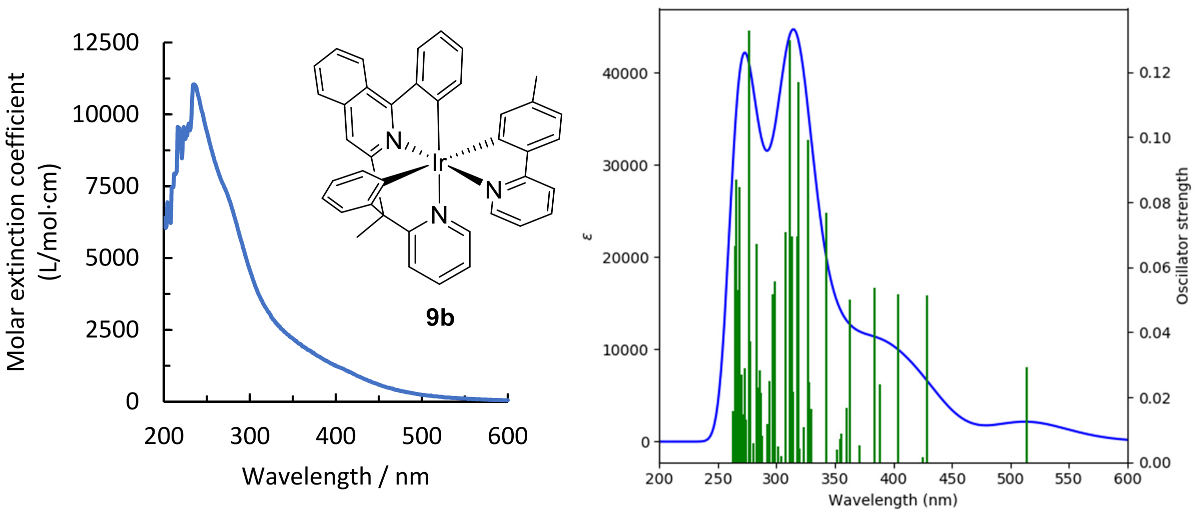

Figure S7. Observed UV-vis spectrum of complex **9b** in 2-MeTHF (1.0 x 10^{-4} M) and calculated (B3LYP(GD3)//SDD(f)/6-31G**) in THF.

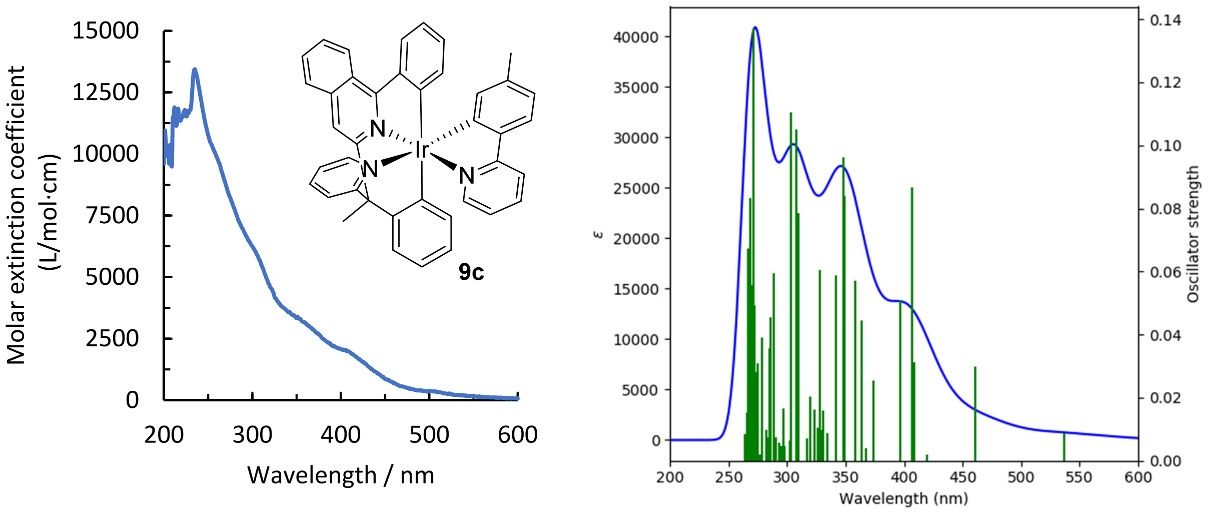

Figure S8. Observed UV-vis spectrum of complex **9c** in 2-MeTHF (1.0 x 10^{-4} M) and calculated (B3LYP(GD3)//SDD(f)/6-31G**) in THF.

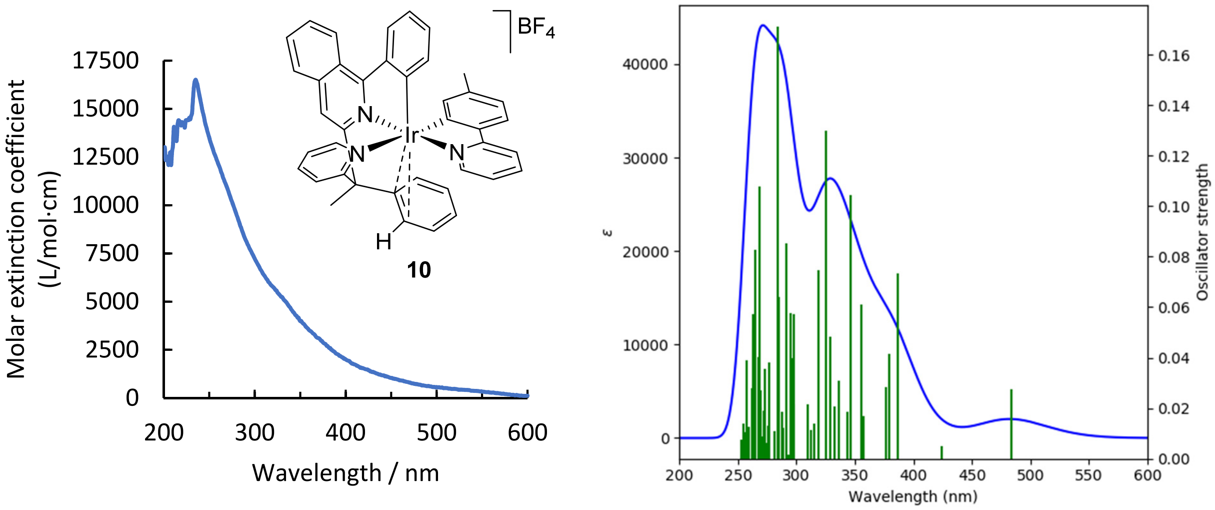

Figure S9. Observed UV-vis spectrum of complex **10** in 2-MeTHF (1.0 x 10^{-4} M) and calculated (B3LYP(GD3)//SDD(f)/6-31G**) in THF.

Analysis of Computed UV/Vis Data of Complexes 3, 5, 6a-c, 8, 9b-c, and 10

Selected transitions for the calculated UV spectra, energies, oscillator strengths, and molecular orbital contributions to the transitions are given in Tables S1, S3, S5, S7, S9, S11, S13, S15, and S17. In order to facilitate the understanding of the electronic transitions, an analysis of the change in charge density for each different group in which the molecules have been divided with the GaussSum program is included in Tables S2, S4, S6, S8, S10, S12, S14, S16, and S18.

Table S1: Selected transitions for the calculated UV spectrum of complex 3 in THF

No.	λ (nm)	Osc. Strength	Symmetry	Major contributions	Minor contributions
**1**	549	0	Triplet	HOMO->LUMO (44%), H-1->LUMO (42%)	H-2->LUMO (4%)
**3**	424	0.0565	Singlet	HOMO->LUMO (96%)	
**5**	379	0.0894	Singlet	H-1->LUMO (92%)	H-2->LUMO (5%)
**9**	348	0.0491	Singlet	H-2->LUMO (85%)	H-3->LUMO (4%), H-1->LUMO (4%), HOMO->L+1 (3%)
**11**	338	0.0588	Singlet	HOMO->L+1 (85%)	H-1->L+1 (5%), H-3->LUMO (2%)
**34**	289	0.0223	Singlet	H-2->L+1 (65%), HOMO->L+3 (14%)	H-3->L+1 (7%), H-6->LUMO (2%)
**59**	262	0.118	Singlet	H-9->LUMO (31%), H-3->L+2 (17%)	H-2->L+2 (9%), H-1->L+4 (7%), H-2->L+3 (5%), H-1->L+6 (3%)
**80**	248	0.1035	Singlet	H-6->L+1 (37%), H-1->L+6 (17%), H-1->L+5 (11%)	H-9->LUMO (4%), H-5->L+2 (4%), H-2->L+4 (3%), H-13->LUMO (2%)
**97**	234	0.0196	Singlet	H-4->L+3 (46%), HOMO->L+9 (10%)	H-1->L+7 (7%), HOMO->L+10 (7%), HOMO->L+8 (6%)

Table S2: Composition (%) of the selected transitions for the calculated UV-vis spectrum of complex 3.

No.	λ (nm)	Iridium	Ph	Py	Isoqui	*Ph*-Isoqui	C-Me	CO	Cl
**1**	549	20-->3 (-17)	19-->0 (-19)	1-->2 (1)	27-->73 (46)	26-->19 (-7)	0-->0 (0)	1-->2 (1)	6-->1 (-5)
**3**	424	29-->3 (-26)	14-->0 (-14)	1-->2 (1)	17-->73 (56)	35-->19 (-16)	0-->0 (0)	0-->2 (2)	4-->1 (-3)
**5**	379	12-->3 (-9)	25-->0 (-25)	1-->2 (1)	37-->73 (36)	17-->19 (2)	0-->0 (0)	1-->2 (1)	8-->1 (-7)
**9**	348	13-->3 (-10)	21-->0 (-21)	1-->4 (3)	14-->71 (57)	28-->19 (-9)	0-->0 (0)	2-->2 (0)	21-->1 (-20)
**11**	338	27-->4 (-23)	14-->1 (-13)	1-->72 (71)	18-->16 (-2)	34-->2 (-32)	0-->2 (2)	0-->2 (2)	5-->1 (-4)
**34**	289	15-->4 (-11)	19-->3 (-16)	1-->70 (69)	14-->16 (2)	29-->2 (-27)	0-->1 (1)	1-->3 (2)	21-->1 (-20)
**59**	262	13-->6 (-7)	13-->2 (-11)	7-->17 (10)	25-->57 (32)	16-->10 (-6)	0-->1 (1)	2-->7 (5)	24-->1 (-23)
**80**	248	7-->9 (2)	13-->3 (-10)	2-->39 (37)	39-->27 (-12)	29-->9 (-20)	0-->1 (1)	1-->11 (10)	8-->1 (-7)
**97**	234	12-->12 (0)	51-->22 (-29)	4-->39 (35)	11-->10 (-1)	14-->8 (-6)	0-->1 (1)	0-->9 (9)	7-->0 (-7)

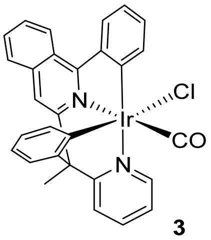

3

Table S3: Selected transitions for the calculated UV spectrum of complex 5 in THF.

No.	λ (nm)	Osc. Strength	Symmetry	Major contributions	Minor contributions
**1**	606	0	Triplet	H-1->LUMO (72%), HOMO->LUMO (10%)	H-3->LUMO (8%), H-2->LUMO (2%)
**3**	491	0.0205	Singlet	HOMO->LUMO (84%), H-1->LUMO (13%)	
**4**	449	0.0782	Singlet	H-1->LUMO (82%), HOMO->LUMO (14%)	
**7**	423	0.0109	Singlet	H-2->LUMO (94%)	
**11**	382	0.0213	Singlet	HOMO->L+1 (86%), H-1->L+1 (13%)	
**20**	351	0.1088	Singlet	H-3->LUMO (62%), H-1->L+3 (28%)	
**27**	332	0.1837	Singlet	H-1->L+3 (51%), H-3->LUMO (22%)	H-1->L+2 (7%), HOMO->L+3 (4%), HOMO->L+4 (2%), H-4->LUMO (2%)
**42**	307	0.1044	Singlet	H-2->L+3 (55%), H-6->LUMO (15%), H-1->L+4 (12%)	H-5->LUMO (2%), H-4->LUMO (2%), H-3->LUMO (2%)
**81**	264	0.1216	Singlet	H-9->LUMO (46%), H-3->L+3 (20%)	H-1->L+6 (4%), H-12->LUMO (4%), H-4->L+3 (3%), H-1->L+8 (2%)
**93**	253	0.0285	Singlet	H-7->L+1 (28%), H-1->L+7 (18%), H-5->L+2 (15%), H-1->L+8 (11%)	H-4->L+3 (6%), H-7->L+2 (3%), H-1->L+9 (3%), H-8->L+1 (2%)

Table S4: Composition (%) of the selected transitions for the calculated UV-vis spectrum of complex 5.

No.	λ (nm)	Iridium	Ph	Py	Isoqui	*Ph*-Isoqui	C-Me	acac
**1**	606	45-->5 (-40)	10-->0 (-10)	2-->1 (-1)	19-->74 (55)	13-->19 (6)	0-->0 (0)	12-->1 (-11)
**3**	491	44-->5 (-39)	18-->0 (-18)	2-->1 (-1)	7-->74 (67)	21-->19 (-2)	0-->0 (0)	7-->1 (-6)
**4**	449	48-->5 (-43)	11-->0 (-11)	2-->1 (-1)	16-->74 (58)	12-->19 (7)	0-->0 (0)	12-->1 (-11)
**7**	423	45-->5 (-40)	10-->0 (-10)	3-->1 (-2)	5-->74 (69)	6-->19 (13)	0-->0 (0)	30-->1 (-29)
**11**	382	44-->4 (-40)	19-->1 (-18)	2-->77 (75)	6-->10 (4)	22-->1 (-21)	0-->2 (2)	7-->5 (-2)
**20**	351	27-->4 (-23)	6-->1 (-5)	1-->8 (7)	40-->70 (30)	19-->14 (-5)	1-->0 (-1)	6-->3 (-3)
**27**	332	39-->3 (-36)	9-->1 (-8)	1-->17 (16)	25-->60 (35)	15-->6 (-9)	0-->1 (1)	10-->12 (2)
**42**	307	41-->3 (-38)	15-->2 (-13)	3-->24 (21)	7-->58 (51)	7-->5 (-2)	0-->1 (1)	27-->6 (-21)
**81**	264	19-->9 (-10)	8-->2 (-6)	9-->7 (-2)	32-->65 (33)	21-->14 (-7)	1-->0 (-1)	9-->3 (-6)
**93**	253	21-->4 (-17)	28-->18 (-10)	2-->32 (30)	11-->12 (1)	14-->14 (0)	0-->1 (1)	24-->19 (-5)

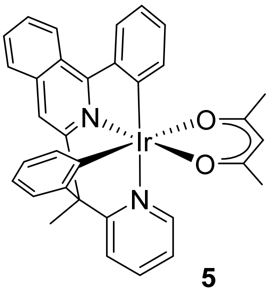

5

Table S5: Selected transitions for the calculated UV spectrum of complex 6a in THF.

No.	λ (nm)	Osc. Strength	Symmetry	Major contributions	Minor contributions
**1**	590	0	Triplet	HOMO->LUMO (48%), H-2->LUMO (24%), H-3->LUMO (12%)	H-1->LUMO (9%)
**2**	515	0.0044	Singlet	HOMO->LUMO (98%)	
**6**	447	0.0564	Singlet	H-1->LUMO (89%)	H-2->LUMO (7%)
**7**	437	0.0486	Singlet	H-2->LUMO (88%)	H-1->LUMO (8%)
**9**	422	0.0034	Singlet	HOMO->L+1 (96%)	
**12**	391	0.0778	Singlet	HOMO->L+2 (93%)	
**26**	349	0.1544	Singlet	H-3->LUMO (76%)	H-1->L+3 (7%), H-2->L+2 (3%), H-2->LUMO (2%)
**42**	319	0.092	Singlet	H-2->L+3 (60%), H-6->LUMO (17%)	H-5->LUMO (5%), H-2->L+4 (4%), H-1->L+4 (3%)
**66**	288	0.095	Singlet	H-4->LUMO+1 (51%), H-8->L (13%)	H-10->LUMO (7%), H-9->LUMO (6%), H-3->L+3 (6%), H-5->L+1 (4%)

Table S6: Composition (%) of the selected transitions for the calculated UV-vis spectrum of complex 6a.

No.	λ (nm)	Iridium	Tetradentate ligand					Ph-Py
			Ph	Py	Isoqui	*Ph*-Isoqui	C-Me	
**1**	590	44-->3 (-41)	9-->0 (-9)	3-->1 (-2)	12-->73 (61)	17-->19 (2)	0-->0 (0)	15-->4 (-11)
**2**	515	50-->3 (-47)	9-->0 (-9)	5-->1 (-4)	4-->73 (69)	15-->19 (4)	0-->0 (0)	16-->4 (-12)
**6**	447	46-->3 (-43)	24-->0 (-24)	1-->1 (0)	7-->73 (66)	6-->19 (13)	1-->0 (-1)	16-->4 (-12)
**7**	437	45-->3 (-42)	7-->0 (-7)	1-->1 (0)	14-->73 (59)	17-->19 (2)	0-->0 (0)	16-->4 (-12)
**9**	422	50-->4 (-46)	9-->1 (-8)	5-->2 (-3)	4-->3 (-1)	15-->0 (-15)	0-->0 (0)	16-->90 (74)
**12**	391	50-->4 (-46)	9-->1 (-8)	5-->78 (73)	4-->11 (7)	15-->1 (-14)	0-->1 (1)	16-->4 (-12)
**26**	349	19-->3 (-16)	8-->0 (-8)	1-->5 (4)	39-->69 (30)	24-->17 (-7)	1-->0 (-1)	8-->5 (-3)
**42**	319	36-->3 (-33)	20-->1 (-19)	1-->13 (12)	12-->58 (46)	16-->5 (-11)	0-->1 (1)	14-->20 (6)
**66**	288	10-->3 (-7)	7-->1 (-6)	3-->3 (0)	11-->27 (16)	10-->6 (-4)	0-->0 (0)	60-->60 (0)

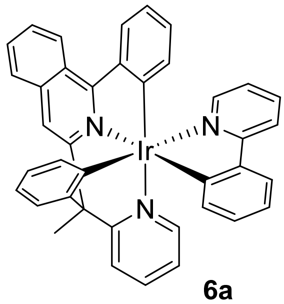

6a

Table S7: Selected transitions for the calculated UV spectrum of complex 6b in THF.

No.	λ (nm)	Osc. Strength	Symmetry	Major contributions	Minor contributions
**1**	593	0	Triplet	HOMO->LUMO (45%), H-1->LUMO (37%)	H-4->LUMO (3%), H-3->LUMO (3%), H-5->LUMO (2%), H-2->LUMO (2%)
**3**	511	0.0304	Singlet	HOMO->LUMO (98%)	
**5**	429	0.045	Singlet	H-1->LUMO (64%), HOMO->L+1 (31%)	
**10**	403	0.0491	Singlet	H-2->LUMO (93%)	
**15**	383	0.0593	Singlet	H-3->LUMO (42%), HOMO->L+2 (37%), H-4->LUMO (13%)	
**36**	329	0.0794	Singlet	H-3->L+1 (52%), HOMO->L+5 (17%), H-2->L+2 (14%)	H-4->L+1 (9%)
**52**	310	0.1274	Singlet	H-8->LUMO (48%), H-1->L+3 (12%), H-1->L+4 (10%)	H-3->L+2 (7%), H-3->L+1 (5%), H-2->L+4 (4%), H-1->L+5 (2%)
**96**	266	0.0289	Singlet	H-7->L+2 (49%), H-8->L+2 (28%), H-6->L+2 (10%)	H-5->L+2 (2%), H-5->L+3 (2%)
**99**	263	0.1424	Singlet	H-5->L+3 (57%)	H-7->L+2 (6%), HOMO->L+9 (6%), H-12->LUMO (5%), H-8->L+2 (3%), H-1->L+6 (2%)

Table S8: Composition (%) of the selected transitions for the calculated UV-vis spectrum of complex 6b.

No.	λ (nm)	Iridium	Tetradentate ligand					Ph-Py
			Ph	Py	Isoqui	*Ph*-Isoqui	C-Me	
**1**	593	42-->3 (-39)	14-->1 (-13)	2-->1 (-1)	13-->68 (55)	16-->19 (3)	0-->0 (0)	12-->8 (-4)
**3**	511	45-->3 (-42)	10-->1 (-9)	2-->1 (-1)	5-->68 (63)	24-->19 (-5)	0-->0 (0)	13-->8 (-5)
**5**	429	44-->4 (-40)	17-->1 (-16)	1-->1 (0)	17-->48 (31)	13-->13 (0)	0-->0 (0)	8-->33 (25)
**10**	403	13-->3 (-10)	37-->1 (-36)	3-->1 (-2)	5-->68 (63)	7-->19 (12)	0-->0 (0)	35-->8 (-27)
**15**	383	43-->3 (-40)	7-->1 (-6)	2-->34 (32)	10-->43 (33)	17-->12 (-5)	0-->1 (1)	20-->6 (-14)
**36**	329	39-->4 (-35)	11-->2 (-9)	3-->28 (25)	11-->7 (-4)	11-->7 (-4)	0-->0 (0)	23-->57 (34)
**52**	310	29-->3 (-26)	34-->1 (-33)	2-->12 (10)	11-->49 (38)	6-->11 (5)	0-->0 (0)	17-->23 (6)
**96**	266	11-->3 (-8)	36-->2 (-34)	2-->83 (81)	5-->5 (0)	15-->1 (-14)	0-->2 (2)	30-->4 (-26)
**99**	263	17-->3 (-14)	9-->7 (-2)	3-->18 (15)	22-->46 (24)	19-->4 (-15)	0-->1 (1)	30-->21 (-9)

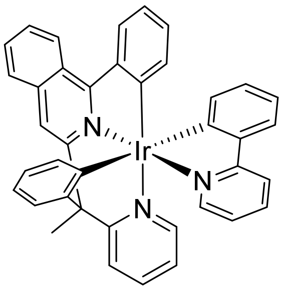

6b

Table S9: Selected transitions for the calculated UV spectrum of complex 6c in THF.

No.	λ (nm)	Osc. Strength	Symmetry	Major contributions	Minor contributions
**1**	608	0	Triplet	HOMO->LUMO (59%), H-2->LUMO (32%)	H-5->LUMO (2%)
**2**	533	0.0092	Singlet	HOMO->LUMO (98%)	
**6**	460	0.0343	Singlet	H-1->LUMO (97%)	
**12**	406	0.0932	Singlet	H-2->LUMO (89%)	H-5->LUMO (4%)
**25**	359	0.059	Singlet	H-1->L+2 (77%), HOMO->L+4 (15%)	HOMO->L+3 (4%)
**55**	306	0.2056	Singlet	H-4->L+1 (42%), H-2->L+3 (32%)	H-3->L+1 (7%), H-2->L+1 (4%), H-5->L+1 (3%)
**97**	267	0.0619	Singlet	H-8->L+2 (45%), H-7->L+2 (25%)	H-7->L+1 (9%), H-4->L+4 (3%) H-10->LUMO (2%), H-6->L+1 (2%)
**98**	266	0.1158	Singlet	H-5->L+3 (53%), H-10->LUMO (15%)	H-7->L+2 (6%), H-4->L+3 (5%), H-13->LUMO (5%)

Table S10: Composition (%) of the selected transitions for the calculated UV-vis spectrum of complex 6c.

No.	λ (nm)	Iridium	Tetradentate ligand					Ph-Py
			Ph	Py	Isoqui	*Ph*-Isoqui	C-Me	
**1**	608	43-->3 (-40)	14-->1 (-13)	2-->1 (-1)	16-->71 (55)	15-->19 (4)	0-->0 (0)	10-->5 (-5)
**2**	533	50-->3 (-47)	20-->1 (-19)	2-->1 (-1)	5-->71 (66)	13-->19 (6)	0-->0 (0)	10-->5 (-5)
**6**	460	26-->3 (-23)	21-->1 (-20)	0-->1 (1)	1-->71 (70)	20-->19 (-1)	1-->0 (-1)	31-->5 (-26)
**12**	406	33-->3 (-30)	4-->1 (-3)	2-->1 (-1)	35-->71 (36)	18-->19 (1)	0-->0 (0)	8-->5 (-3)
**25**	359	30-->5 (-25)	21-->1 (-20)	1-->40 (39)	2-->11 (9)	19-->0 (-19)	0-->1 (1)	27-->42 (15)
**55**	306	27-->2 (-25)	20-->1 (-19)	2-->30 (28)	18-->23 (5)	13-->2 (-11)	0-->1 (1)	20-->41 (21)
**97**	267	15-->5 (-10)	25-->1 (-24)	3-->42 (39)	6-->11 (5)	22-->1 (-21)	0-->1 (1)	28-->39 (11)
**98**	266	22-->3 (-19)	14-->1 (-13)	6-->23 (17)	22-->51 (29)	10-->6 (-4)	0-->1 (1)	26-->17 (-9)

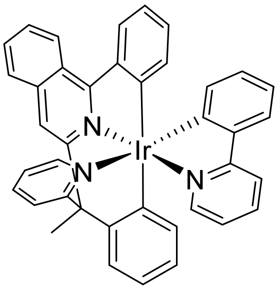

6c

Table S11: Selected transitions for the calculated UV spectrum of complex 8 in THF.

No.	λ (nm)	Osc. Strength	Symmetry	Major contributions	Minor contributions
**1**	572	0	Triplet	HOMO->LUMO (53%), H-1->LUMO (36%)	
**3**	479	0.0274	Singlet	HOMO->LUMO (95%)	HOMO->L+1 (3%)
**9**	387	0.0801	Singlet	H-1->LUMO (85%)	H-2->LUMO (7%)
**19**	356	0.0987	Singlet	H-3->LUMO (45%), H-1->L+1 (22%)	H-2->LUMO (8%), H-2->L+1 (6%), H-4->LUMO (4%), H-4->L+1 (2%), H-1->L+2 (2%)
**24**	339	0.0691	Singlet	H-4->LUMO (57%), H-3->LUMO (13%)	HOMO->L+3 (7%), H-5->LUMO (6%), H-2->LUMO (4%), H-2->L+1 (4%)
**32**	324	0.1378	Singlet	H-3->L+1 (51%), H-1->L+1 (13%), H-2->L+1 (11%)	H-4->L+1 (6%), HOMO->L+3 (5%), H-5->L+1 (2%)
**34**	319	0.0501	Singlet	H-1->L+2 (71%)	H-5->LUMO (7%), H-2->L+2 (4%), HOMO->L+5 (3%), HOMO->L+6 (2%)
**55**	291	0.1021	Singlet	H-7->LUMO (55%), H-1->L+3 (17%)	H-5->L+1 (7%), H-1->L+4 (4%)
**64**	282	0.1765	Singlet	H-4->L+2 (29%), H-8->LUMO (16%)	H-1->L+4 (9%), H-3->L+2 (5%), H-2->L+6 (4%), H-5->L+2 (3%), H-2->L+2 (3%), H-2->L+4 (3%), H-2->L+5 (3%)
**97**	254	0.0107	Singlet	H-6->L+2 (54%), H-2->L+6 (12%)	H-3->L+5 (6%), H-3->L+6 (3%), H-4->L+4 (3%), H-2->L+5 (3%)

Table S12: Composition (%) of the selected transitions for the calculated UV-vis spectrum of complex 8.

No.	λ (nm)	Iridium	Tetradentate ligand					Ph-Py
			Ph	Py	Isoqui	*Ph*-Isoqui	C-Me	
**1**	572	34-->5 (-29)	1-->1 (0)	1-->1 (0)	20-->66 (46)	20-->20 (0)	0-->0 (0)	23-->6 (-17)
**3**	479	44-->6 (-38)	1-->1 (0)	2-->2 (0)	8-->65 (57)	23-->19 (-4)	0-->0 (0)	22-->7 (-15)
**9**	387	22-->5 (-17)	2-->1 (-1)	1-->1 (0)	35-->66 (31)	16-->20 (4)	0-->0 (0)	24-->6 (-18)
**19**	356	32-->7 (-25)	9-->2 (-7)	1-->10 (9)	17-->47 (30)	14-->14 (0)	0-->0 (0)	26-->20 (-6)
**24**	339	29-->5 (-24)	3-->1 (-2)	2-->5 (3)	15-->62 (47)	11-->18 (7)	0-->0 (0)	41-->9 (-32)
**32**	324	35-->10 (-25)	10-->3 (-7)	2-->26 (24)	13-->14 (1)	15-->3 (-12)	0-->1 (1)	27-->43 (16)
**34**	319	23-->5 (-18)	2-->3 (1)	1-->42 (41)	33-->16 (-17)	16-->2 (-14)	0-->1 (1)	25-->31 (6)
**55**	291	8-->5 (-3)	8-->1 (-7)	1-->12 (11)	36-->54 (18)	36-->14 (-22)	1-->0 (-1)	10-->14 (4)
**64**	282	25-->5 (-20)	13-->6 (-7)	2-->28 (26)	17-->23 (6)	11-->6 (-5)	1-->1 (0)	31-->32 (1)
**97**	254	14-->6 (-8)	57-->9 (-48)	3-->40 (37)	5-->12 (7)	7-->2 (-5)	1-->1 (0)	14-->30 (16)

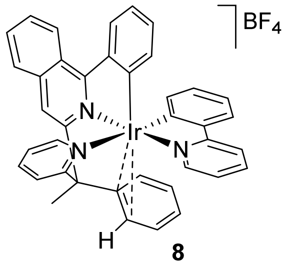

8

Table S13: Selected transitions for the calculated UV spectrum of complex 9b in THF.

No.	λ (nm)	Osc. Strength	Symmetry	Major contributions	Minor contributions
**1**	594	0	Triplet	HOMO->LUMO (47%), H-1->LUMO (35%)	H-3->LUMO (5%), H-5->LUMO (3%), H-2->LUMO (2%)
**3**	514	0.0293	Singlet	HOMO->LUMO (98%)	
**5**	429	0.0513	Singlet	H-1->LUMO (91%)	HOMO->L+1 (2%)
**10**	405	0.0517	Singlet	H-2->LUMO (92%)	
**15**	384	0.0538	Singlet	H-3->LUMO (35%), H-4->LUMO (32%), HOMO->L+2 (25%)	
**21**	363	0.0501	Singlet	H-4->LUMO (49%), H-3->LUMO (37%)	HOMO->L+3 (6%)
**45**	318	0.117	Singlet	H-7->LUMO (55%), H-1->L+3 (13%)	H-4->L+1 (8%), H-8->LUMO (6%), H-6->LUMO (3%), H-1->L+4 (3%), H-5->LUMO (2%), H-3->L+1 (2%)
**87**	277	0.1329	Singlet	H-6->L+1 (32%), H-3->L+4 (25%), H-4->L+4 (18%)	H-7->L+1 (6%), H-6->L+2 (5%)
**96**	268	0.016	Singlet	H-7->L+2 (56%), H-6->L+2 (13%)	H-8->L+2 (8%), HOMO->L+8 (8%), H-5->L+3 (3%)

Table S14: Composition (%) of the selected transitions for the calculated UV-vis spectrum of complex 9b.

No.	λ (nm)	Iridium	Tetradentate ligand					*p*-Tolyl-Py
			Ph	Py	Isoqui	*Ph*-Isoqui	C-Me	
**1**	594	42-->3 (-39)	14-->1 (-13)	1-->1 (0)	12-->69 (57)	16-->19 (3)	0-->0 (0)	14-->7 (-7)
**3**	514	45-->3 (-42)	10-->1 (-9)	2-->1 (-1)	5-->69 (64)	23-->19 (-4)	0-->0 (0)	15-->7 (-8)
**5**	429	44-->3 (-41)	21-->1 (-20)	1-->1 (0)	20-->68 (48)	8-->18 (10)	0-->0 (0)	6-->9 (3)
**10**	405	12-->3 (-9)	33-->1 (-32)	2-->1 (-1)	5-->69 (64)	8-->19 (11)	0-->0 (0)	39-->7 (-32)
**15**	384	36-->3 (-33)	9-->1 (-8)	2-->23 (21)	8-->51 (43)	13-->14 (1)	0-->1 (1)	32-->6 (-26)
**21**	363	35-->3 (-32)	8-->1 (-7)	2-->1 (-1)	7-->69 (62)	10-->18 (8)	0-->0 (0)	37-->8 (-29)
**45**	318	17-->3 (-14)	37-->1 (-36)	2-->3 (1)	8-->60 (52)	16-->14 (-2)	0-->0 (0)	20-->19 (-1)
**87**	277	17-->4 (-13)	26-->1 (-25)	3-->7 (4)	7-->11 (4)	11-->0 (-11)	0-->0 (0)	36-->77 (41)
**96**	268	11-->4 (-7)	41-->4 (-37)	3-->74 (71)	6-->6 (0)	18-->2 (-16)	0-->1 (1)	21-->9 (-12)

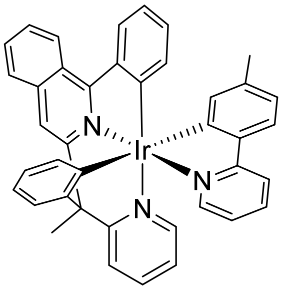

9b

Table S15: Selected transitions for the calculated UV spectrum of complex 9c in THF.

No.	λ (nm)	Osc. Strength	Symmetry	Major contributions	Minor contributions
**1**	611	0	Triplet	HOMO->LUMO (59%), H-2->LUMO (32%)	
**2**	537	0.0087	Singlet	HOMO->LUMO (98%)	
**6**	461	0.0298	Singlet	H-1->LUMO (96%)	
**11**	407	0.0868	Singlet	H-2->LUMO (86%)	H-5->LUMO (4%)
**25**	358	0.0572	Singlet	H-1->L+2 (66%), HOMO->L+4 (27%)	HOMO->L+3 (2%)
**54**	308	0.1049	Singlet	H-2->L+3 (62%), H-4->L+2 (11%)	H-3->L+3 (4%), H-2->L+2 (3%), H-8->LUMO (3%), H-5->LUMO (2%), H-5->L+2 (2%)
**58**	303	0.1104	Singlet	H-4->L+2 (44%), H-3->L+2 (23%), H-2->L+3 (13%)	H-5->L+2 (5%), H-2->L+2 (4%)
**97**	268	0.1158	Singlet	H-5->L+3 (52%), H-10->LUMO (23%)	HOMO->L+8 (5%), H-13->LUMO (5%), H-4->L+3 (4%)
**98**	267	0.0672	Singlet	H-8->L+2 (72%)	H-7->L+2 (4%), HOMO->L+9 (4%)

Table S16: Composition (%) of the selected transitions for the calculated UV-vis spectrum of complex 9c.

No.	λ (nm)	Iridium	Tetradentate ligand					*p*-Tolyl-Py
			Ph	Py	Isoqui	*Ph*-Isoqui	C-Me	
**1**	611	43-->3 (-40)	13-->1 (-12)	2-->1 (-1)	15-->72 (57)	15-->19 (4)	0-->0 (0)	11-->4 (-7)
**2**	537	50-->3 (-47)	19-->1 (-18)	2-->1 (-1)	4-->72 (68)	13-->19 (6)	0-->0 (0)	13-->4 (-9)
**6**	461	25-->3 (-22)	21-->1 (-20)	0-->1 (1)	1-->72 (71)	20-->19 (-1)	1-->0 (-1)	32-->4 (-28)
**11**	407	31-->3 (-28)	4-->1 (-3)	2-->1 (-1)	34-->72 (38)	18-->19 (1)	0-->0 (0)	10-->4 (-6)
**25**	358	33-->5 (-28)	20-->1 (-19)	1-->25 (24)	2-->9 (7)	18-->0 (-18)	0-->1 (1)	26-->60 (34)
**54**	308	29-->3 (-26)	9-->1 (-8)	2-->28 (26)	28-->45 (17)	18-->3 (-15)	0-->1 (1)	13-->20 (7)
**58**	303	24-->5 (-19)	23-->1 (-22)	2-->32 (30)	11-->14 (3)	16-->0 (-16)	0-->1 (1)	24-->48 (24)
**97**	268	26-->3 (-23)	7-->3 (-4)	7-->19 (12)	23-->55 (32)	9-->10 (1)	0-->1 (1)	27-->11 (-16)
**98**	267	21-->5 (-16)	25-->3 (-22)	3-->31 (28)	9-->7 (-2)	34-->1 (-33)	0-->1 (1)	9-->52 (43)

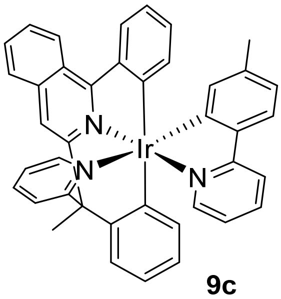

9c

Table S17: Selected transitions for the calculated UV spectrum of complex 10 in THF.

No.	λ (nm)	Osc. Strength	Symmetry	Major contributions	Minor contributions
**1**	573	0	Triplet	HOMO->LUMO (52%), H-1->LUMO (37%)	
**3**	483	0.0277	Singlet	HOMO->LUMO (94%)	HOMO->L+1 (3%)
**9**	387	0.0732	Singlet	H-1->LUMO (84%)	H-2->LUMO (7%)
**19**	356	0.0609	Singlet	H-1->L+1 (50%), H-3->LUMO (16%), H-2->L+1 (14%)	H-4->L+1 (6%), H-2->LUMO (2%), H-1->LUMO (2%)
**22**	346	0.1042	Singlet	H-4->LUMO (47%), H-3->LUMO (28%)	H-2->LUMO (8%), H-2->L+1 (3%), HOMO->L+4 (2%)
**32**	324	0.1299	Singlet	H-3->L+1 (37%), H-4->L+1 (20%), H-1->L+1 (11%)	H-2->L+1 (6%), HOMO->L+5 (5%), H-5->L+1 (4%), HOMO->L+3 (4%)
**34**	319	0.0747	Singlet	H-1->L+2 (66%), HOMO->L+5 (12%)	H-5->LUMO (6%), H-2->L+2 (3%)
**64**	284	0.1712	Singlet	H-8->LUMO (47%), H-1->L+4 (10%)	H-1->L+3 (5%), H-2->L+4 (4%), H-2->L+5 (3%), H-3->L+2 (3%), H-3->L+4 (3%), H-3->L+5 (3%),
**91**	263	0.0513	Singlet	H-8->L+1 (47%), H-7->L+1 (19%)	H-2->L+5 (7%), H-1->L+6 (5%), HOMO->L+11 (4%), H-3->L+4 (3%)

Table S18: Composition (%) of the selected transitions for the calculated UV-vis spectrum of complex 10.

No.	λ (nm)	Iridium	Tetradentate ligand					*p*-Tolyl-Py
			Ph	Py	Isoqui	*Ph*-Isoqui	C-Me	
**1**	573	31-->6 (-25)	1-->1 (0)	1-->1 (0)	20-->67 (47)	19-->20 (1)	0-->0 (0)	28-->5 (-23)
**3**	483	42-->6 (-36)	0-->1 (1)	2-->2 (0)	7-->66 (59)	22-->20 (-2)	0-->0 (0)	27-->6 (-21)
**9**	387	17-->6 (-11)	1-->1 (0)	1-->1 (0)	35-->67 (32)	15-->20 (5)	0-->0 (0)	31-->5 (-26)
**19**	356	23-->9 (-14)	4-->3 (-1)	1-->24 (23)	25-->25 (0)	14-->7 (-7)	0-->1 (1)	32-->31 (-1)
**22**	346	34-->6 (-28)	6-->1 (-5)	1-->2 (1)	11-->64 (53)	12-->19 (7)	0-->0 (0)	35-->8 (-27)
**32**	324	34-->10 (-24)	8-->6 (-2)	1-->30 (29)	15-->13 (-2)	13-->3 (-10)	0-->1 (1)	29-->37 (8)
**34**	319	20-->6 (-14)	1-->7 (6)	1-->34 (33)	31-->12 (-19)	17-->3 (-14)	0-->1 (1)	29-->37 (8)
**64**	284	20-->6 (-14)	33-->6 (-27)	1-->7 (6)	17-->44 (27)	11-->13 (2)	2-->0 (-2)	16-->24 (8)
**91**	263	16-->10 (-6)	32-->7 (-25)	1-->28 (27)	20-->12 (-8)	19-->5 (-14)	2-->1 (-1)	11-->37 (26)

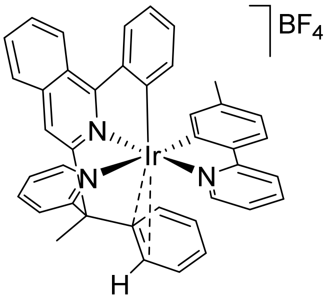

10

Theoretical Analysis of Molecular Orbitals of Complexes 3, 5, 6a-c, 8, 9b,c, and 10.

Energies and population analysis (%) of frontier molecular orbitals are given in Tables S19–S27 whereas Figures S10–S19 collects the frontier molecular orbitals and natural transition orbitals (NTOs) for the T_1 excited states.

Table S19: Composition of the frontier orbitals of 3

MO	eV	Iridium	Ph	Py	isoqui	*Ph*-isoqui	C-Me	CO	Cl
**L+9**	0.24	10	63	1	9	10	1	4	0
**L+8**	0.19	29	12	2	5	44	0	6	1
**L+7**	0	38	34	4	3	4	0	16	0
**L+6**	-0.25	16	4	5	26	13	0	31	0
**L+5**	-0.28	22	9	8	27	23	0	17	0
**L+4**	-0.39	31	4	11	14	8	0	33	3
**L+3**	-0.70	5	13	62	12	1	1	6	0
**L+2**	-1.05	3	2	22	60	1	1	4	0
**L+1**	-1.36	4	1	71	15	2	2	2	1
**LUMO**	-2.09	3	0	12	73	19	0	2	1
**HOMO**	-5.70	29	14	1	17	35	0	0	4
**H-1**	-5.93	12	25	1	38	16	0	1	7
**H-2**	-6.29	12	22	0	13	29	0	2	20
**H-3**	-6.53	11	4	1	2	17	0	1	64
**H-4**	-6.60	2	84	5	4	2	1	0	3
**H-5**	-6.67	8	25	7	8	7	0	2	44
**H-6**	-6.92	1	4	8	45	44	0	0	5
**H-7**	-7.17	11	24	16	12	14	1	2	25
**H-8**	-7.20	25	8	77	26	6	1	5	21
**H-9**	-7.43	16	8	11	37	9	0	3	12

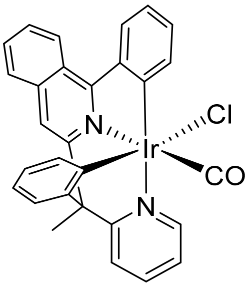

3

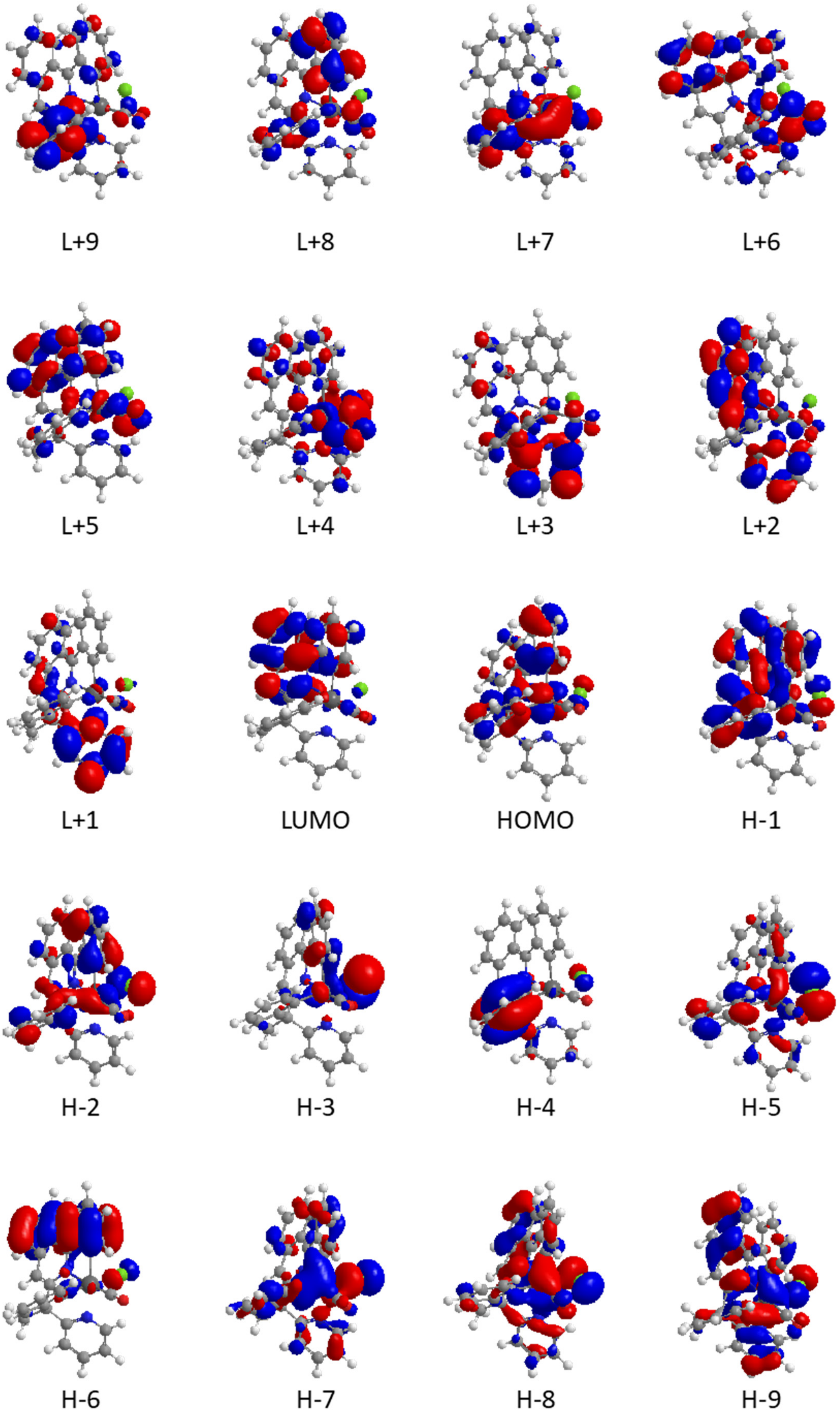

Figure S10. Frontier molecular orbitals of complex **3** (isovalue 0.03 au).

Table S20: Composition of the frontier orbitals of complex 5

MO	eV	Iridium	Ph	Py	isoqui	*Ph*-isoqui	C-Me	acac
**L+9**	0.67	7	77	1	1	13	1	1
**L+8**	0.47	4	45	7	9	34	0	0
**L+7**	0.43	5	43	3	7	41	0	1
**L+6**	0.11	96	0	0	0	1	0	2
**L+5**	-0.11	3	2	1	58	37	0	0
**L+4**	-0.49	2	11	72	12	0	1	1
**L+3**	-0.80	3	1	23	62	1	1	9
**L+2**	-0.85	3	1	6	8	0	0	81
**L+1**	-1.12	4	1	77	10	1	2	5
**LUMO**	-1.79	5	0	1	74	19	0	1
**HOMO**	-5.06	44	20	2	5	23	0	6
**H-1**	-5.16	48	9	1	18	10	0	13
**H-2**	-5.49	45	10	3	5	6	0	30
**H-3**	-5.95	18	4	2	49	23	1	4
**H-4**	-6.23	9	20	0	7	35	0	29
**H-5**	-6.29	2	88	4	2	1	1	2
**H-6**	-6.44	31	29	4	2	4	0	31
**H-7**	-6.67	6	22	2	6	18	1	46
**H-8**	-6.78	2	5	1	46	37	0	8
**H-9**	-7.08	17	9	15	26	23	1	9

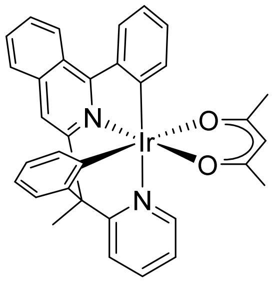

5

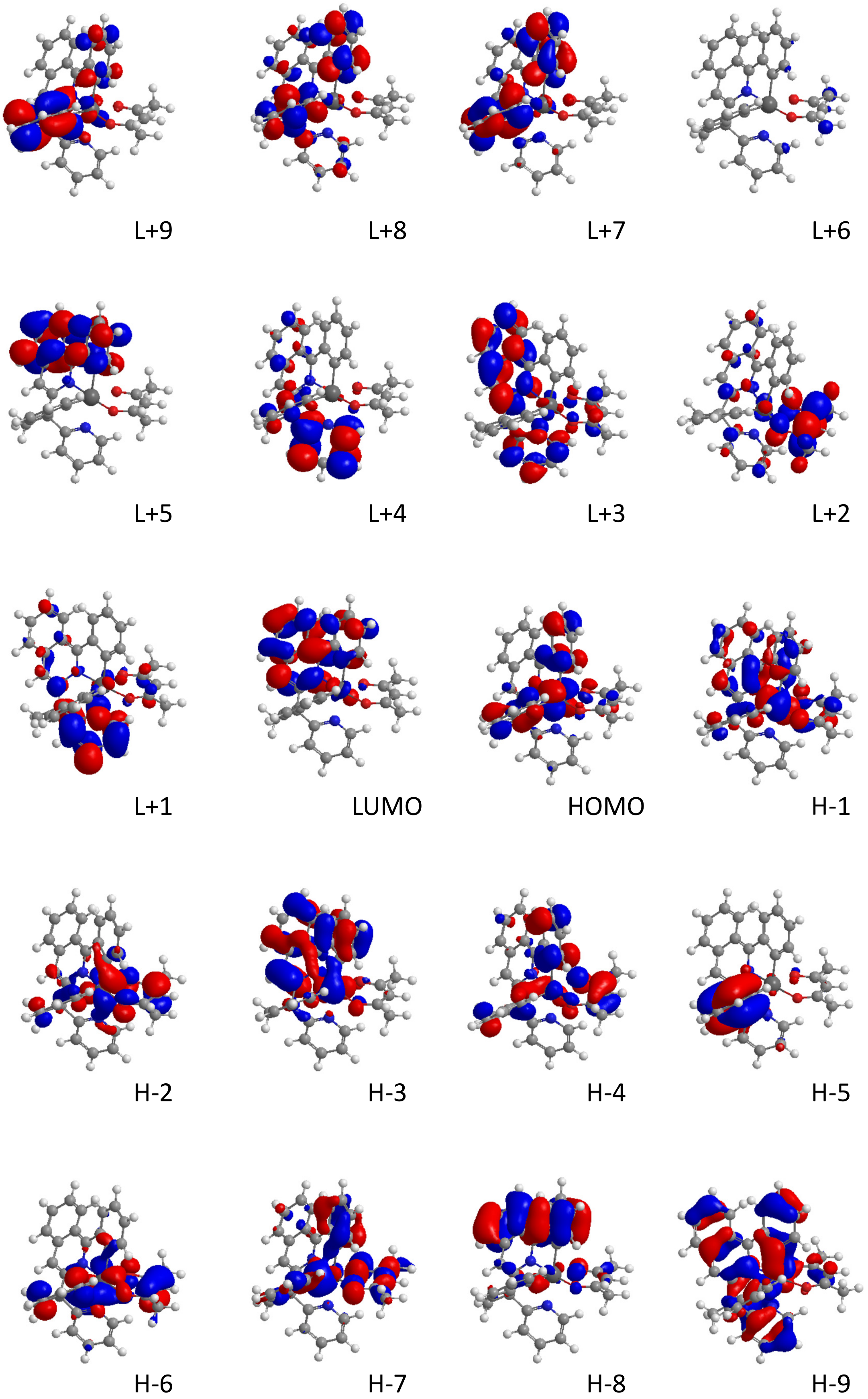

Figure S11. Frontier molecular orbitals of complex **5** (isovalue 0.03 au).

Table S21: Composition of the frontier orbitals of complex 6a.

MO	eV	Iridium	Ph	Py	isoqui	*Ph*-isoqui	C-Me	Ph-Py
**L+9**	0.5	4	22	3	7	37	0	28
**L+8**	0.46	4	53	4	8	28	0	3
**L+7**	0.20	97	0	0	1	1	0	1
**L+6**	-0.09	3	2	0	56	36	0	2
**L+5**	-0.44	3	9	76	10	1	1	1
**L+4**	-0.68	3	1	6	17	0	0	72
**L+3**	-0.79	2	1	18	57	1	1	20
**L+2**	-1.08	4	1	78	11	1	2	4
**L+1**	-1.18	4	1	2	3	0	0	90
**LUMO**	-1.77	3	0	1	73	19	0	4
**HOMO**	-4.90	50	9	5	4	15	0	16
**H-1**	-5.17	47	25	1	6	5	0	16
**H-2**	-5.31	45	6	1	14	18	0	16
**H-3**	-5.88	14	7	1	44	26	1	7
**H-4**	-6.05	6	2	1	4	2	0	85
**H-5**	-6.19	9	62	4	10	9	0	6
**H-6**	-6.25	11	62	1	3	15	0	8
**H-7**	-6.57	7	18	4	16	28	1	26
**H-8**	-6.71	18	8	6	15	13	0	40
**H-9**	-6.80	11	6	3	28	26	0	26

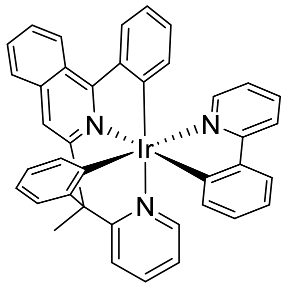

6a

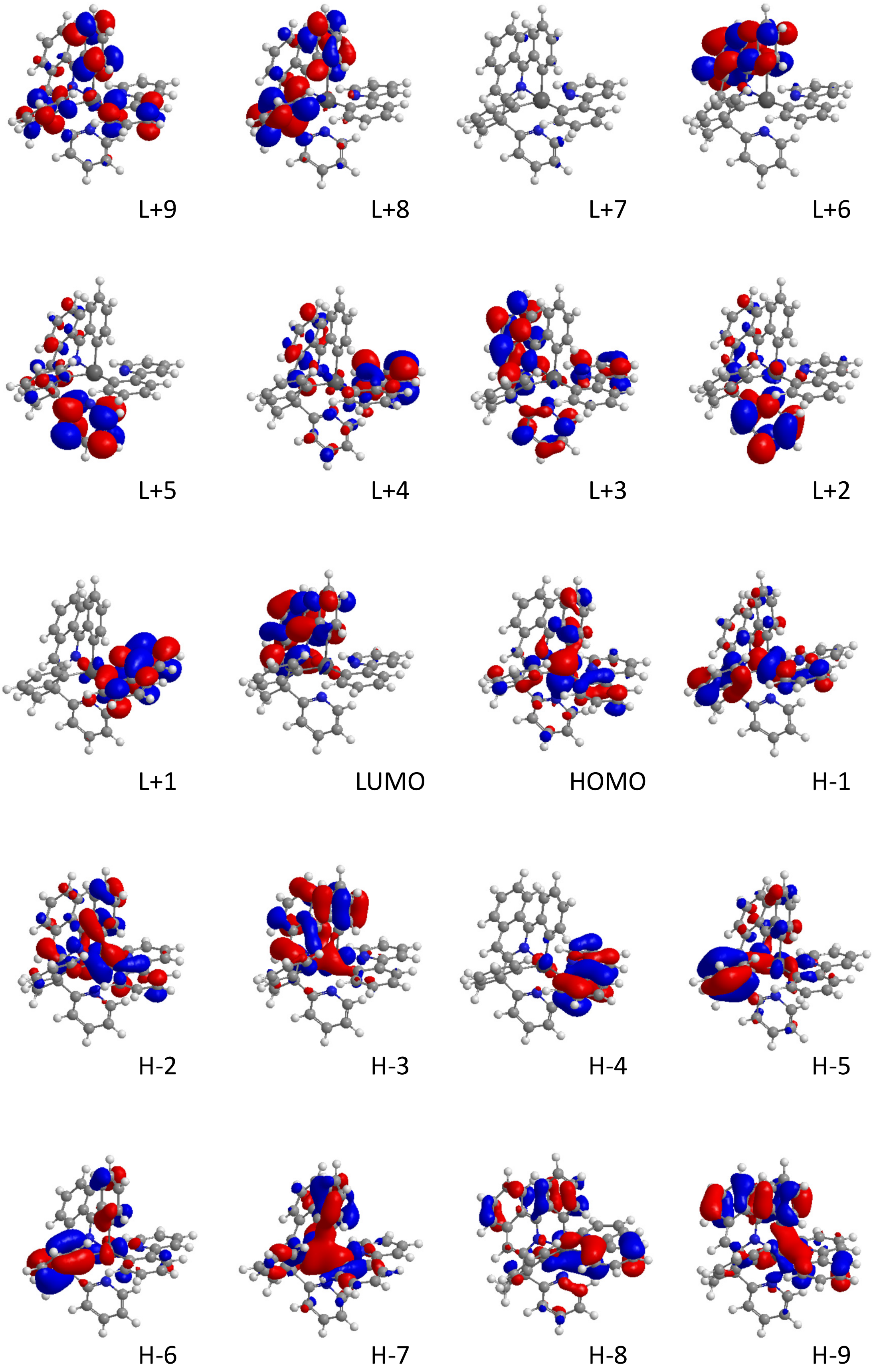

Figure S12. Frontier molecular orbitals of complex **6a** (isovalue 0.03 au).

Table S22: Composition of the frontier orbitals of complex 6b.

MO	eV	Iridium	Ph	Py	isoqui	*Ph*-isoqui	C-Me	Ph-Py
**L+9**	0.47	3	73	7	12	1	0	3
**L+8**	0.44	5	30	2	1	10	0	52
**L+7**	0.20	97	0	0	1	1	0	1
**L+6**	-0.06	3	2	0	57	36	0	2
**L+5**	-0.46	3	10	77	8	0	1	1
**L+4**	-0.72	3	1	4	26	0	1	65
**L+3**	-0.78	3	2	11	53	1	1	29
**L+2**	-1.03	3	2	85	4	1	2	4
**L+1**	-1.24	4	0	2	7	1	0	85
**LUMO**	-1.75	3	1	1	68	19	0	8
**HOMO**	-4.91	45	10	2	5	24	0	13
**H-1**	-5.28	43	20	1	22	8	0	6
**H-2**	-5.48	12	38	3	5	7	0	35
**H-3**	-5.72	46	4	3	15	13	0	19
**H-4**	-5.82	25	10	2	8	11	0	45
**H-5**	-6.03	13	5	2	26	18	0	35
**H-6**	-6.21	2	66	3	2	2	1	24
**H-7**	-6.28	7	26	2	4	24	0	37
**H-8**	-6.35	20	49	3	6	3	0	20
**H-9**	-6.72	2	1	0	47	47	0	1

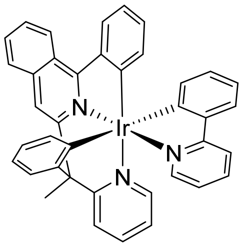

6b

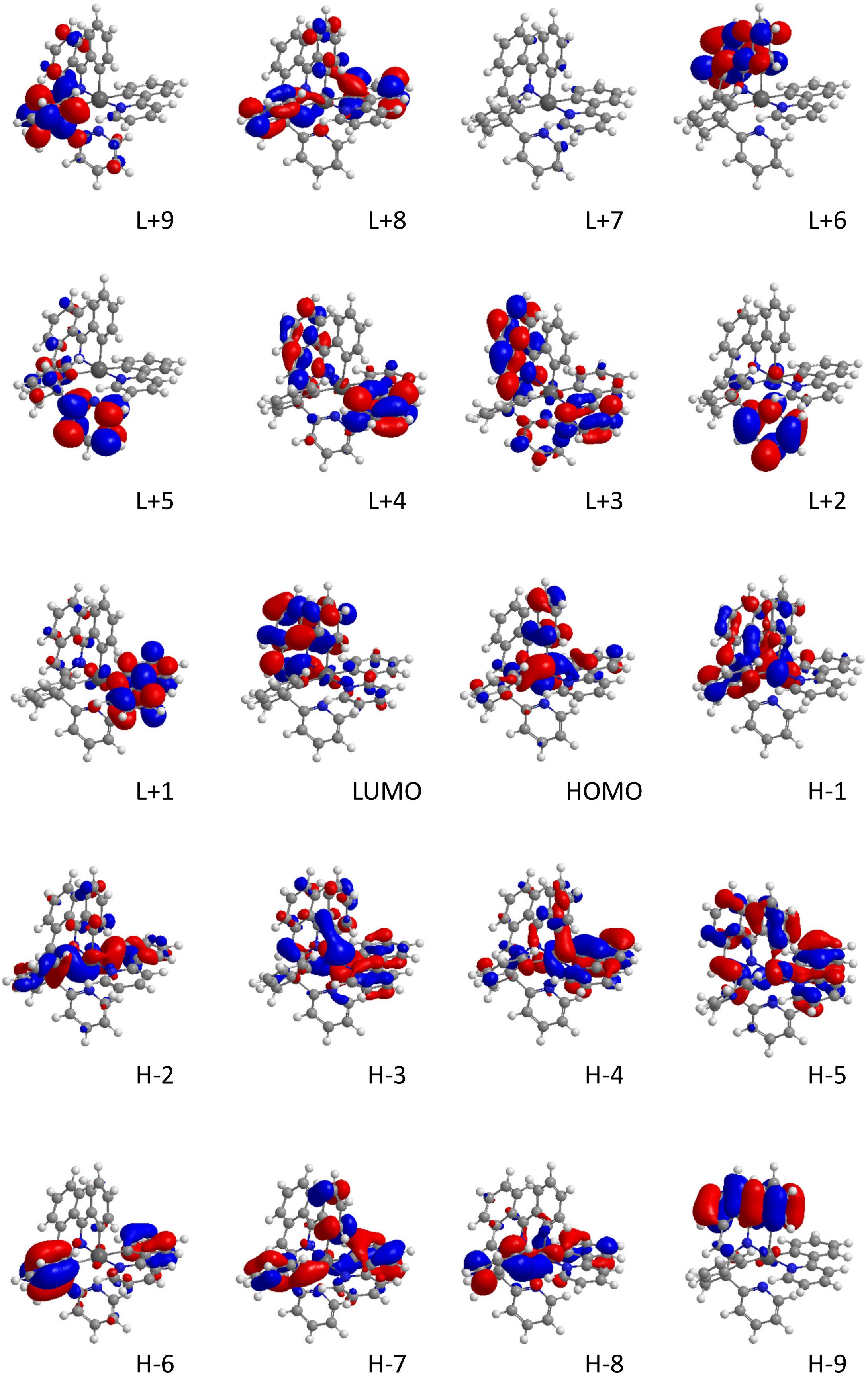

Figure S13. Frontier molecular orbitals of complex **6b** (isovalue 0.03 au).

Table S23: Composition of the frontier orbitals of complex 6c.

MO	eV	Iridium	Ph	Py	isoqui	*Ph-* isoqui	C-Me	Ph-Py
**L+9**	0.51	5	58	5	8	20	1	4
**L+8**	0.44	5	32	3	3	46	0	11
**L+7**	0.20	97	0	0	1	1	0	2
**L+6**	-0.09	3	1	2	59	32	0	2
**L+5**	-0.46	2	9	66	20	2	0	1
**L+4**	-0.67	4	0	7	13	0	0	76
**L+3**	-0.81	2	1	28	48	2	1	19
**L+2**	-1.08	5	1	47	9	0	1	37
**L+1**	-1.18	2	2	31	9	2	1	54
**LUMO**	-1.82	3	1	1	71	19	0	5
**HOMO**	-4.87	50	20	2	5	13	0	10
**H-1**	-5.22	26	21	0	1	20	1	31
**H-2**	-5.49	33	4	2	35	18	0	8
**H-3**	-5.69	29	13	1	6	34	0	17
**H-4**	-5.85	21	36	2	5	6	0	32
**H-5**	-6.04	28	13	2	25	13	0	19
**H-6**	-6.16	2	72	3	1	3	1	18
**H-7**	-6.25	9	22	2	3	8	0	55
**H-8**	-6.36	21	24	3	9	36	0	8
**H-9**	-6.72	2	0	1	46	48	0	2

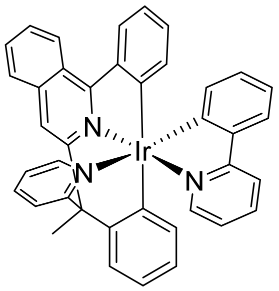

6c

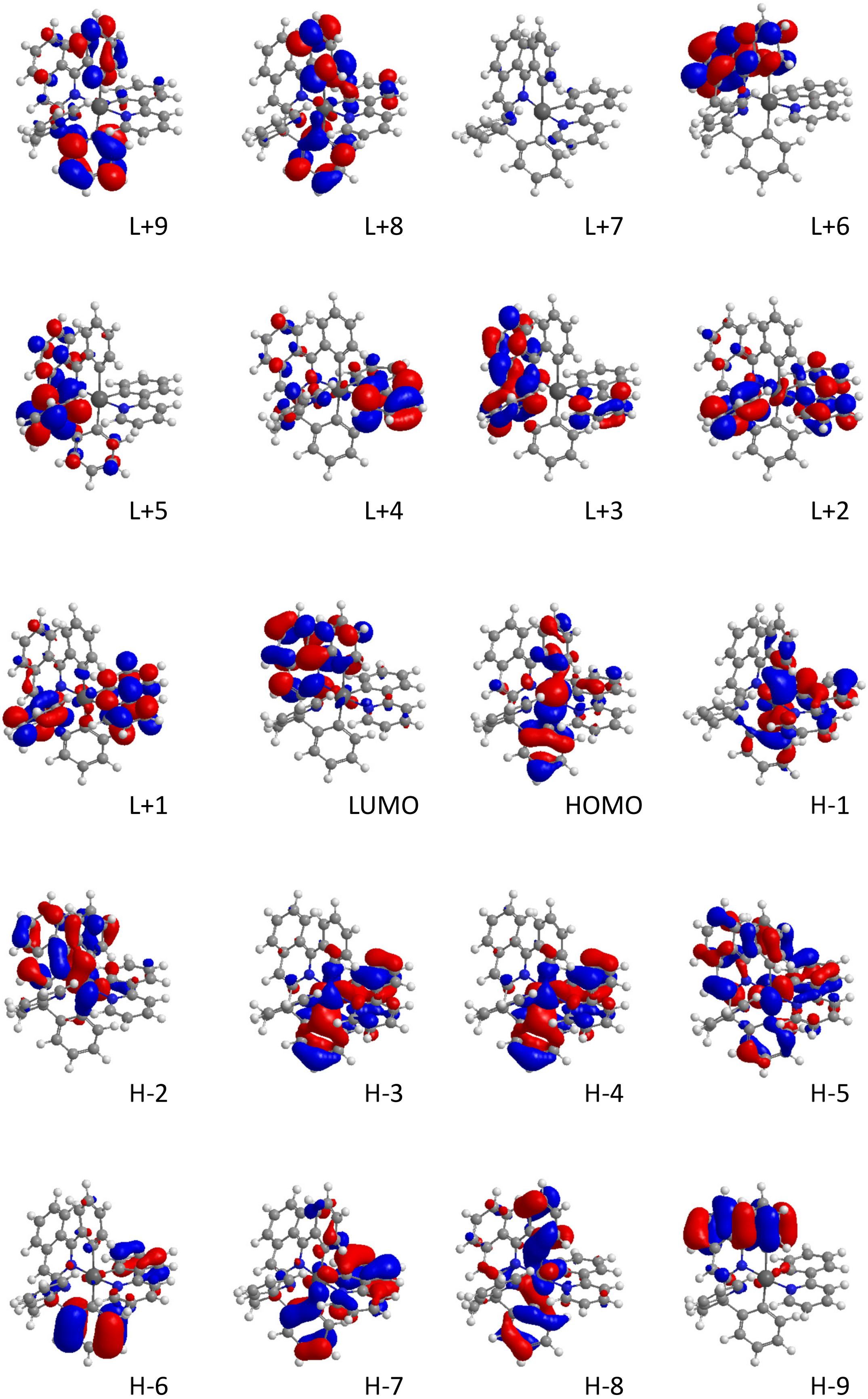

Figure S14. Frontier molecular orbitals of complex **6c** (isovalue 0.03 au).

Table S24: Composition of the frontier orbitals of complex 8.

MO	eV	Iridium	Ph	Py	isoqui	*Ph*-isoqui	C-Me	Ph-Py
**L+9**	-0.28	32	23	1	2	27	1	13
**L+8**	-0.50	3	81	1	4	8	1	1
**L+7**	-0.57	2	9	5	56	26	0	1
**L+6**	-0.88	12	28	24	10	7	1	17
**L+5**	-0.89	5	16	36	25	5	0	12
**L+4**	-1.30	5	12	8	7	2	1	66
**L+3**	-1.32	1	1	42	38	2	1	15
**L+2**	-1.69	4	2	47	11	0	1	35
**L+1**	-1.98	10	3	25	12	3	1	45
**LUMO**	-2.38	5	1	1	66	20	0	6
**HOMO**	-5.73	44	1	2	8	23	0	22
**H-1**	-6.17	21	2	1	38	15	0	23
**H-2**	-6.38	32	5	1	6	20	0	36
**H-3**	-6.59	39	15	2	8	13	0	22
**H-4**	-6.65	25	1	1	18	7	0	48
**H-5**	-6.80	19	2	3	16	17	0	42
**H-6**	-7.22	3	85	3	1	3	1	4
**H-7**	-7.25	2	11	0	38	47	1	1
**H-8**	-7.37	17	51	1	14	8	3	5
**H-9**	-7.70	8	3	39	27	5	1	18

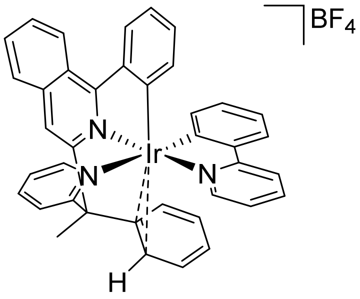

8

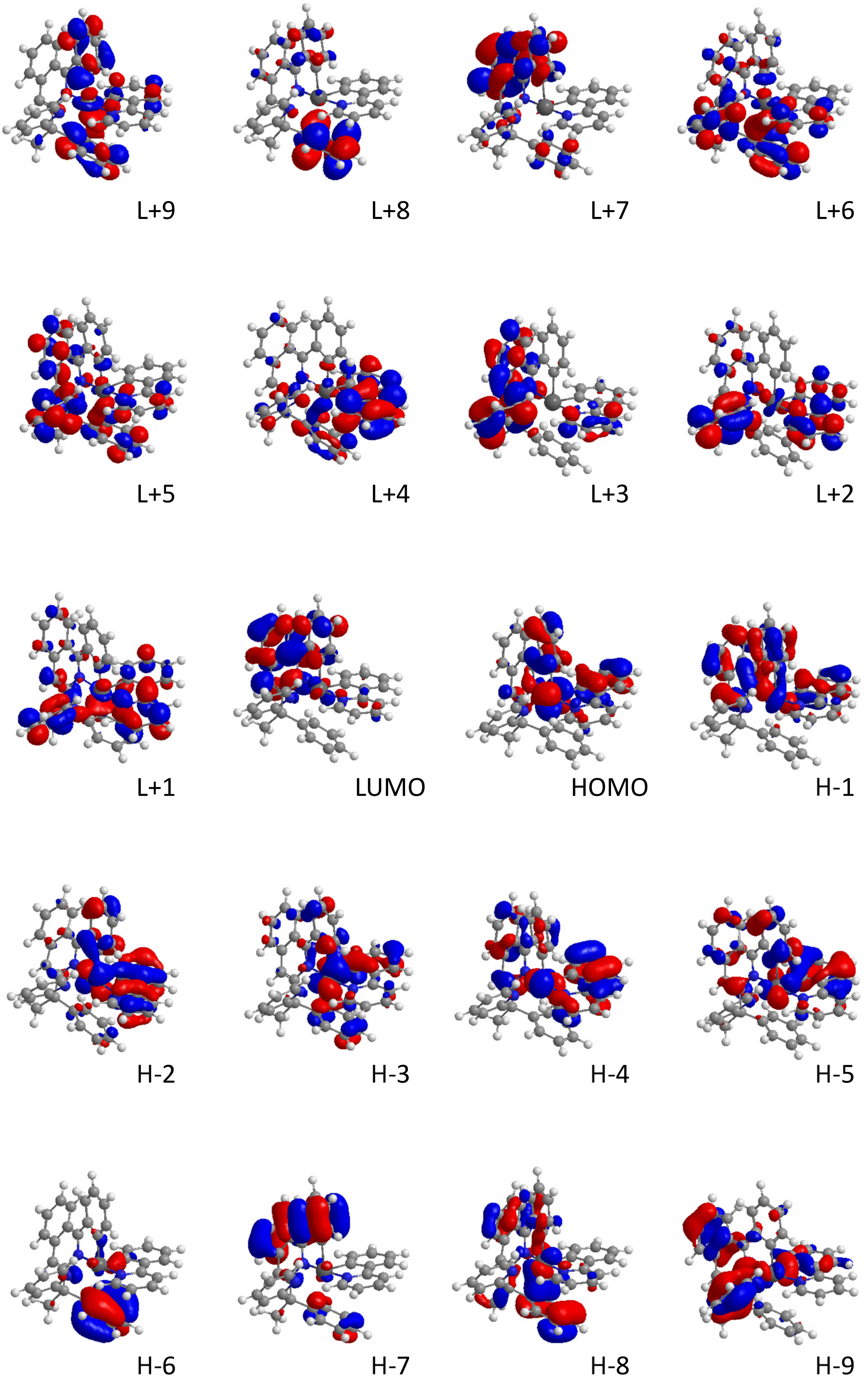

Figure S15. Frontier molecular orbitals of complex **8** (isovalue 0.03 au).

Table S25: Composition of the frontier orbitals of complex 9b.

MO	eV	Iridium	Ph	Py	isoqui	*Ph*-isoqui	C-Me	*p*-tolyl-Py
**L+9**	0.48	3	74	7	12	2	0	2
**L+8**	0.46	5	30	2	1	10	0	52
**L+7**	0.21	97	0	0	1	1	0	1
**L+6**	-0.06	3	1	0	57	36	0	2
**L+5**	-0.45	3	10	77	8	0	1	1
**L+4**	-0.70	3	1	2	16	0	0	77
**L+3**	-0.77	3	2	13	63	1	1	17
**L+2**	-1.02	3	2	84	4	1	2	4
**L+1**	-1.20	4	0	3	6	1	0	86
**LUMO**	-1.74	3	1	1	69	19	0	7
**HOMO**	-4.88	45	10	2	5	23	0	15
**H-1**	-5.27	44	21	0	21	8	0	6
**H-2**	-5.45	12	33	2	5	8	0	39
**H-3**	-5.67	25	8	2	15	13	0	37
**H-4**	-5.77	41	8	3	2	6	0	40
**H-5**	-5.96	18	3	1	29	16	0	33
**H-6**	-6.17	3	44	3	5	10	0	35
**H-7**	-6.24	6	46	3	4	21	0	20
**H-8**	-6.33	21	55	3	6	5	0	11
**H-9**	-6.71	2	2	1	47	47	0	1

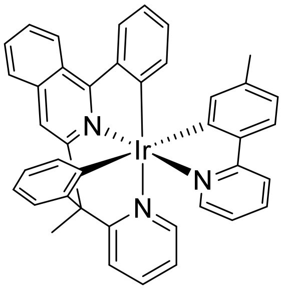

9b

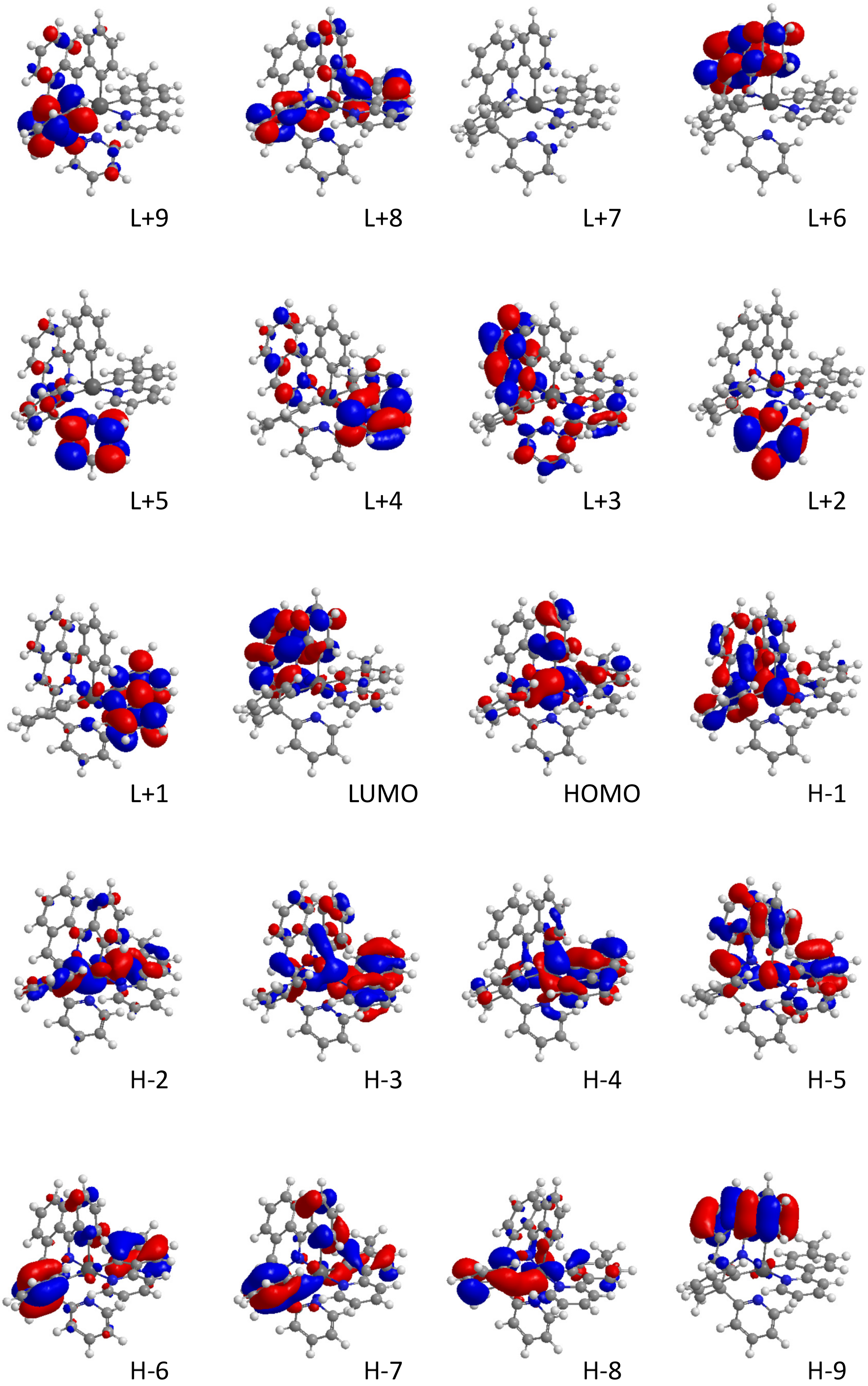

Figure S16. Frontier molecular orbitals of complex **9b** (isovalue 0.03 au).

Table S26: Composition of the frontier orbitals of complex 9c.

MO	eV	Iridium	Ph	Py	isoqui	*Ph*-isoqui	C-Me	*p*-tolyl-Py
**L+9**	0.52	5	57	5	7	21	1	4
**L+8**	0.44	5	32	3	3	45	0	12
**L+7**	0.20	97	0	0	1	1	0	1
**L+6**	-0.09	3	1	2	59	32	0	2
**L+5**	-0.46	2	9	67	19	2	0	1
**L+4**	-0.64	4	0	5	9	0	0	81
**L+3**	-0.80	2	1	29	52	2	1	14
**L+2**	-1.05	5	1	32	7	0	1	54
**L+1**	-1.15	2	2	46	10	1	1	37
**LUMO**	-1.81	3	1	1	72	19	0	4
**HOMO**	-4.85	50	19	2	4	13	0	13
**H-1**	-5.20	25	21	0	1	20	1	32
**H-2**	-5.47	31	4	2	35	18	0	9
**H-3**	-5.64	27	5	1	6	28	0	33
**H-4**	-5.78	20	42	2	1	10	0	26
**H-5**	-5.99	32	3	1	25	13	0	25
**H-6**	-6.14	2	62	2	3	5	0	25
**H-7**	-6.21	12	45	4	4	8	1	27
**H-8**	-6.34	20	24	3	9	36	0	8
**H-9**	-6.72	2	0	1	46	48	0	3

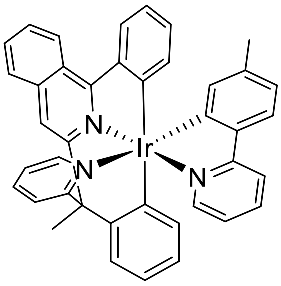

9c

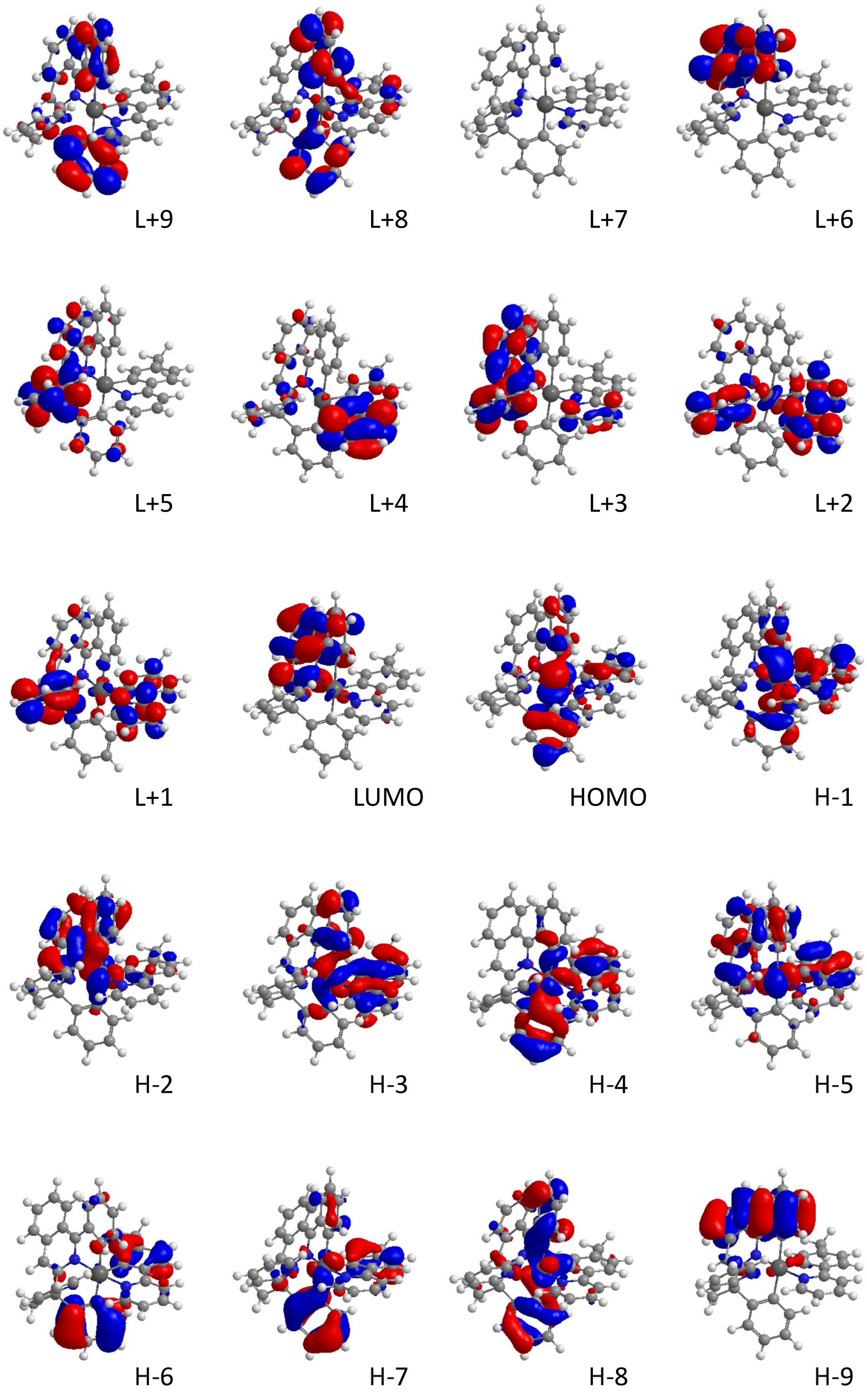

Figure S17. Frontier molecular orbitals of complex **9c** (isovalue 0.03 au).

Table S27: Composition of the frontier orbitals of complex 10.

MO	eV	Iridium	Ph	Py	isoqui	*Ph*-isoqui	C-Me	*p*-tolyl-Py
**L+9**	-0.26	35	22	1	2	27	1	12
**L+8**	-0.49	3	80	1	5	8	1	1
**L+7**	-0.57	2	10	4	56	26	0	1
**L+6**	-0.88	2	2	57	33	4	0	1
**L+5**	-0.90	15	41	4	1	7	2	31
**L+4**	-1.27	4	12	1	1	3	1	78
**L+3**	-1.32	1	2	48	44	1	1	2
**L+2**	-1.67	4	2	42	9	0	1	41
**L+1**	-1.96	10	4	31	13	3	1	39
**LUMO**	-2.37	6	1	1	67	20	0	5
**HOMO**	-5.69	42	0	2	7	22	0	27
**H-1**	-6.14	16	1	1	37	15	0	29
**H-2**	-6.29	25	3	1	4	16	0	51
**H-3**	-6.57	43	16	1	12	8	0	20
**H-4**	-6.58	31	2	1	13	12	0	40
**H-5**	-6.78	23	2	4	21	21	0	29
**H-6**	-7.23	3	86	3	1	2	1	4
**H-7**	-7.25	2	12	0	38	46	1	1
**H-8**	-7.35	16	51	1	14	9	3	5
**H-9**	-7.69	9	3	35	23	5	1	23

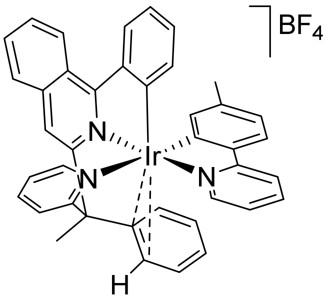

10

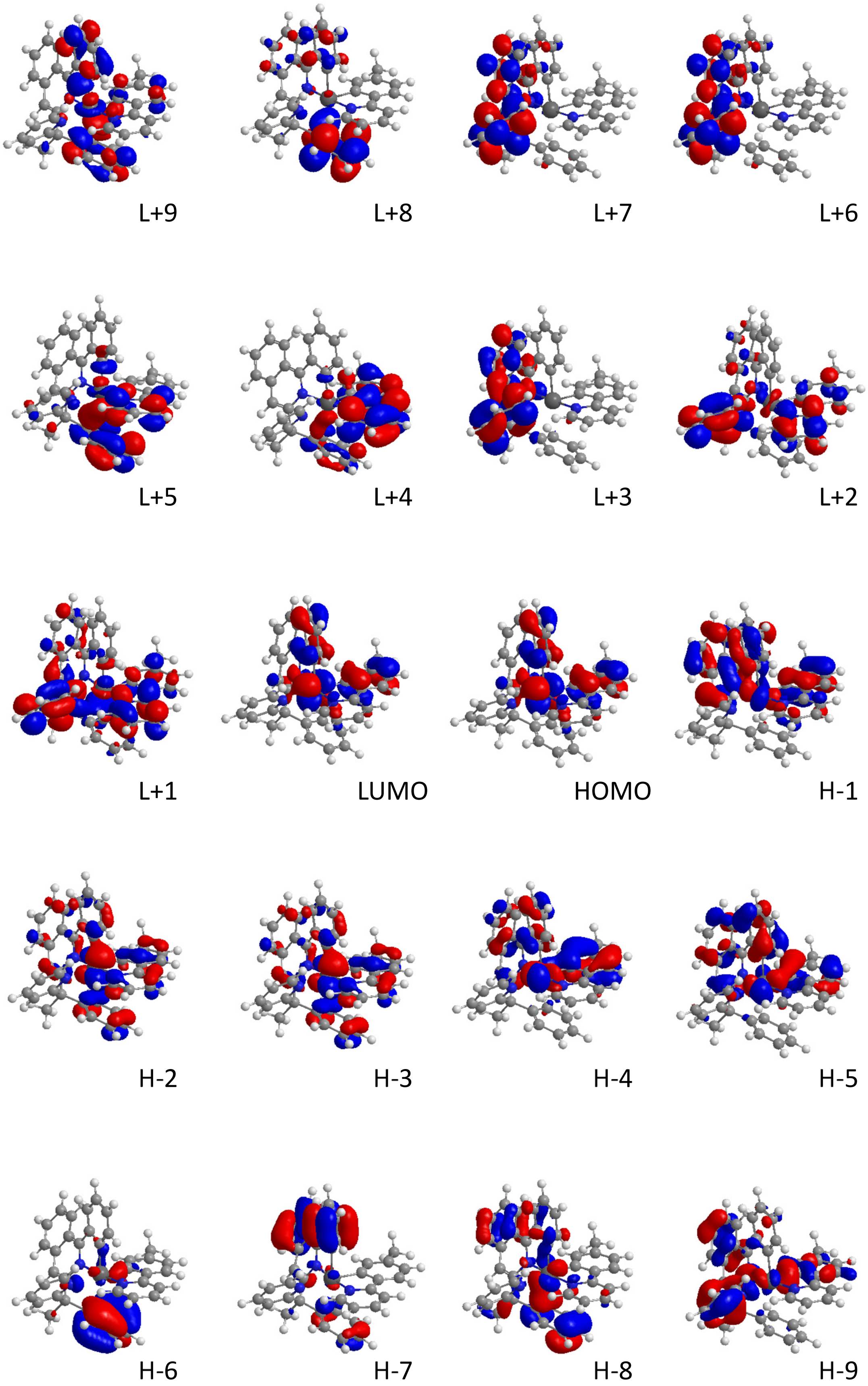

Figure S18. Frontier molecular orbitals of complex **10** (isovalue 0.03 au).

Natural transition orbitals (NTOs) and spin-density for complexes **3, 5, 6a-c, 8, 9b,c, and 10**

	**Electron**	**Hole**	**Spin density**

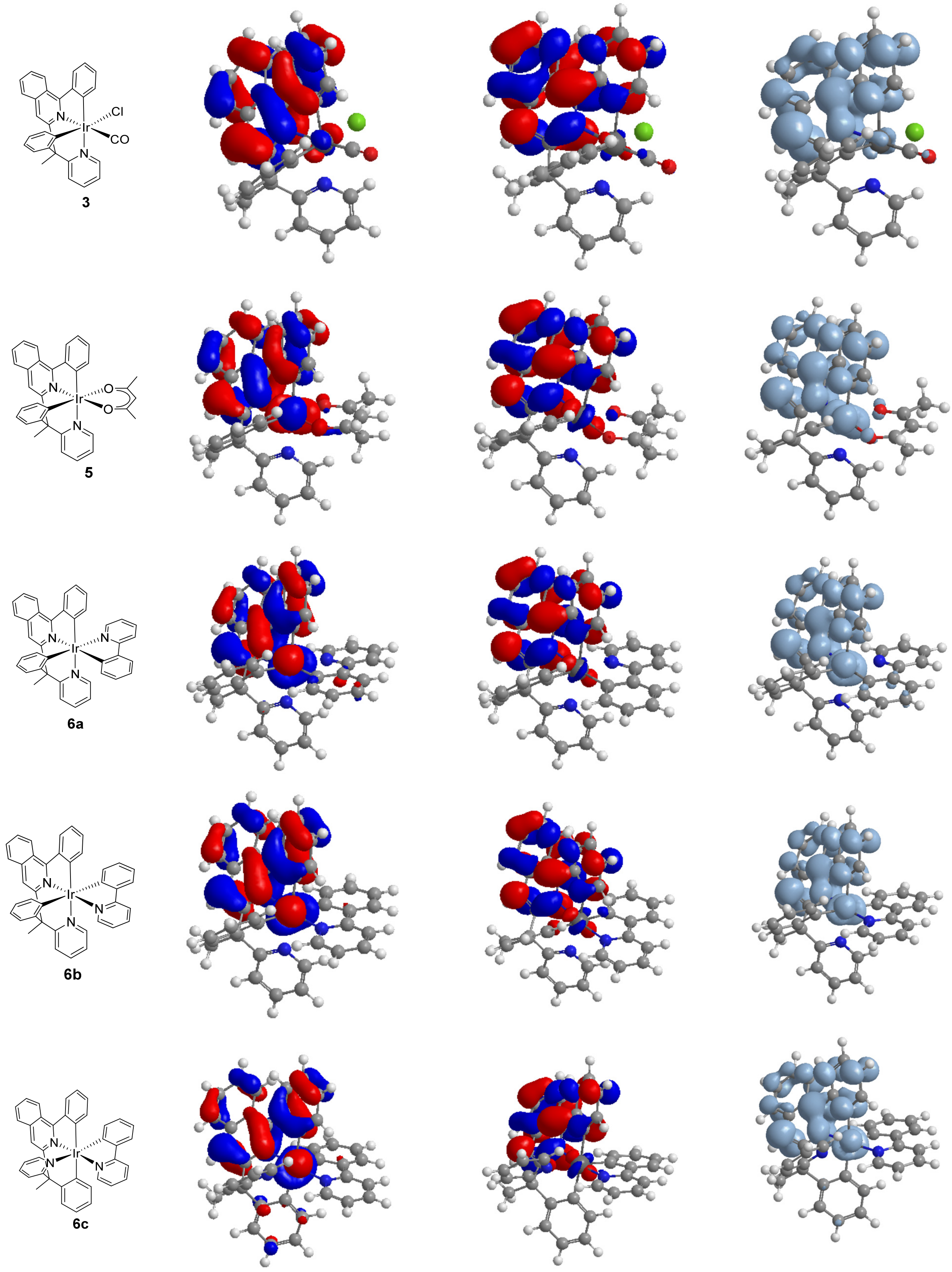

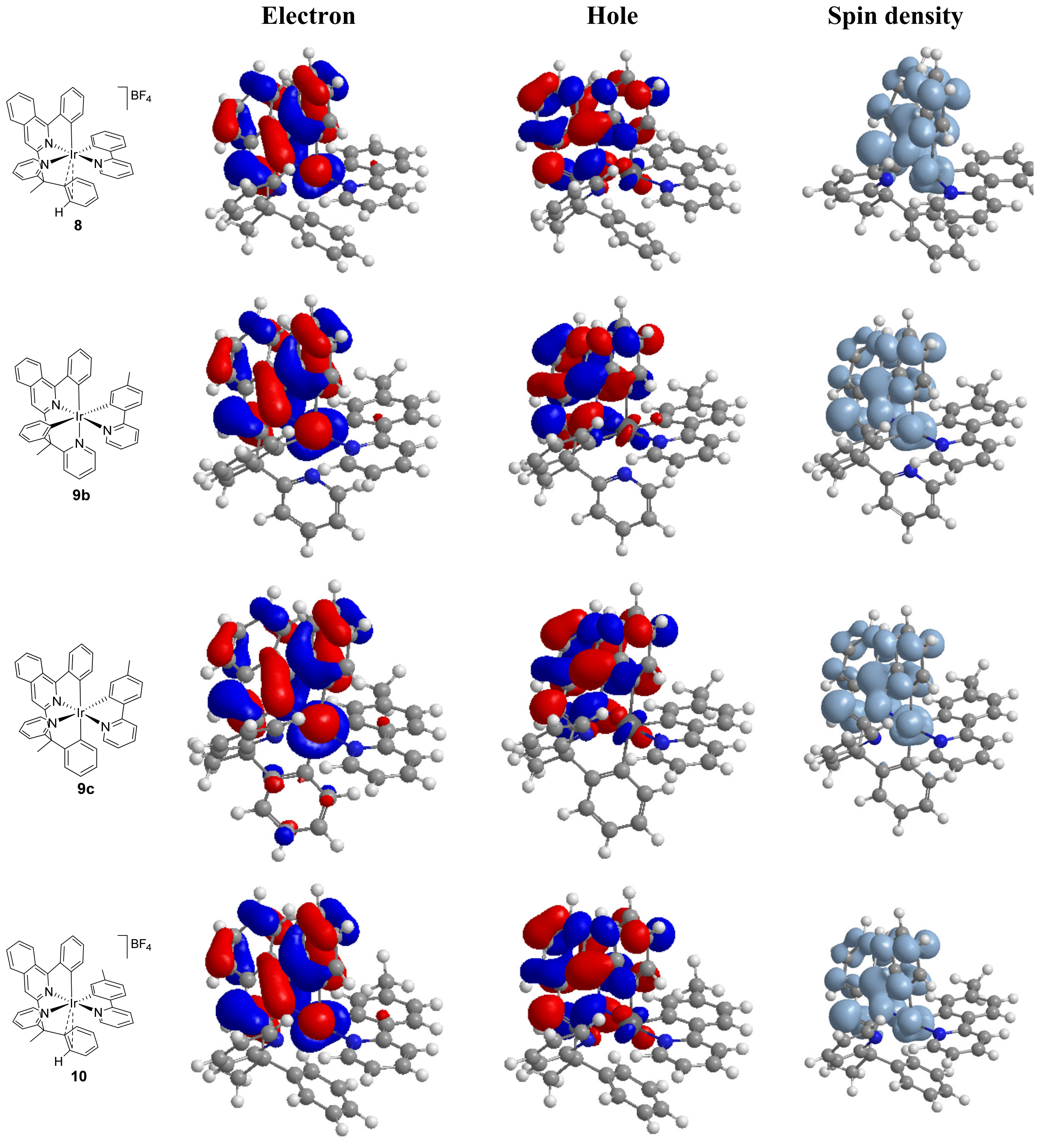

Figure S19. Natural transition orbitals (NTOs) (isovalue = 0.03) illustrating the nature of optically active triplet excited state T_1 and spin-density calculated (isovalue = 0.002) for the T_1 excited state) for complexes **3, 5, 6a-c, 8, 9b,c, and 10**.

Cyclic Voltammograms of Complexes 3, 5, 6, 9, and 8-10

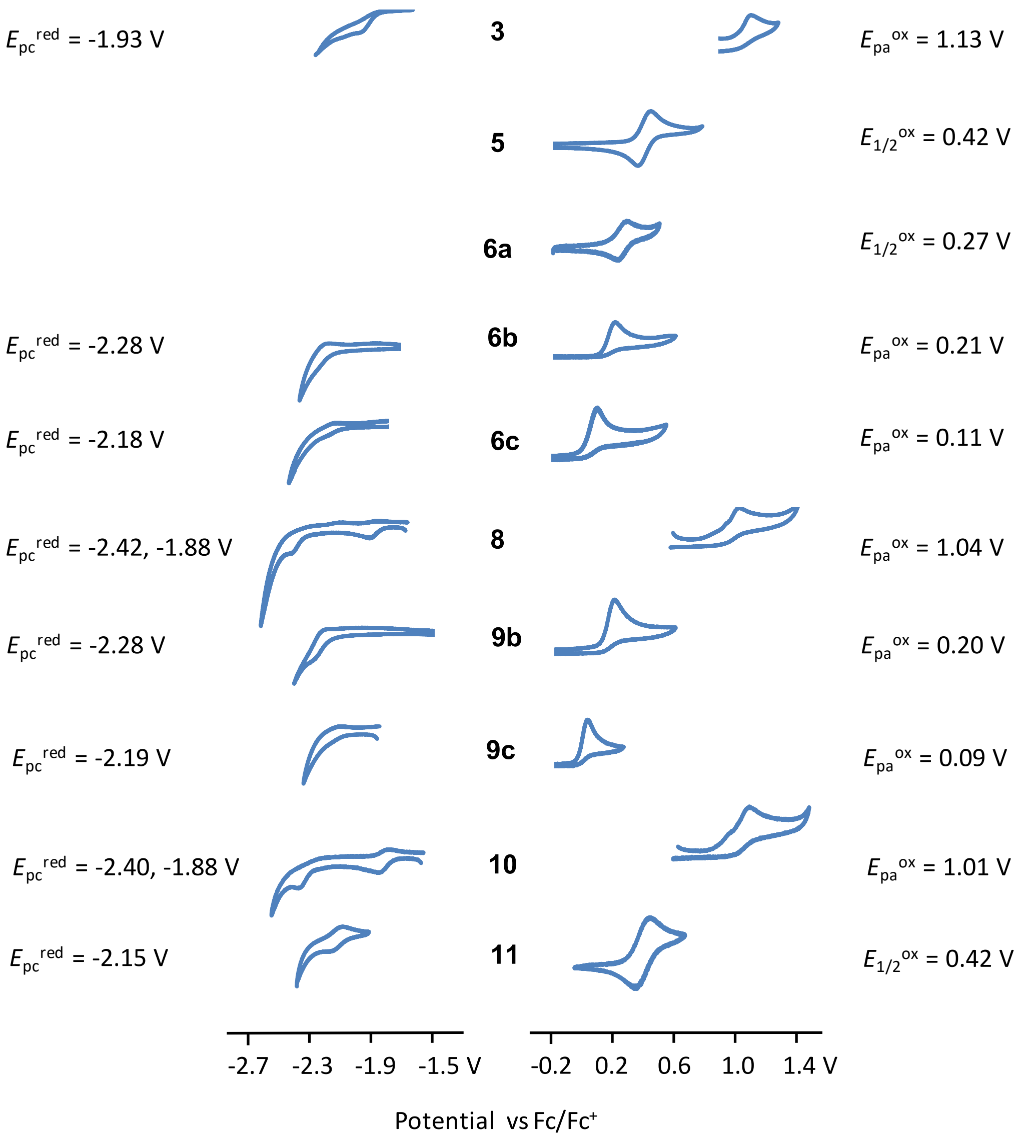

Figure S20. Cyclic voltammograms in 1:1 acetonitrile/dichlorometane (5x10^{-4} M, **3**) or acetonitrile (10^{-3} M, **5**, **6**, and **8-11**) solutions with Bu_4NPF_6 (0.1 M) as supporting electrolyte at a scan rate of 100 mV s^{-1}. The potentials are referenced to the ferrocene/ferrocenium (Fc/Fc$^+$) couple.

.

Normalized Emission Spectra of Complexes 3, 5, 6a-c, 8, 9b,c, and 10.

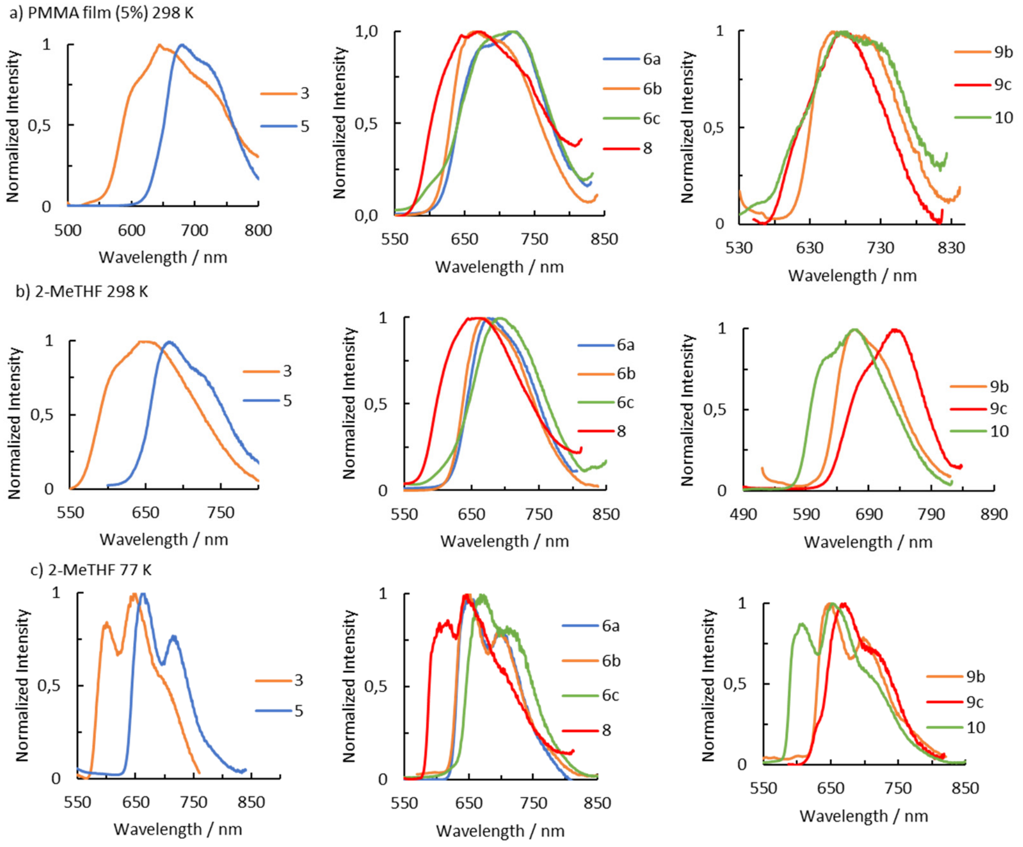

Figure S21. Normalized emission spectra of compounds **3, 5, 6a-c, 8a ,9b,c, and 10** in 2-MeTHF. a) PMMA film (5% wt) at 298 K. b) 2-MeTHF at 298 K. c) 2-MeTHF at 77 K.

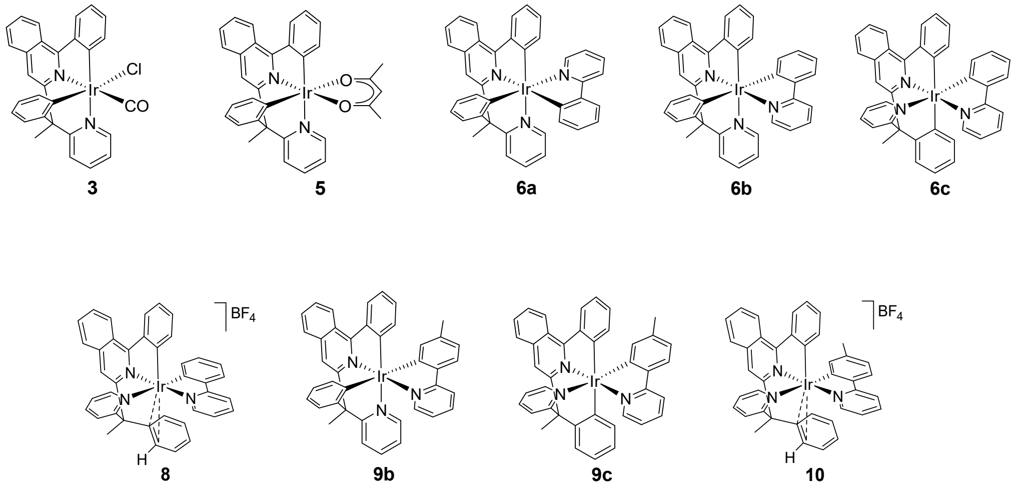

Normalized Excitation and Emission Spectra of Complexes 3, 5, 6a-c, 8, 9b,c, and 10.

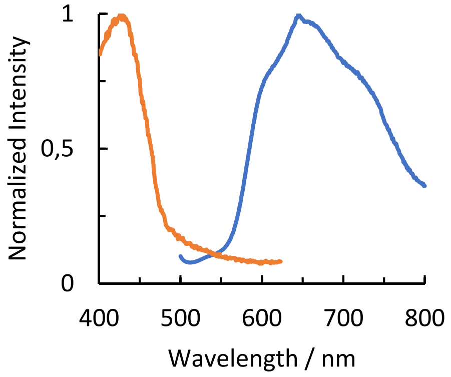

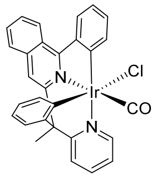

3

Figure S22. Normalized excitation (orange line) and emission (blue line) spectra of Ir(κ^4-*cis*-*C,C′*-*cis*-*N,N′*-MeL)Cl(CO) (**3**) in PMMA film (5 wt%) at 298 K.

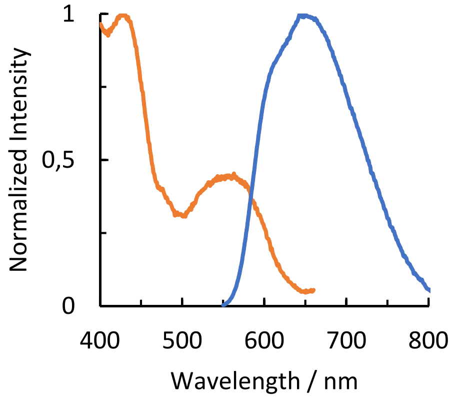

Figure S23. Normalized excitation (orange line) and emission (blue line) spectra of Ir(κ^4-*cis*-*C,C′*-*cis*-*N,N′*-MeL)Cl(CO) (**3**) in a 1.0 x 10^{-5} M solution in 2-MeTHF at 298 K.

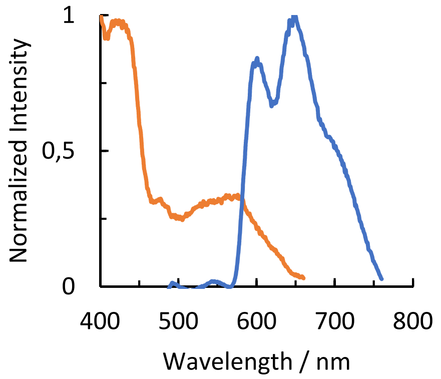

Figure S24. Normalized excitation (orange line) and emission (blue line) spectra of Ir(κ^4-*cis*-*C,C′*-*cis*-*N,N′*-MeL)Cl(CO) (**3**) in a 1.0 x 10^{-5} M solution in 2-MeTHF at 77 K.

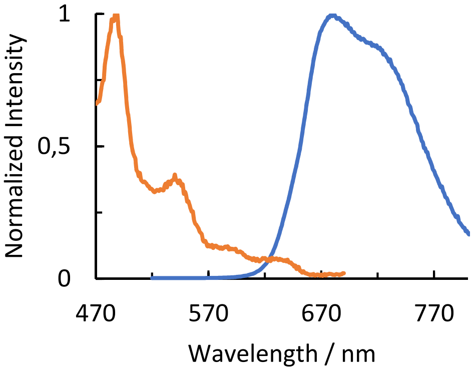

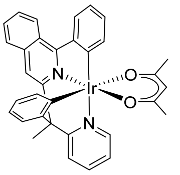

5

Figure S25. Normalized excitation (orange line) and emission (blue line) spectra of Ir(κ^4-*cis*-*C,C*′-*cis*-*N,N*′-MeL)(acac) (**5**) in PMMA film (5 wt%) at 298 K.

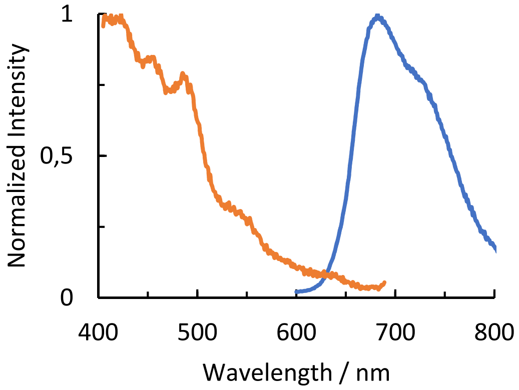

Figure S25. Normalized excitation (orange line) and emission (blue line) spectra of Ir(κ^4-*cis*-*C,C*′-*cis*-*N,N*′-MeL)(acac) (**5**) in a 1.0 x 10^{-5} M solution in 2-MeTHF at 298 K.

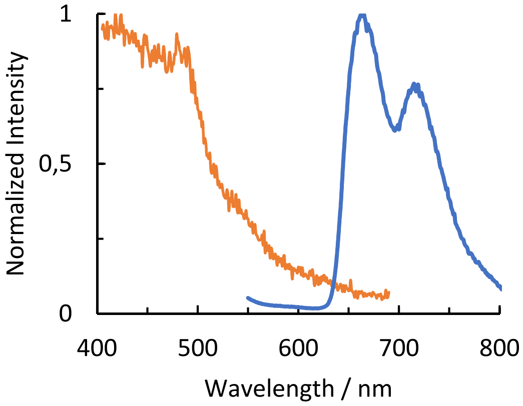

Figure S27. Normalized excitation (orange line) and emission (blue line) spectra of Ir(κ^4-*cis*-*C,C*′-*cis*-*N,N*′-MeL)(acac) (**5**) in a 1.0 x 10^{-5} M solution in 2-MeTHF at 77 K.

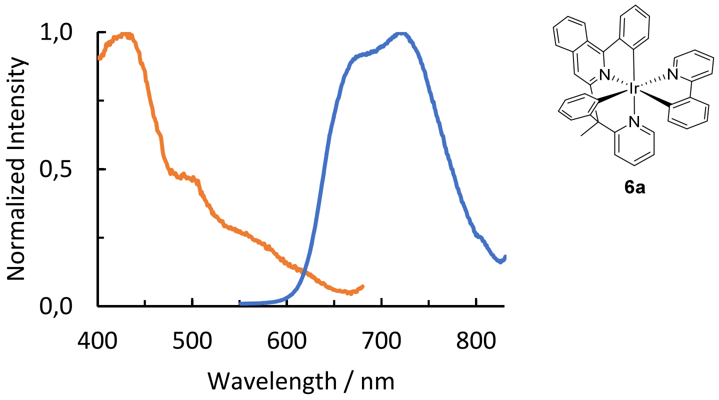

Figure S28. Normalized excitation (orange line) and emission (blue line) spectra of *fac*-Ir(κ^4-*cis*-*C,C′*-*cis*-*N,N′*-MeL){κ^2-*C,N*-(C_6H_4-py) (**6a**) in PMMA film (5 wt%) at 298 K.

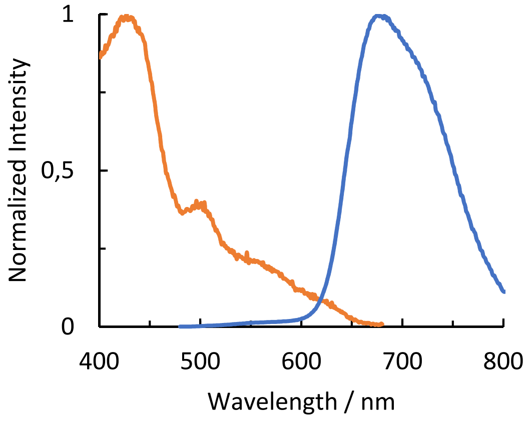

Figure S29. Normalized excitation (orange line) and emission (blue line) spectra of *fac*-Ir(κ^4-*cis*-*C,C′*-*cis*-*N,N′*-MeL){κ^2-*C,N*-(C_6H_4-py) (**6a**) in a 1.0 x 10^{-5} M solution in 2-MeTHF at 298 K.

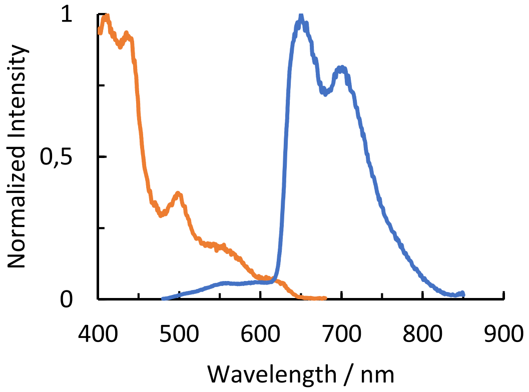

Figure S30. Normalized excitation (orange line) and emission (blue line) spectra of *fac*-Ir(κ^4-*cis*-*C,C′*-*cis*-*N,N′*-MeL){κ^2-*C,N*-(C_6H_4-py) (**6a**) in a 1.0 x 10^{-5} M solution in 2-MeTHF at 77 K.

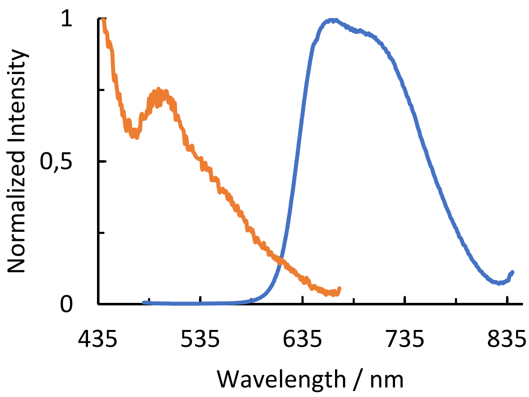

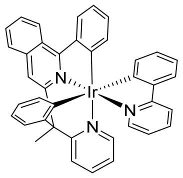

6b

Figure S31. Normalized excitation (orange line) and emission (blue line) spectra of *mer*-Ir(κ^4-*cis*-*C,C′*-*cis*-*N,N′*-MeL){κ^2-*C,N*-(C_6H_4-py) (**6b**) in PMMA film (5 wt%) at 298 K.

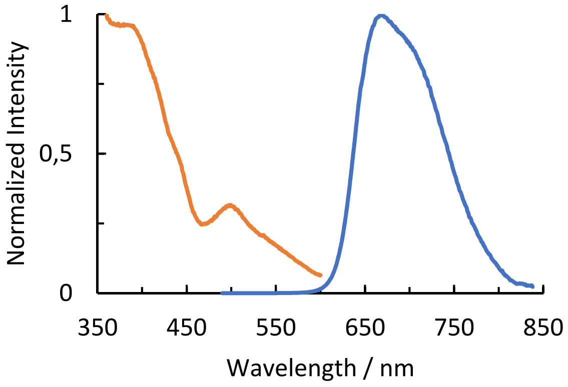

Figure S32. Normalized excitation (orange line) and emission (blue line) spectra of *mer*-Ir(κ^4-*cis*-*C,C′*-*cis*-*N,N′*-MeL){κ^2-*C,N*-(C_6H_4-py) (**6b**) in a 1.0 x 10^{-5} M solution in 2-MeTHF at 298 K.

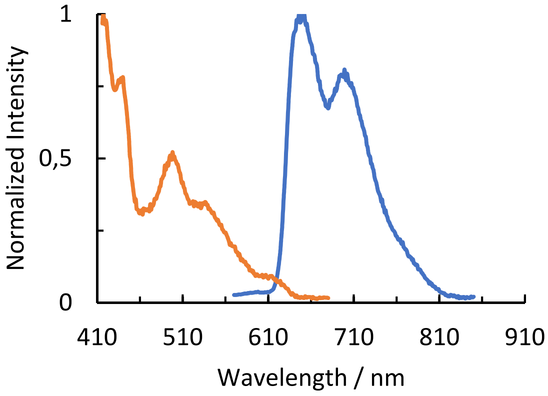

Figure S33. Normalized excitation (orange line) and emission (blue line) spectra of *mer*-Ir(κ^4-*cis*-*C,C′*-*cis*-*N,N′*-MeL){κ^2-*C,N*-(C_6H_4-py) (**6b**) in a 1.0 x 10^{-5} M solution in 2-MeTHF at 77 K.

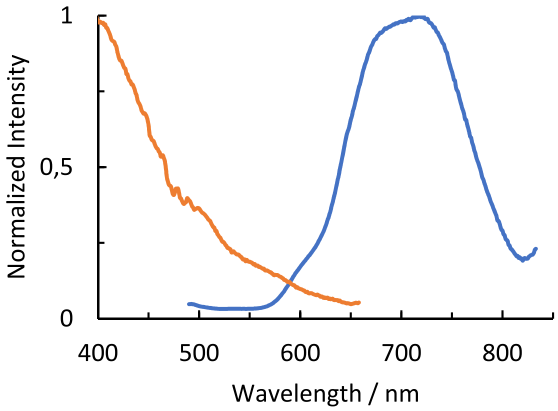

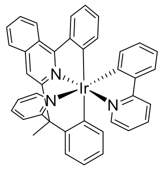

6c

Figure S34. Normalized excitation (orange line) and emission (blue line) spectra of *mer*-Ir(κ^4-*trans*-*C,C*′-*cis*-*N,N*′-MeL){κ^2-*C,N*-(C_6H_4-py) (**6c**) in PMMA film (5 wt%) at 298 K.

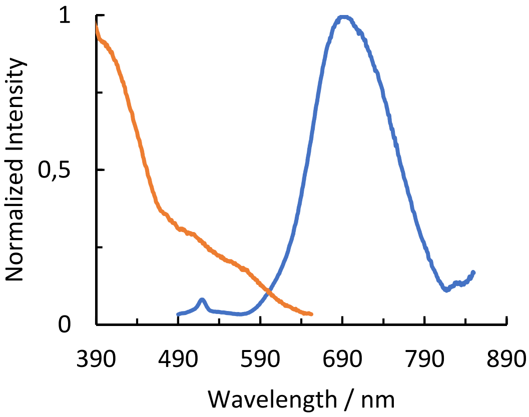

Figure S35. Normalized excitation (orange line) and emission (blue line) spectra of *mer*-Ir(κ^4-*trans*-*C,C*′-*cis*-*N,N*′-MeL){κ^2-*C,N*-(C_6H_4-py) (**6c**) in a 1.0 x 10^{-5} M solution in 2-MeTHF at 298 K.

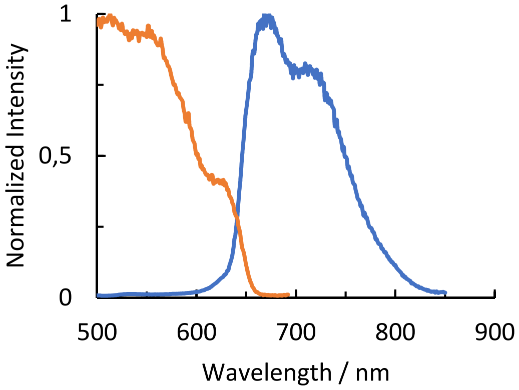

Figure S36. Normalized excitation (orange line) and emission (blue line) spectra of *mer*-Ir(κ^4-*trans*-*C,C*′-*cis*-*N,N*′-MeL){κ^2-*C,N*-(C_6H_4-py) (**6c**) in a 1.0 x 10^{-5} M solution in 2-MeTHF at 77 K.

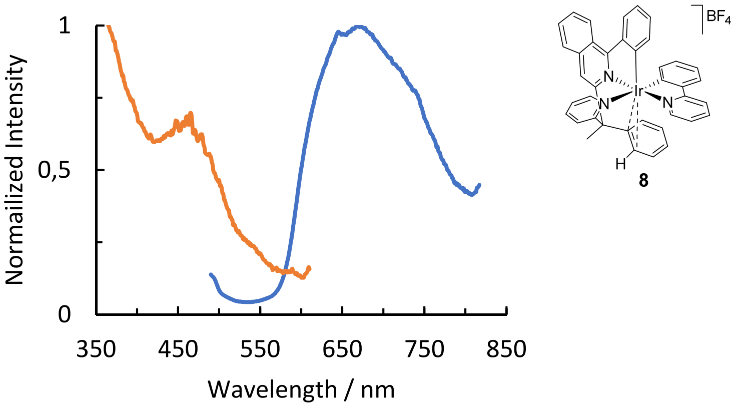

Figure S37. Normalized excitation (orange line) and emission (blue line) spectra of [Ir(κ^3-*C,N,N'*; η^2-*CC)*-MeHL)(κ^2-*C,N*-C_6H_4-py)]BF_4 (**8**) in PMMA film (5 wt%) at 298 K.

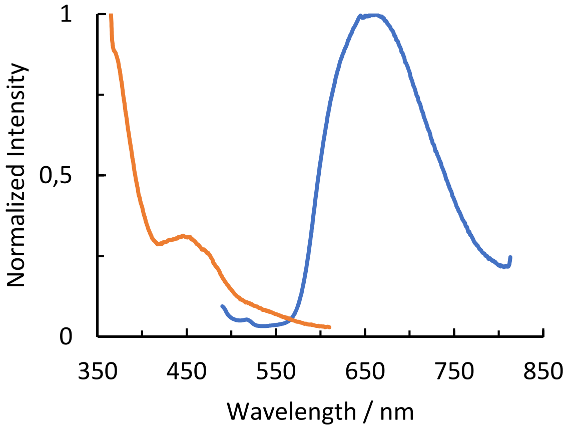

Figure S38. Normalized excitation (orange line) and emission (blue line) spectra of [Ir(κ^3-*C,N,N'*; η^2-*CC)*-MeHL)(κ^2-*C,N*-C_6H_4-py)]BF_4 (**8**) in a 1.0 x 10^{-5} M solution in 2-MeTHF at 298 K.

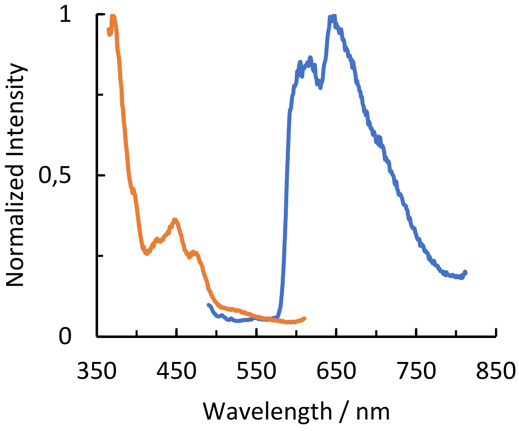

Figure S39. Normalized excitation (orange line) and emission (blue line) spectra of [Ir(κ^3-*C,N,N'*; η^2-*CC)*-MeHL)(κ^2-*C,N*-C_6H_4-py)]BF_4 (**8**) in a 1.0 x 10^{-5} M solution in 2-MeTHF at 77 K.

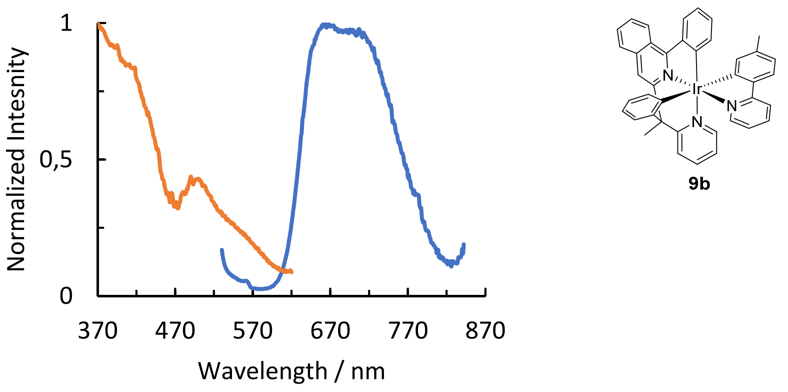

Figure S40. Normalized excitation (orange line) and emission (blue line) spectra of *mer*-Ir(κ^4-*cis*-*C,C′*-*cis*-*N,N′*-MeL){κ^2-*C,N*-(C_6H_3Me-py) (**9b**) in PMMA film (5 wt%) at 298 K.

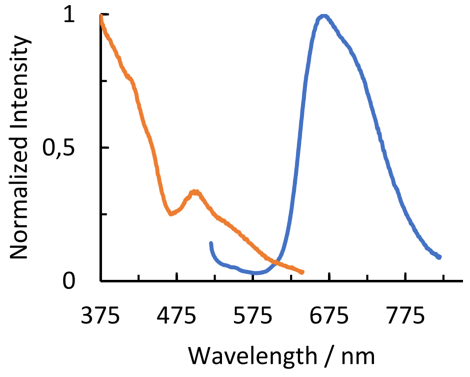

Figure S41. Normalized excitation (orange line) and emission (blue line) spectra of *mer*-Ir(κ^4-*cis*-*C,C′*-*cis*-*N,N′*-MeL){κ^2-*C,N*-(C_6H_3Me-py) (**9b**) in a 1.0 x 10^{-5} M solution in 2-MeTHF at 298 K.

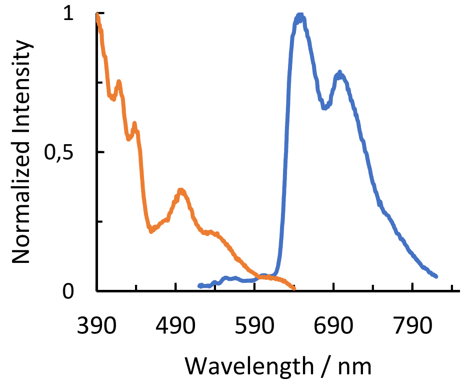

Figure S42. Normalized excitation (orange line) and emission (blue line) spectra of *mer*-Ir(κ^4-*cis*-*C,C′*-*cis*-*N,N′*-MeL){κ^2-*C,N*-(C_6H_3Me-py) (**9b**) in a 1.0 x 10^{-5} M solution in 2-MeTHF at 77 K.

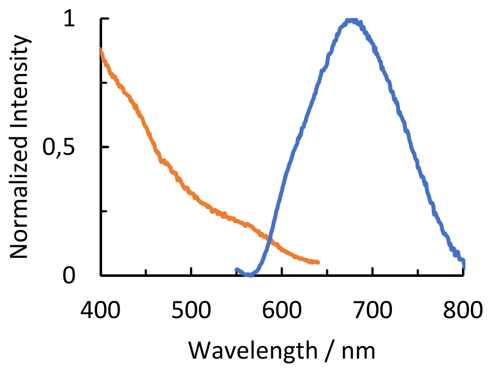

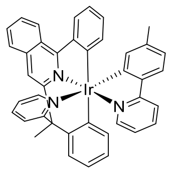

9c

Figure S43. Normalized excitation (orange line) and emission (blue line) spectra of *mer*-Ir(κ^4-*trans*-*C,C′*-*cis*-*N,N′*-MeL){κ^2-*C,N*-(C_6H_3Me-py) (**9c**) in PMMA film (5 wt%) at 298 K.

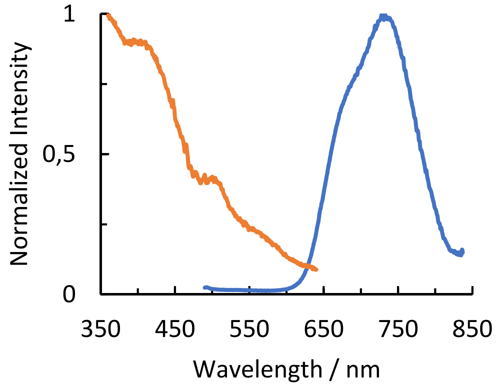

Figure S44. Normalized excitation (orange line) and emission (blue line) spectra of *mer*-Ir(κ^4-*trans*-*C,C′*-*cis*-*N,N′*-MeL){κ^2-*C,N*-(C_6H_3Me-py) (**9c**) in a 1.0 x 10^{-5} M solution in 2-MeTHF at 298 K.

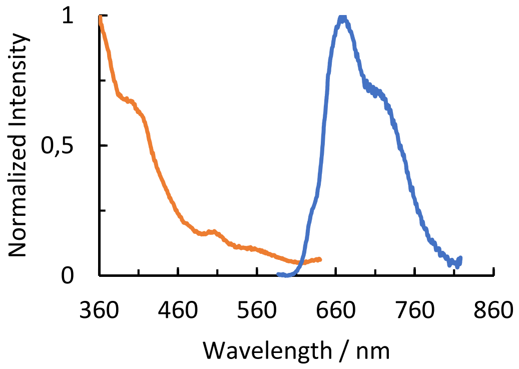

Figure S45. Normalized excitation (orange line) and emission (blue line) spectra of *mer*-Ir(κ^4-*trans*-*C,C′*-*cis*-*N,N′*-MeL){κ^2-*C,N*-(C_6H_3Me-py) (**9c**) in a 1.0 x 10^{-5} M solution in 2-MeTHF at 77 K

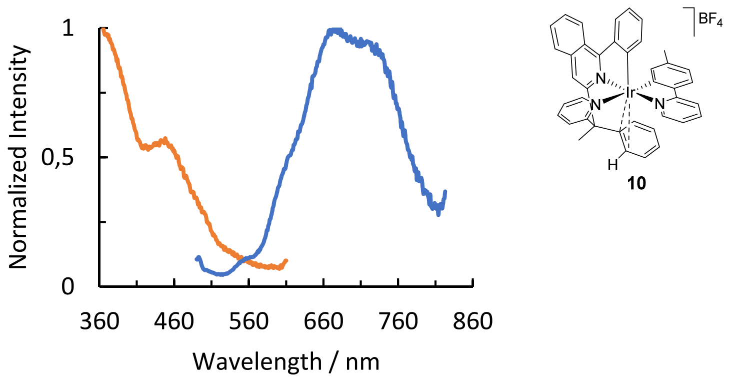

Figure S46. Normalized excitation (orange line) and emission (blue line) spectra of of [Ir(κ^3-*C,N,N'*; η^2-*CC)*-MeHL)(κ^2-*C,N*-C_6H_3Me-py)]BF_4 (**10**) in PMMA film (5 wt%) at 298 K.

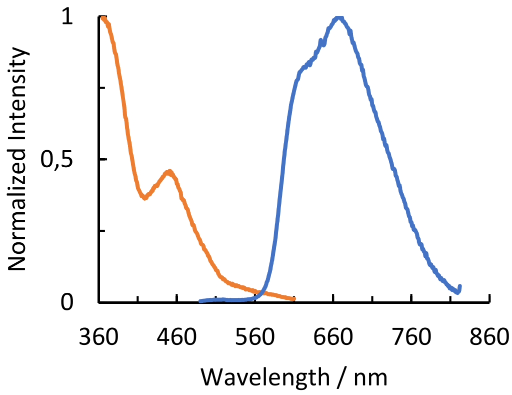

Figure S47. Normalized excitation (orange line) and emission (blue line) spectra of [Ir(κ^3-*C,N,N'*; η^2-*CC)*-MeHL)(κ^2-*C,N*-C_6H_3Me-py)]BF_4 (**10**) in a 1.0 x 10^{-5} M solution in 2-MeTHF at 298 K.

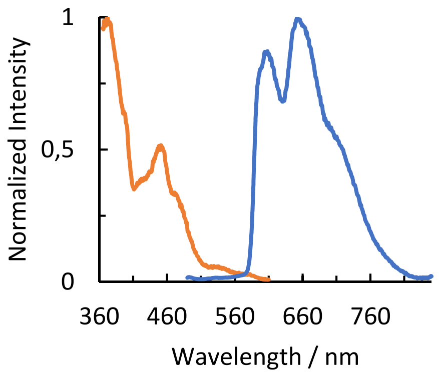

Figure S48. Normalized excitation (orange line) and emission (blue line) spectra of [Ir(κ^3-*C,N,N'*; η^2-*CC)*-MeHL)(κ^2-*C,N*-C_6H_3Me-py)]BF_4 (**10**) in a 1.0 x 10^{-5} M solution in 2-MeTHF at 77 K.

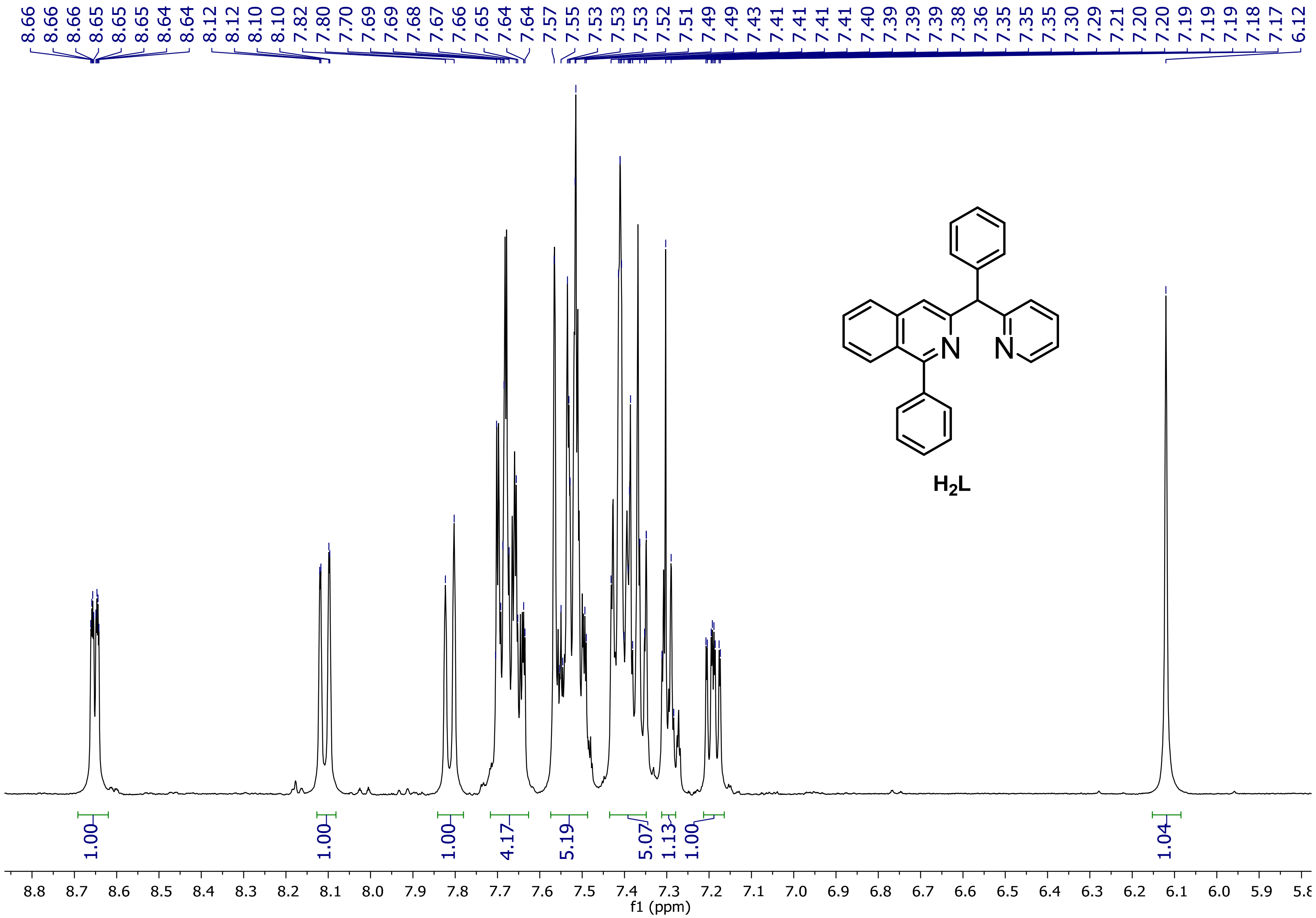

Figure S49. ^{1}H NMR (400 MHz, $CDCl_3$, 298 K) spectrum of H_2L.

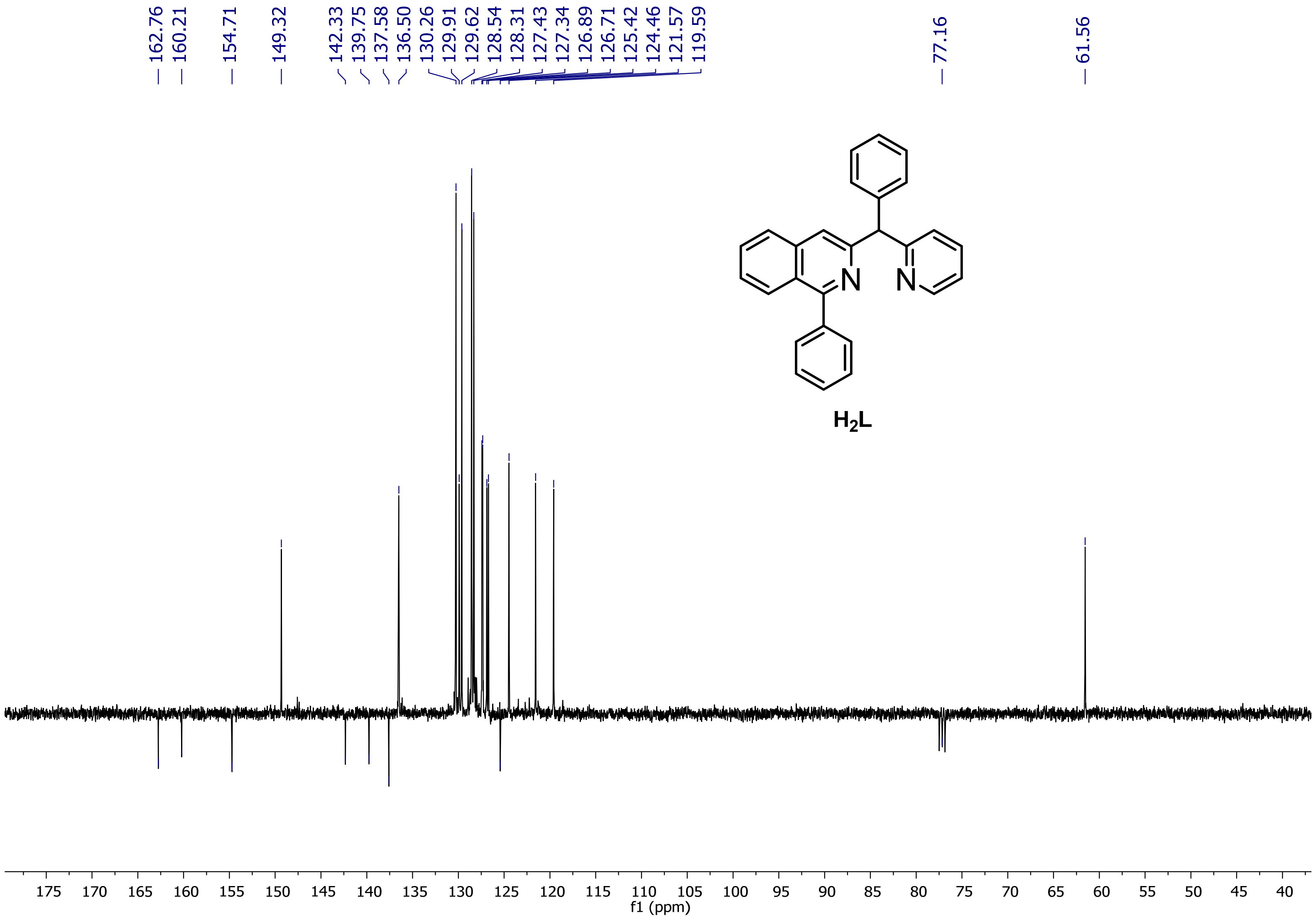

Figure S50. $^{13}C\{^{1}H\}$-APT NMR (100 MHz, $CDCl_3$, 298 K) spectrum of H_2L.

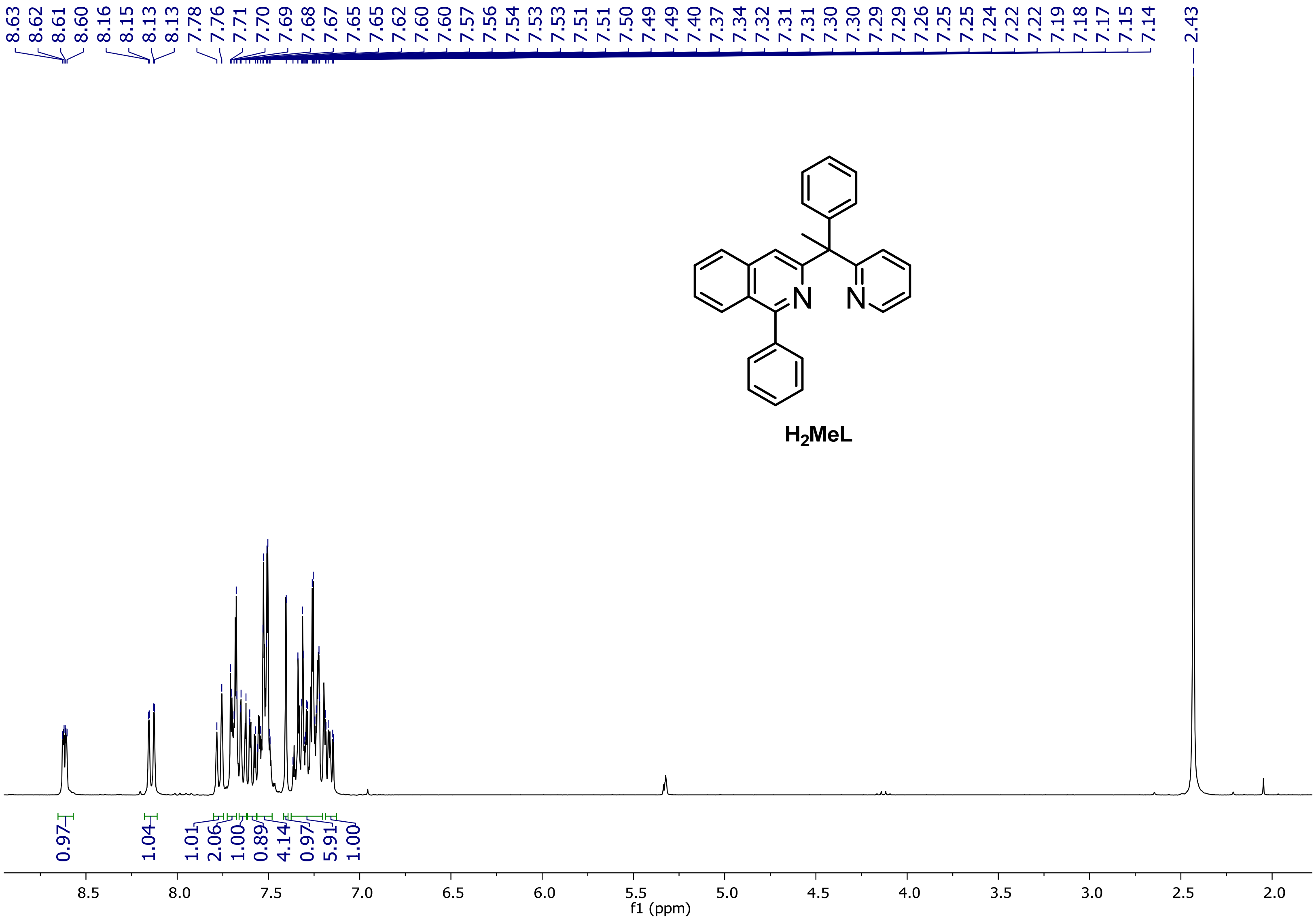

Figure S51. ^{1}H NMR (300 MHz, CD_2Cl_2, 298 K) spectrum of H_2MeL.

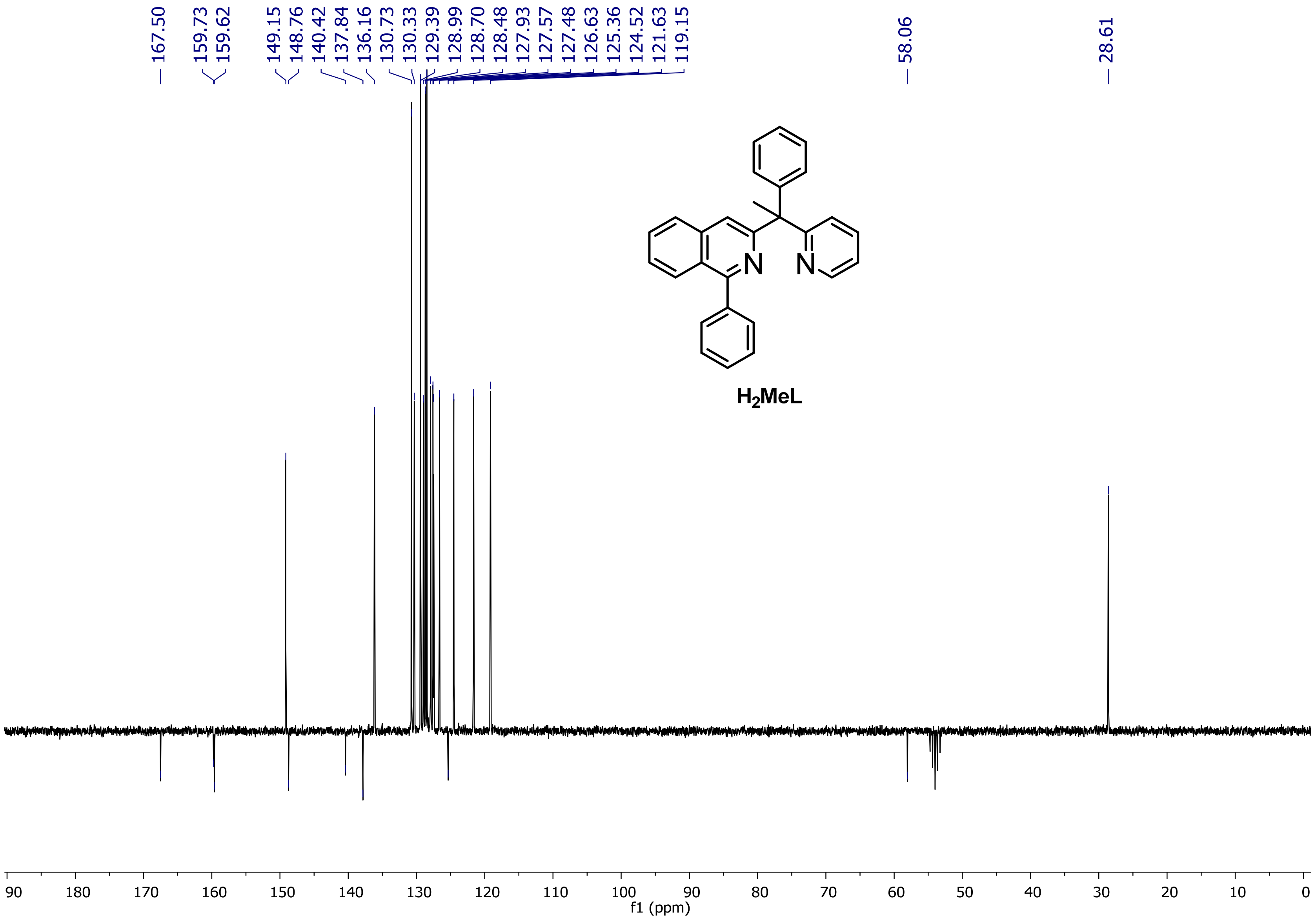

Figure S52. $^{13}C\{^{1}H\}$-APT NMR (75 MHz, CD_2Cl_2, 298 K) spectrum of H_2MeL.

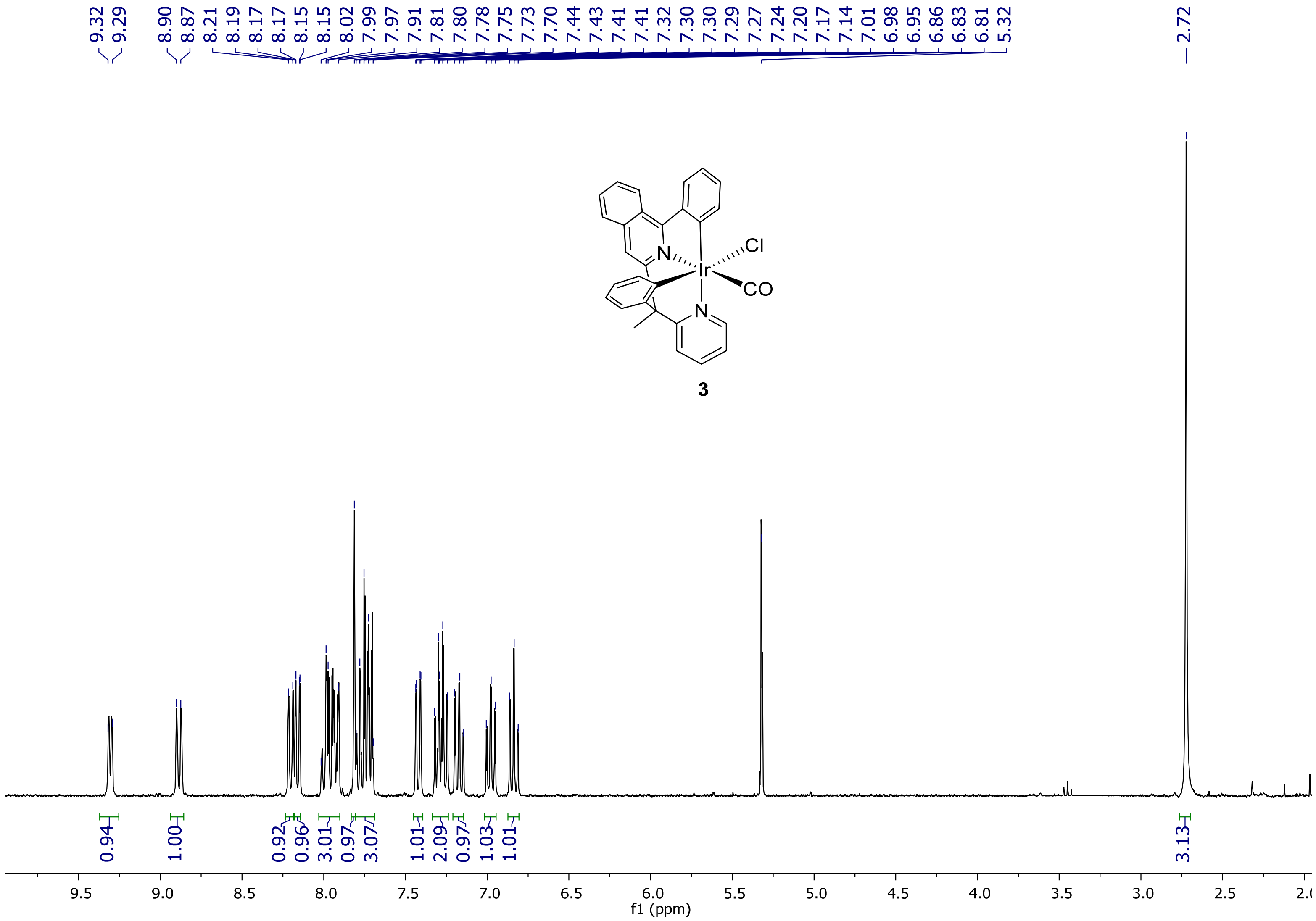

Figure S53. ^{1}H NMR (300 MHz, CD_2Cl_2, 298 K) spectrum of complex **3**.

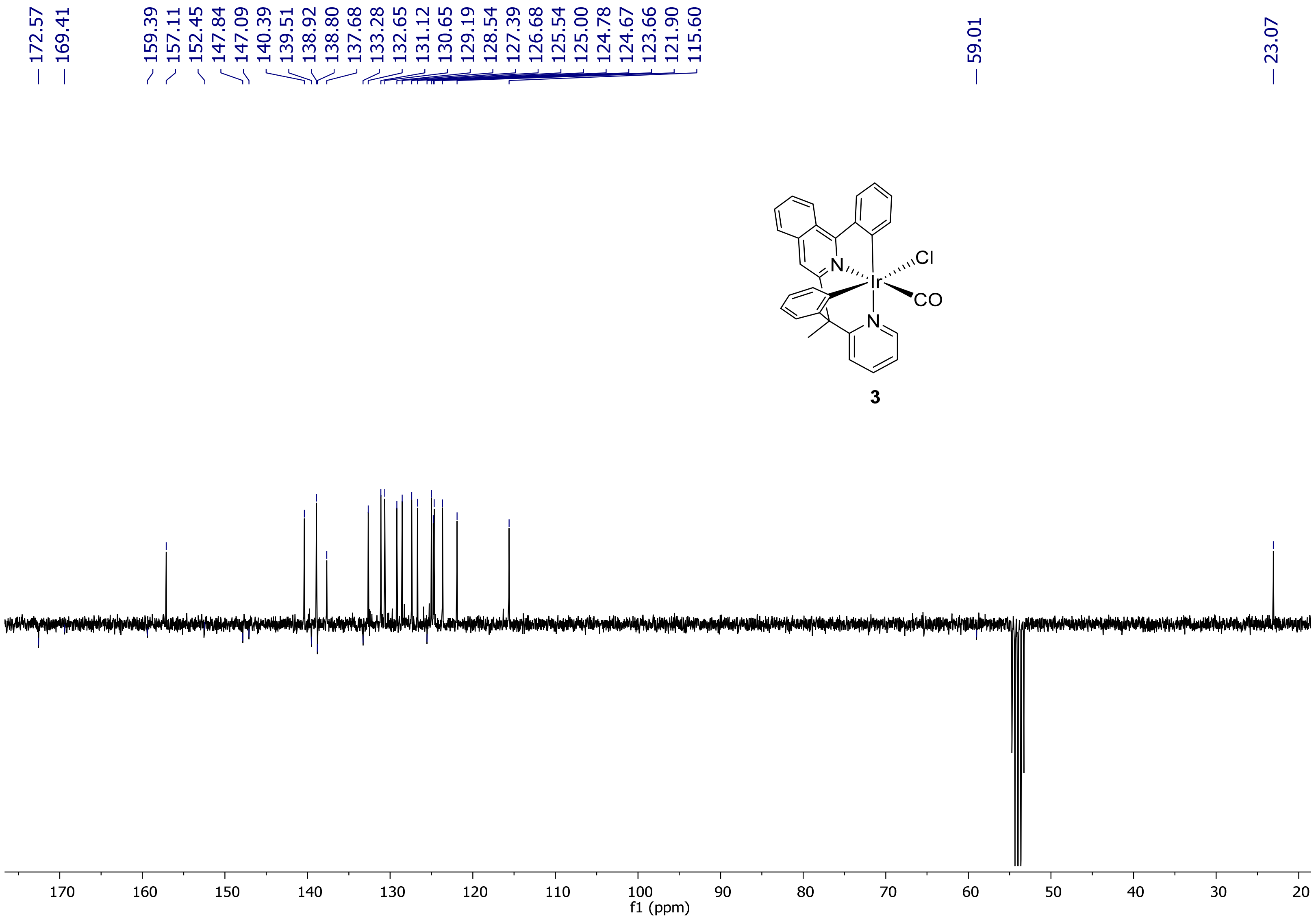

Figure S54 . $^{13}C\{^1H\}$-APT NMR (75 MHz, CD_2Cl_2, 298 K) spectrum of (**3**).

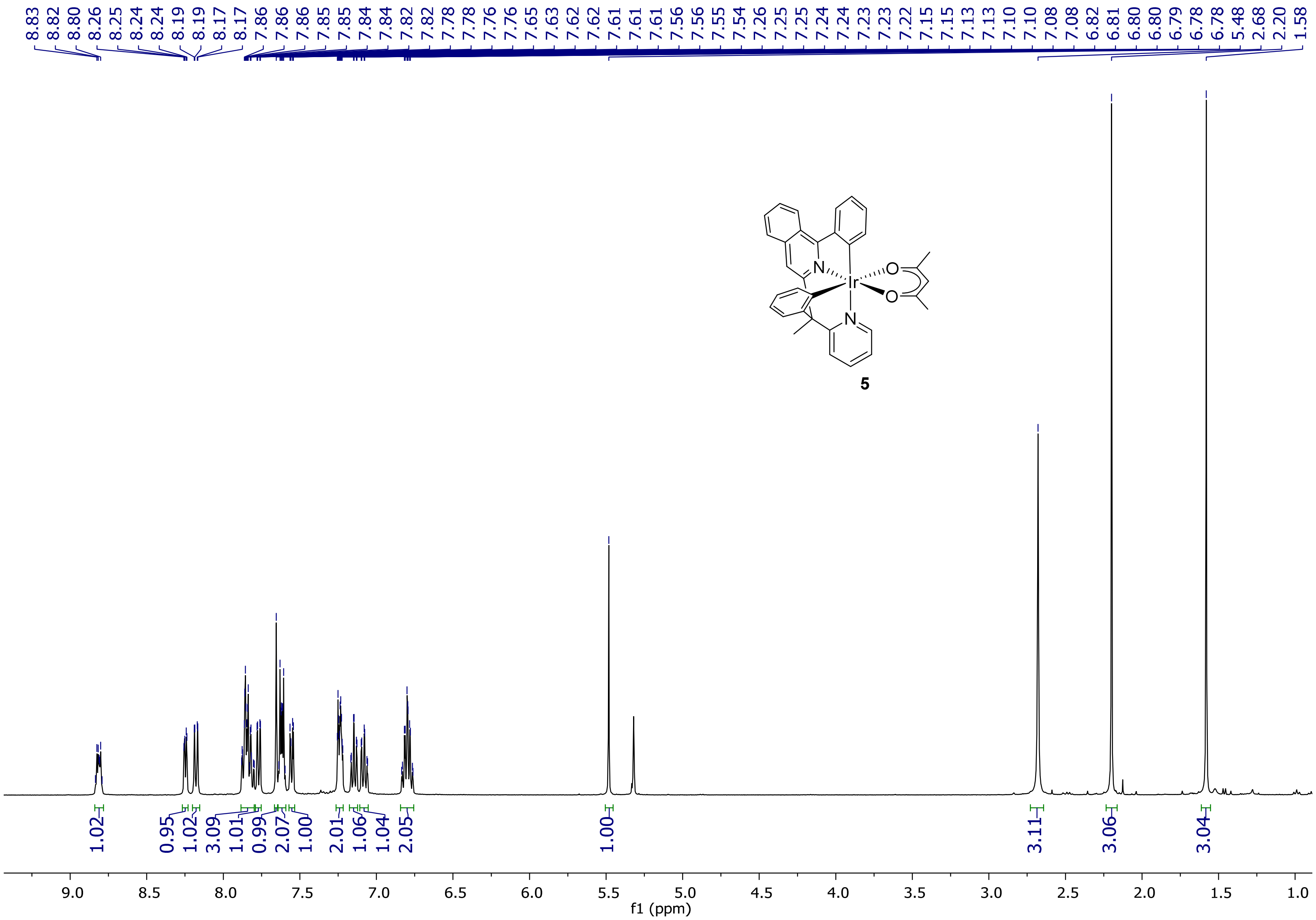

Figure S55. ^{1}H NMR (400 MHz, CD_2Cl_2, 298 K) spectrum of complex **5**.

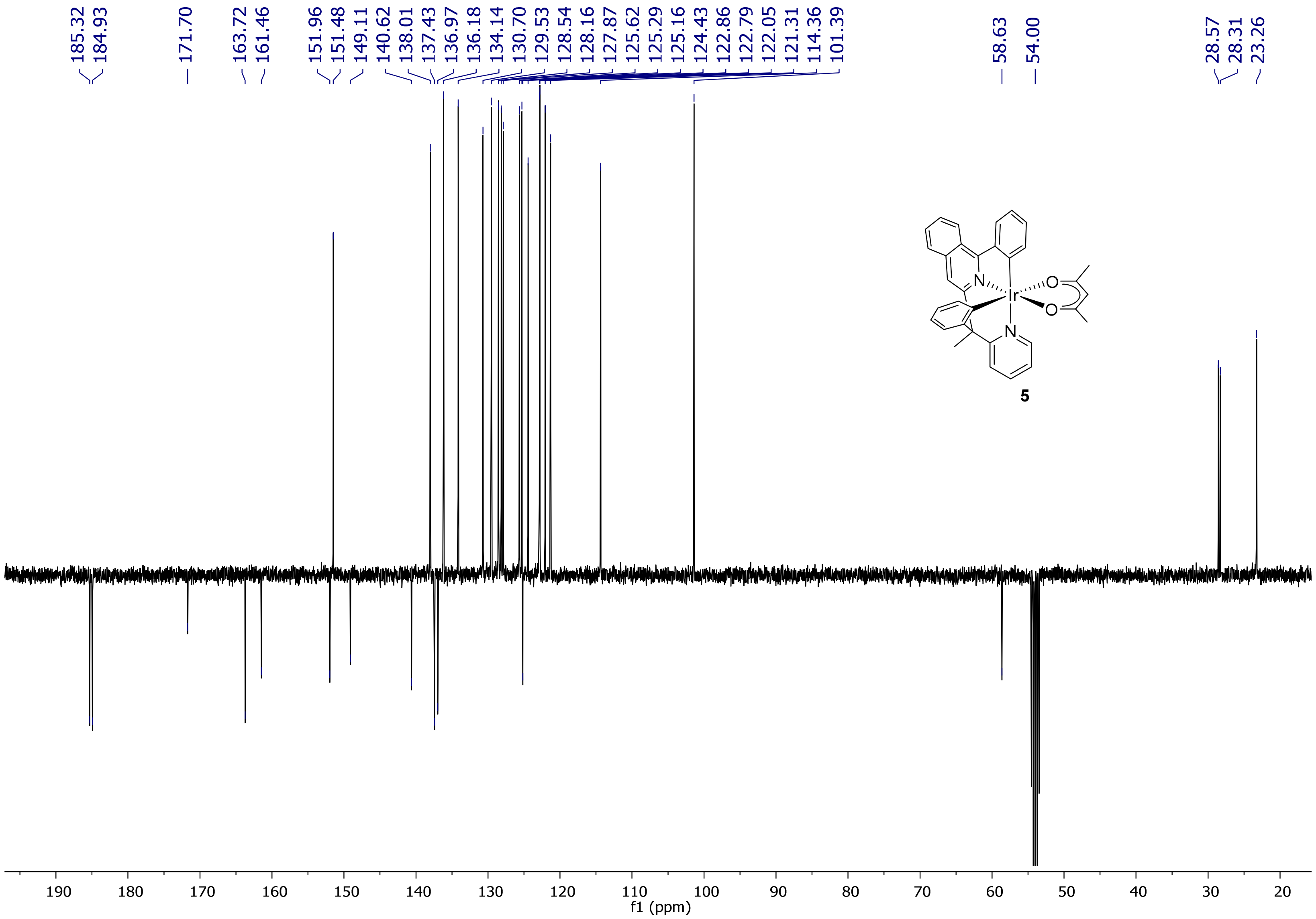

Figure S56. $^{13}C\{^{1}H\}$-APT NMR (100 MHz, CD_2Cl_2, 298 K) spectrum of complex **5**.

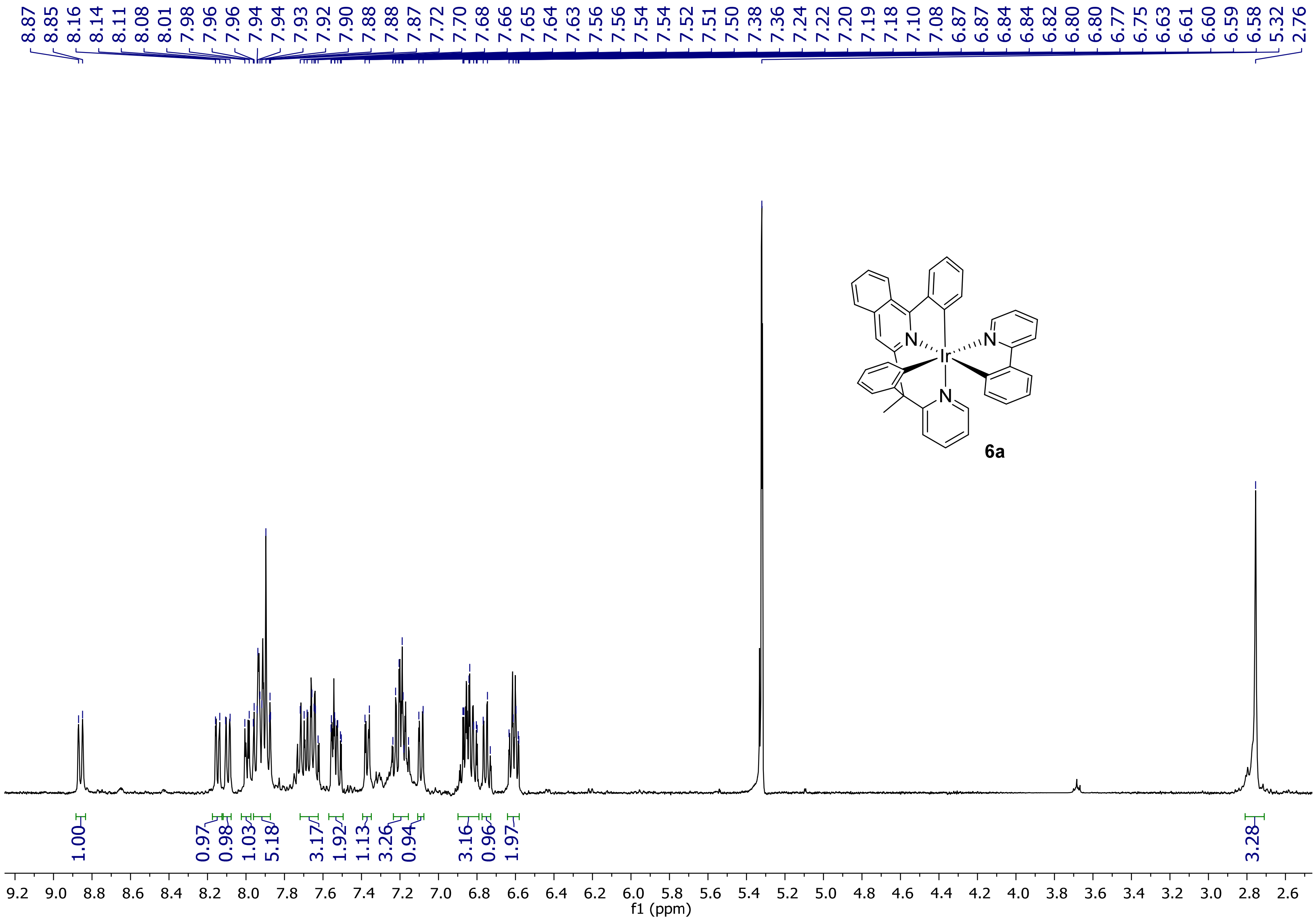

Figure S57. ^{1}H NMR (400 MHz, CD_2Cl_2, 298 K) spectrum of complex **6a**.

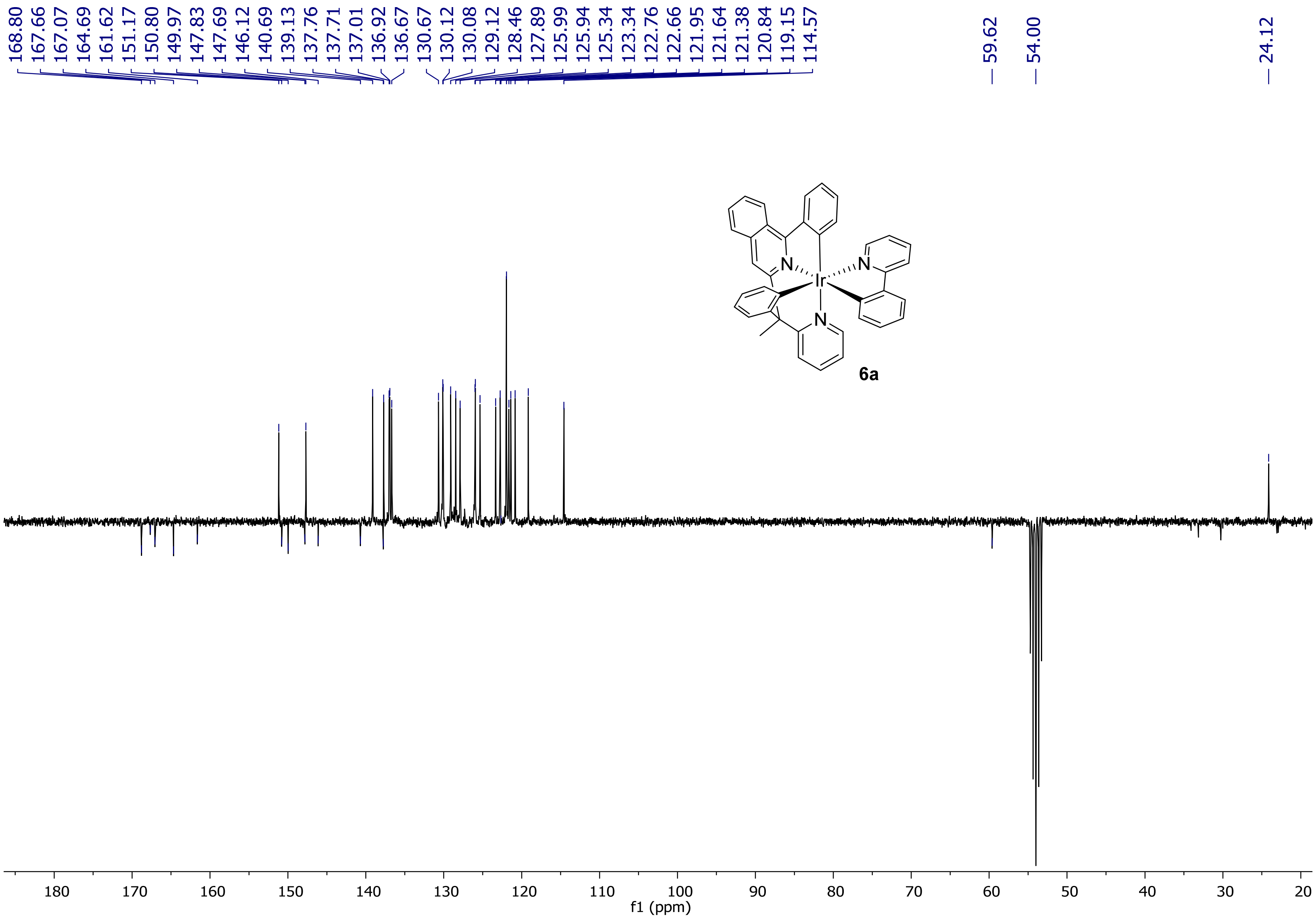

Figure S58. $^{13}C\{^{1}H\}$-APT NMR (75 MHz, CD_2Cl_2, 298 K) spectrum of complex **6a**.

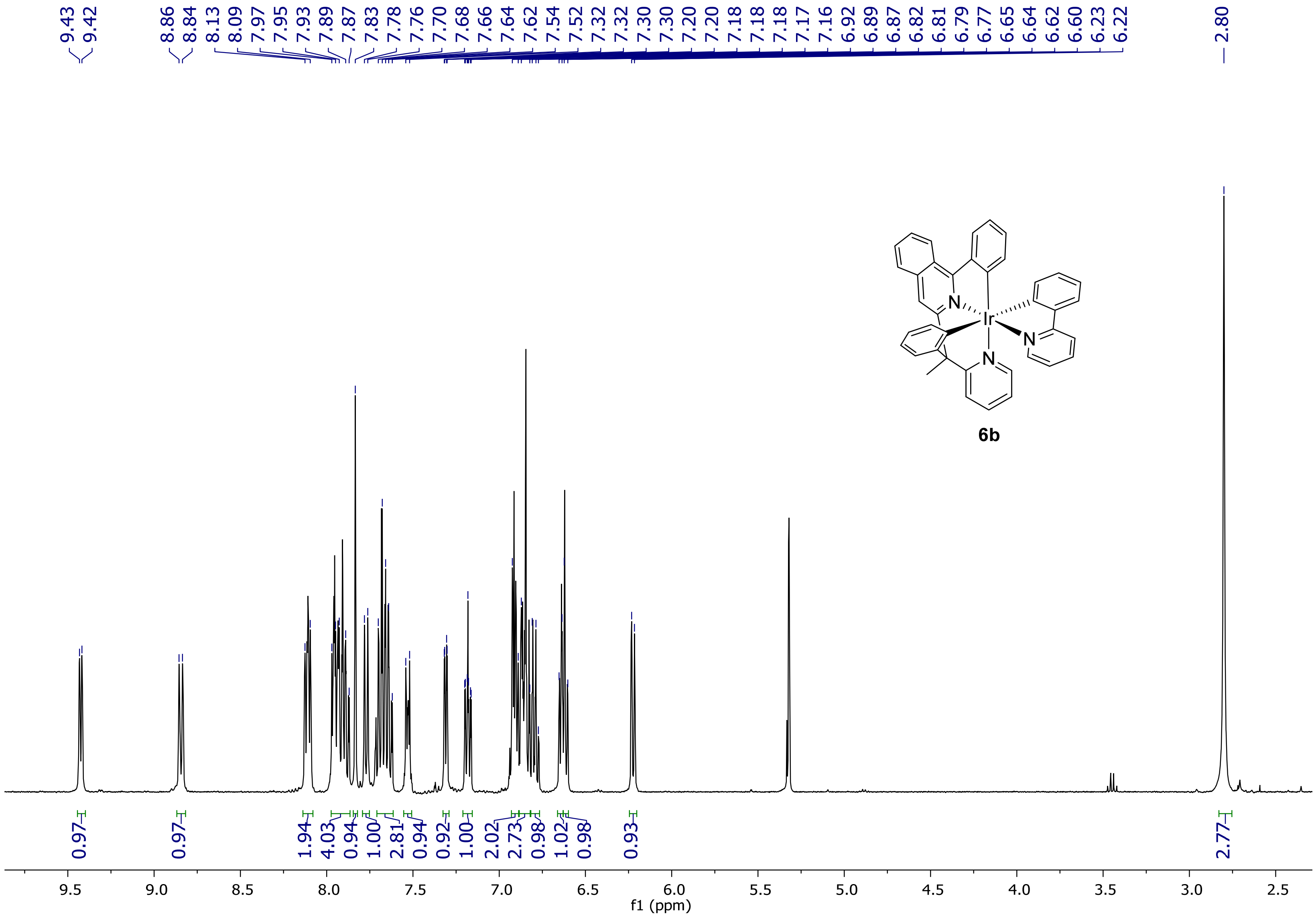

Figure S59. ^{1}H NMR (400 MHz, CD_2Cl_2, 298 K) spectrum of complex **6b**.

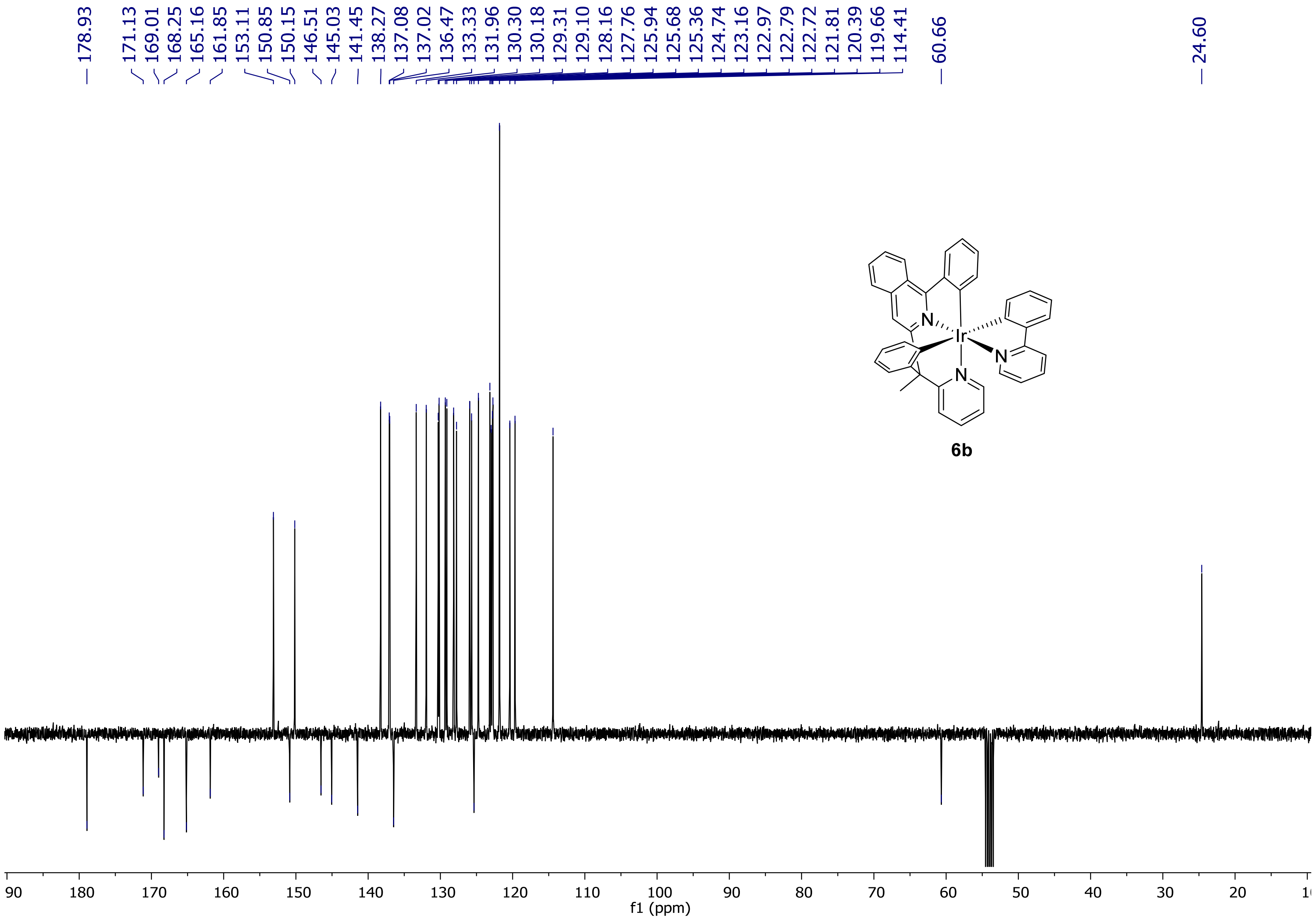

Figure S60 . $^{13}C\{^{1}H\}$-APT NMR (100 MHz, CD_2Cl_2, 298 K) spectrum of complex **6b**.

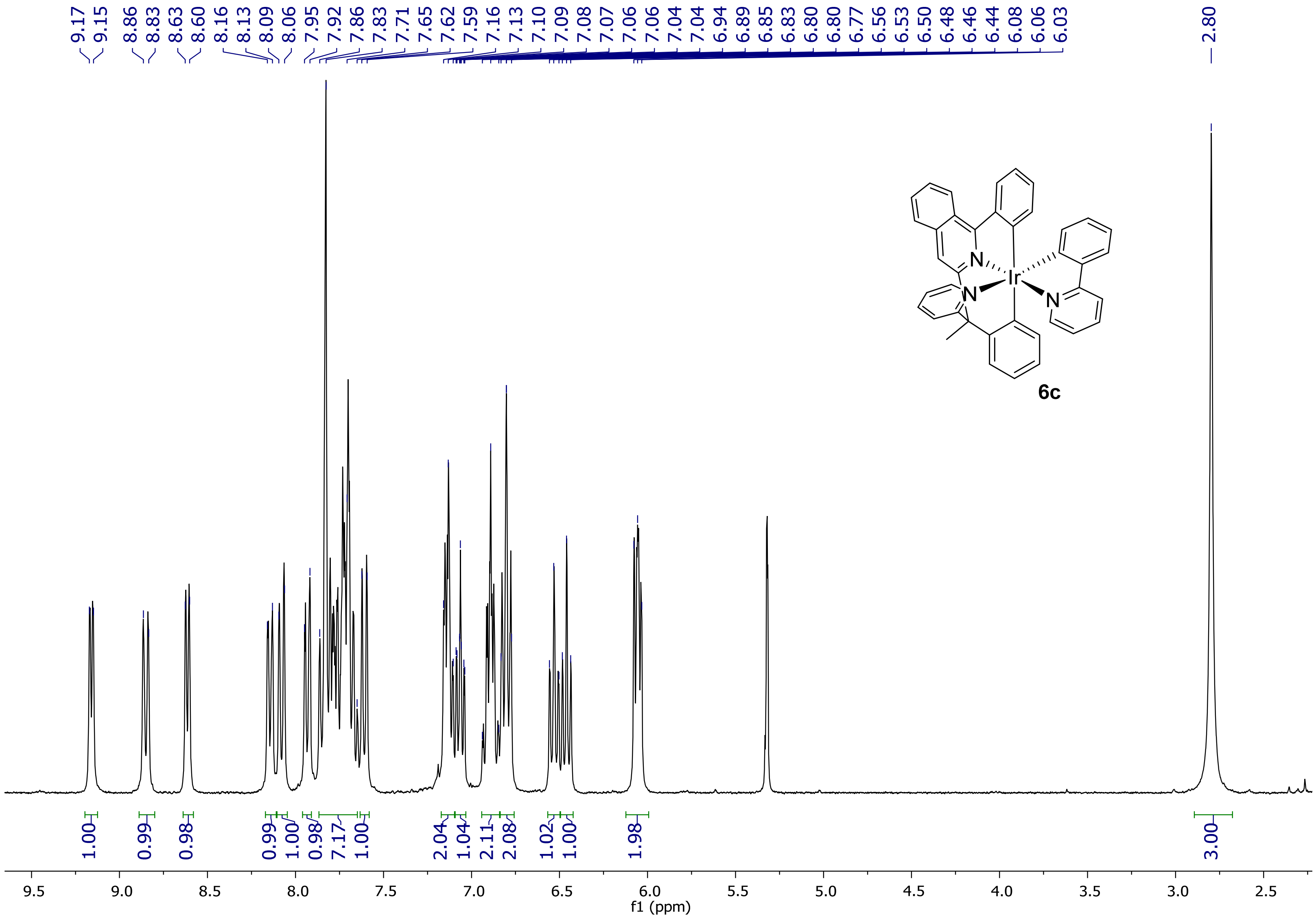

Figure S61. ^{1}H NMR (300 MHz, CD_2Cl_2, 298 K) spectrum of complex **6c**.

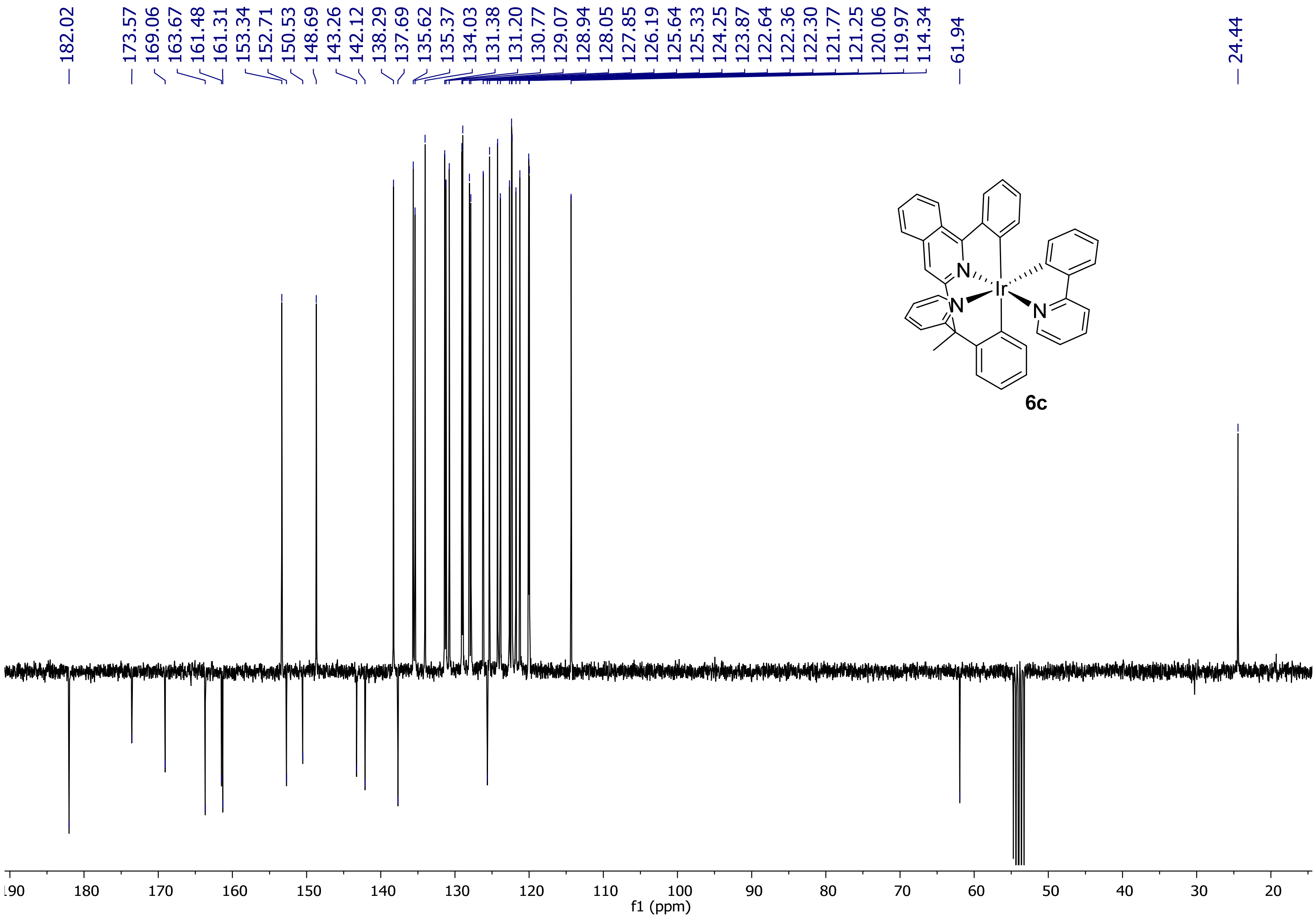

Figure S62 . $^{13}C\{^{1}H\}$-APT NMR (75 MHz, CD_2Cl_2, 298 K) spectrum of **6c**.

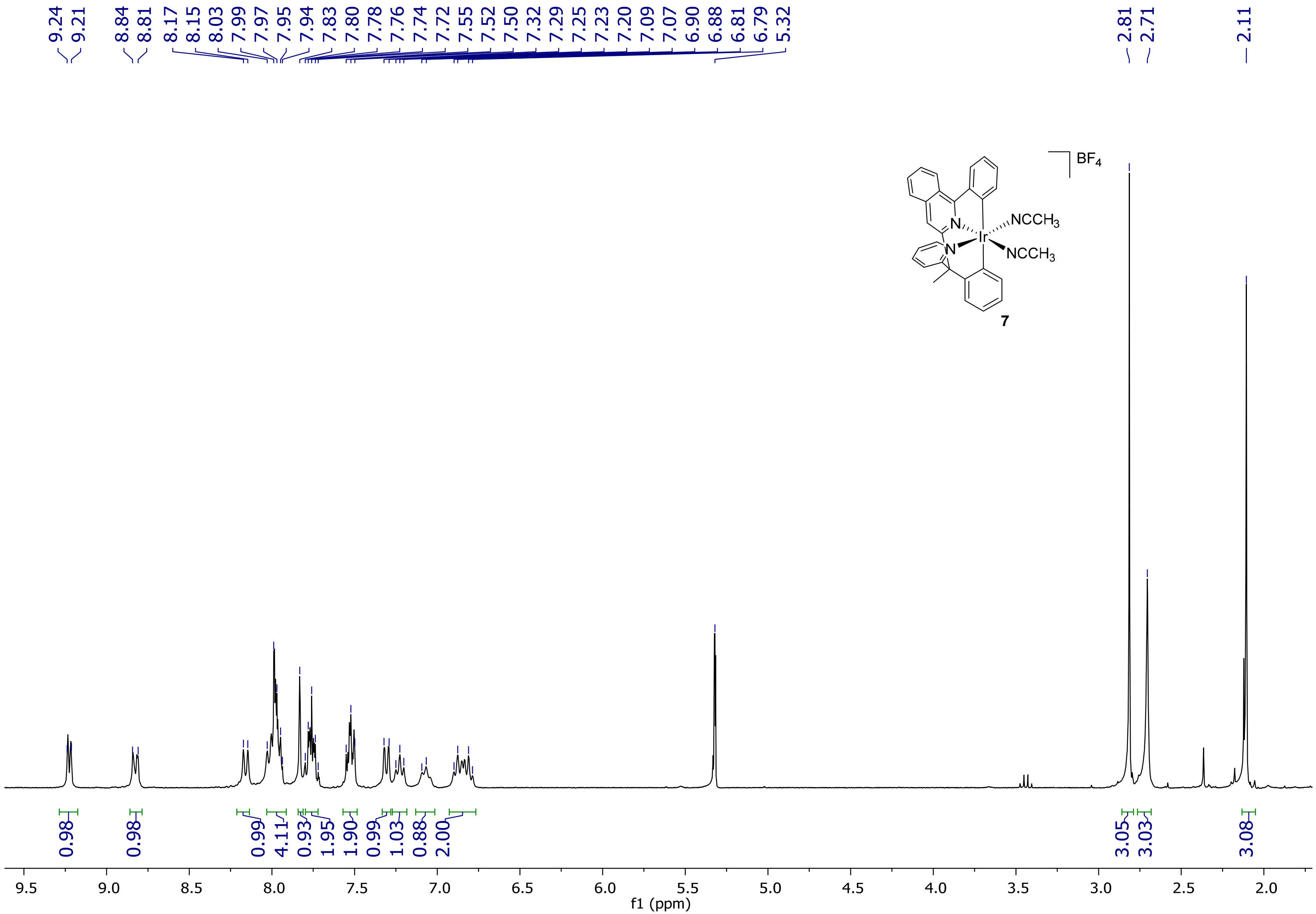

Figure S63. ^{1}H NMR (300 MHz, CD_2Cl_2, 298 K) spectrum of complex **7**.

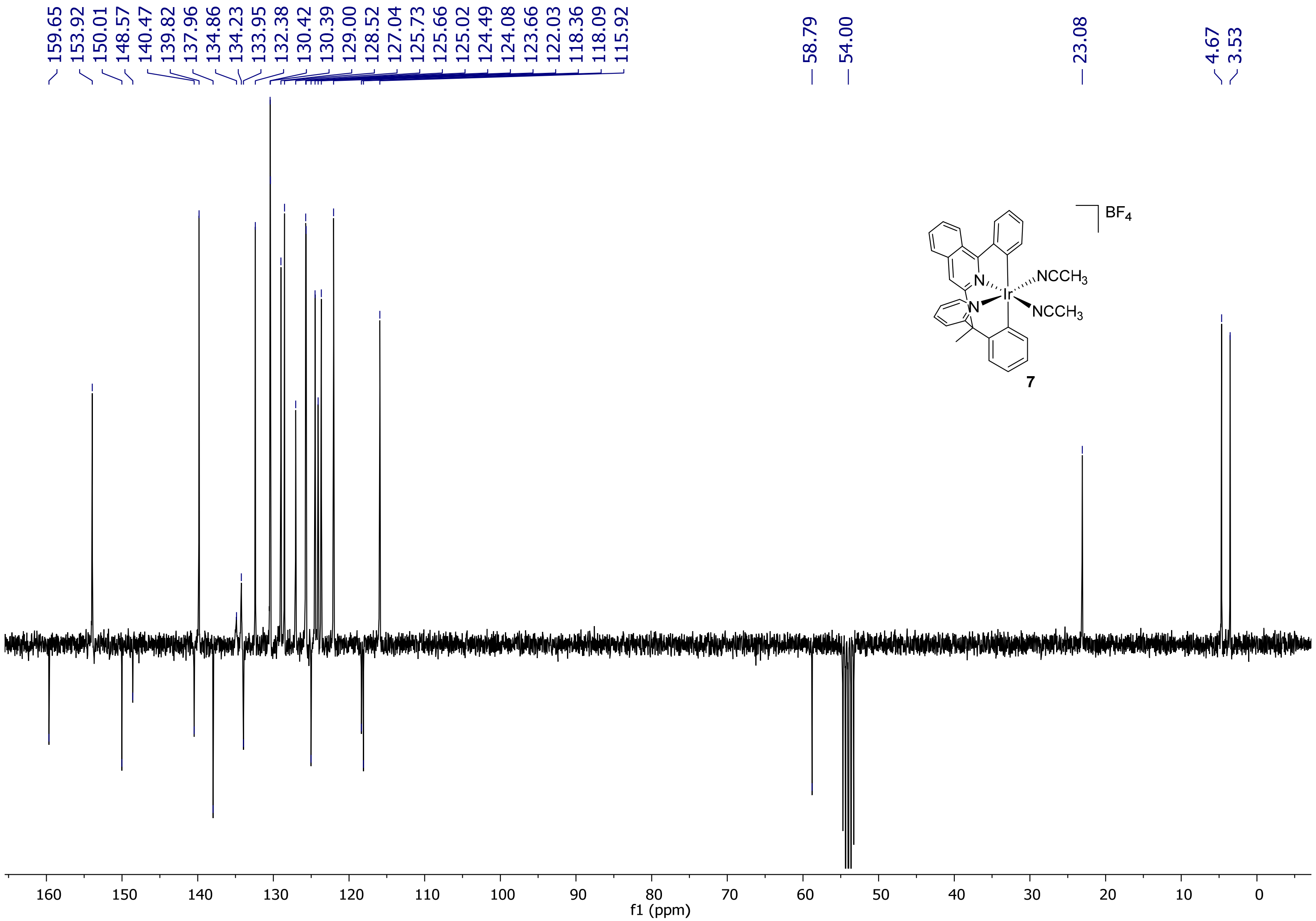

Figure S64 . $^{13}C\{^1H\}$-APT NMR (75 MHz, CD_2Cl_2, 298 K) spectrum of complex **7**.

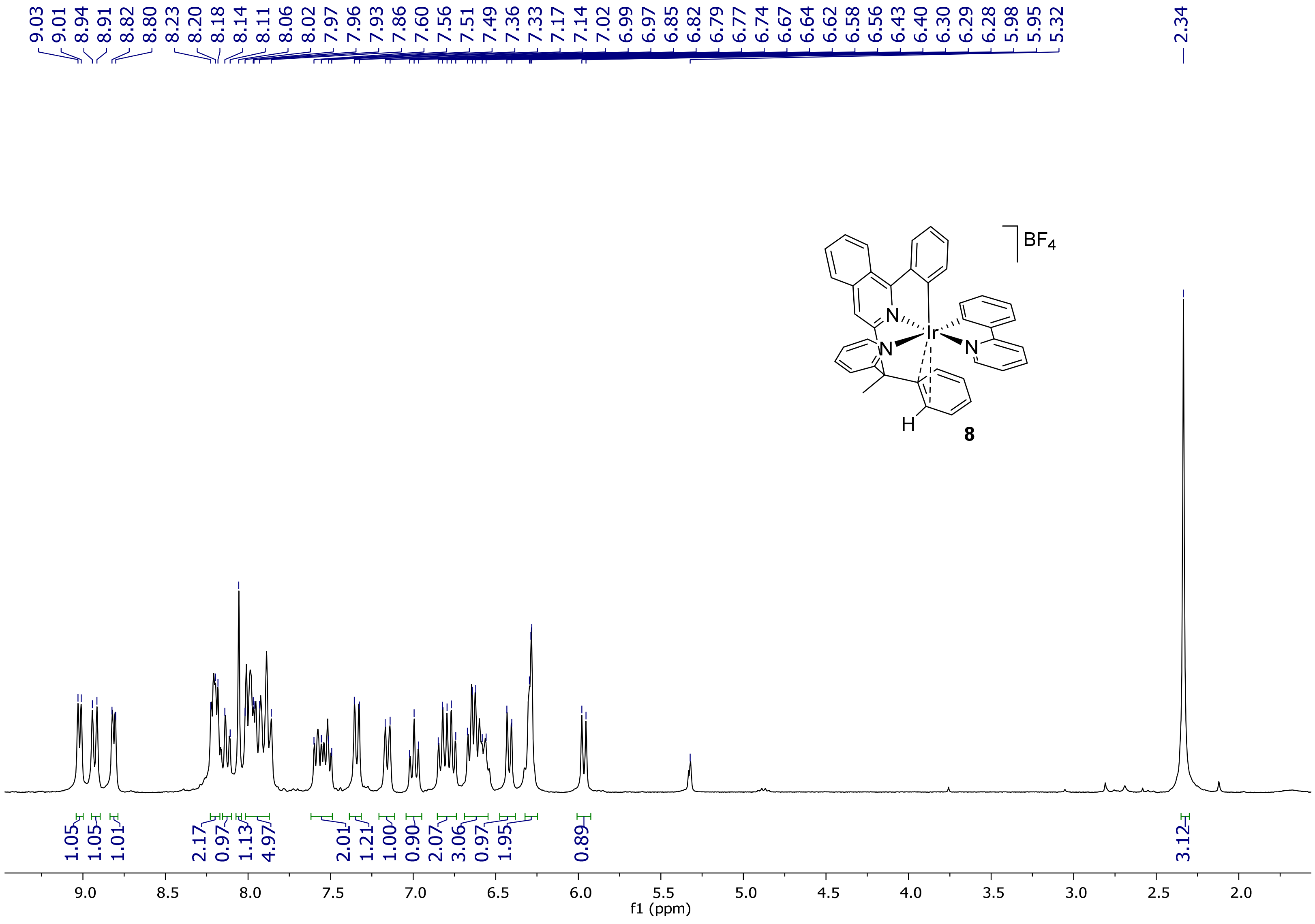

Figure S65. ^{1}H NMR (300 MHz, CD_2Cl_2, 298 K) spectrum of complex **8**.

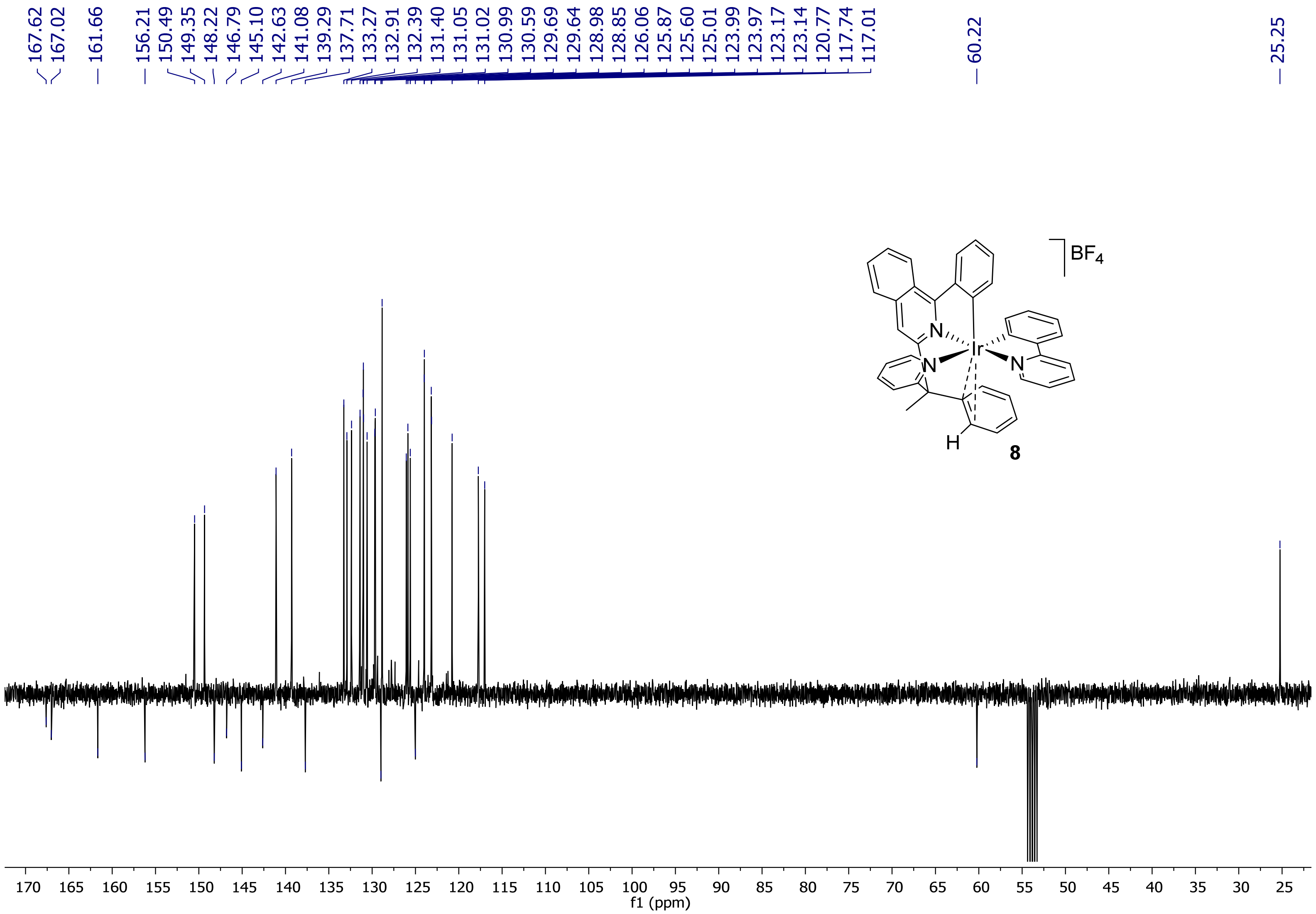

Figure S66. $^{13}C\{^{1}H\}$-APT NMR (100 MHz, CD_2Cl_2, 298 K) spectrum of complex **8**.

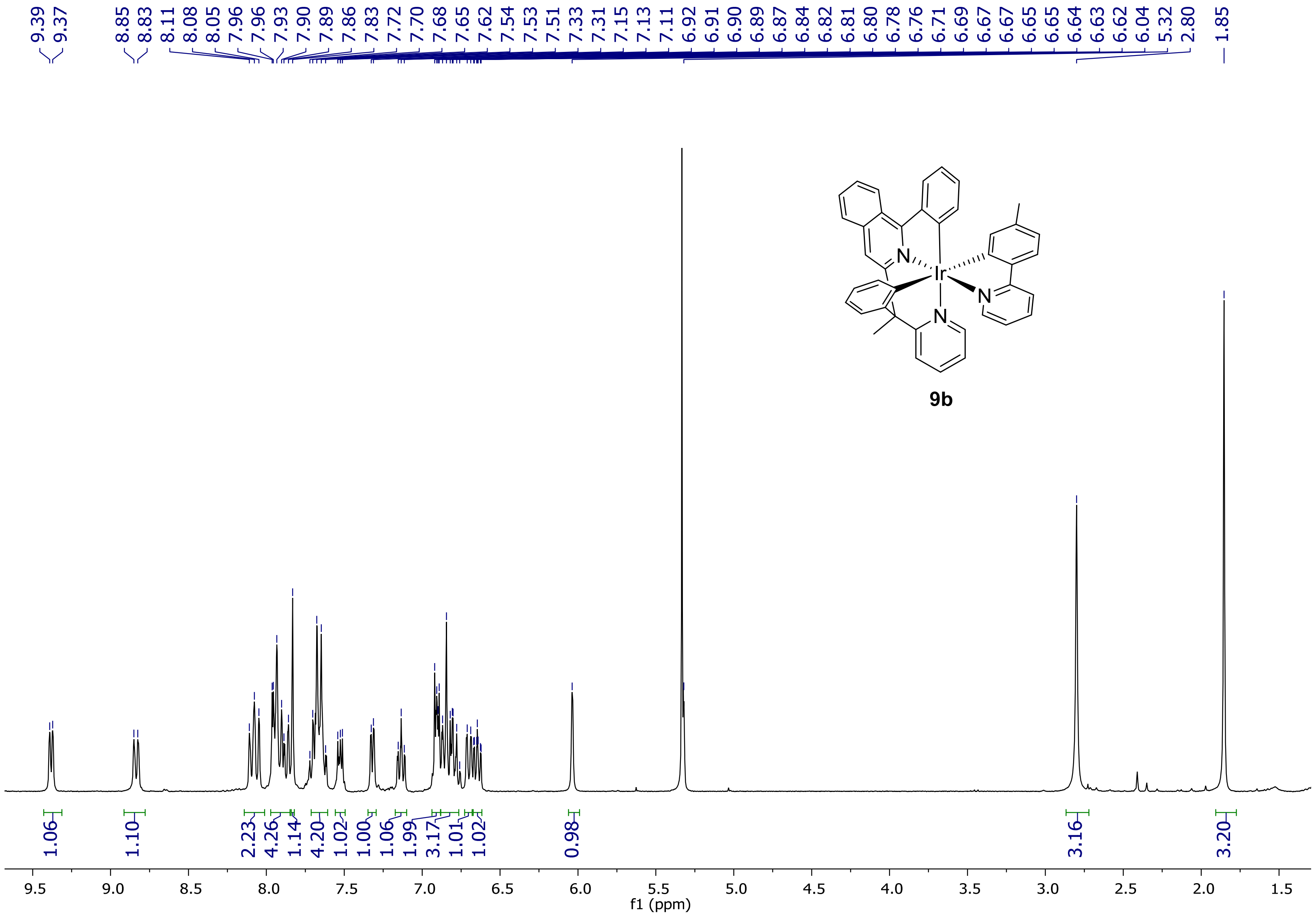

Figure S67. ^{1}H NMR (300 MHz, CD_2Cl_2, 298 K) spectrum of complex **9b**.

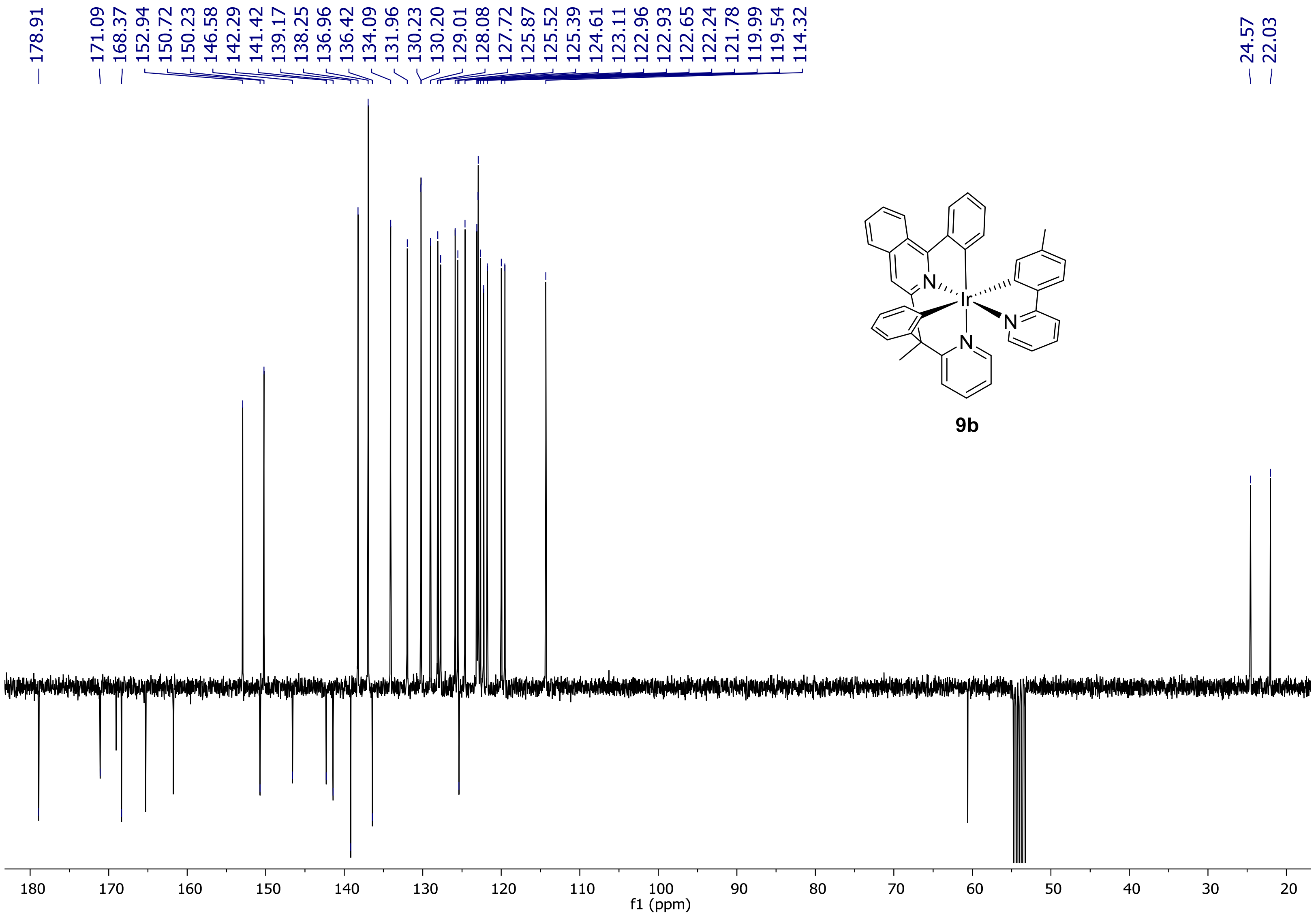

Figure S68. $^{13}C\{^{1}H\}$-APT NMR (75 MHz, CD_2Cl_2, 298 K) spectrum of complex **9b**.

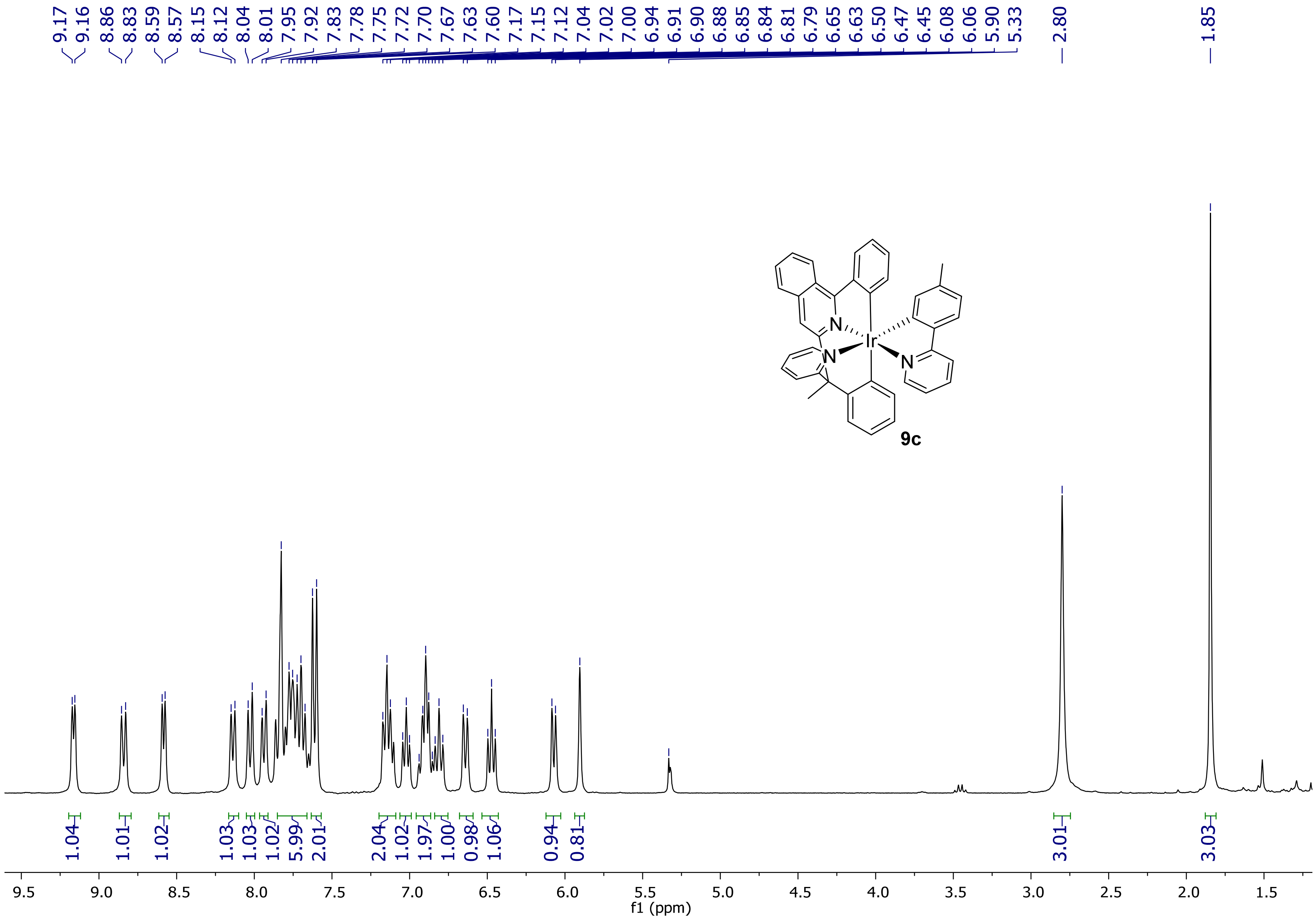

Figure S69. ^{1}H NMR (300 MHz, CD_2Cl_2, 298 K) spectrum of complex **9c**.

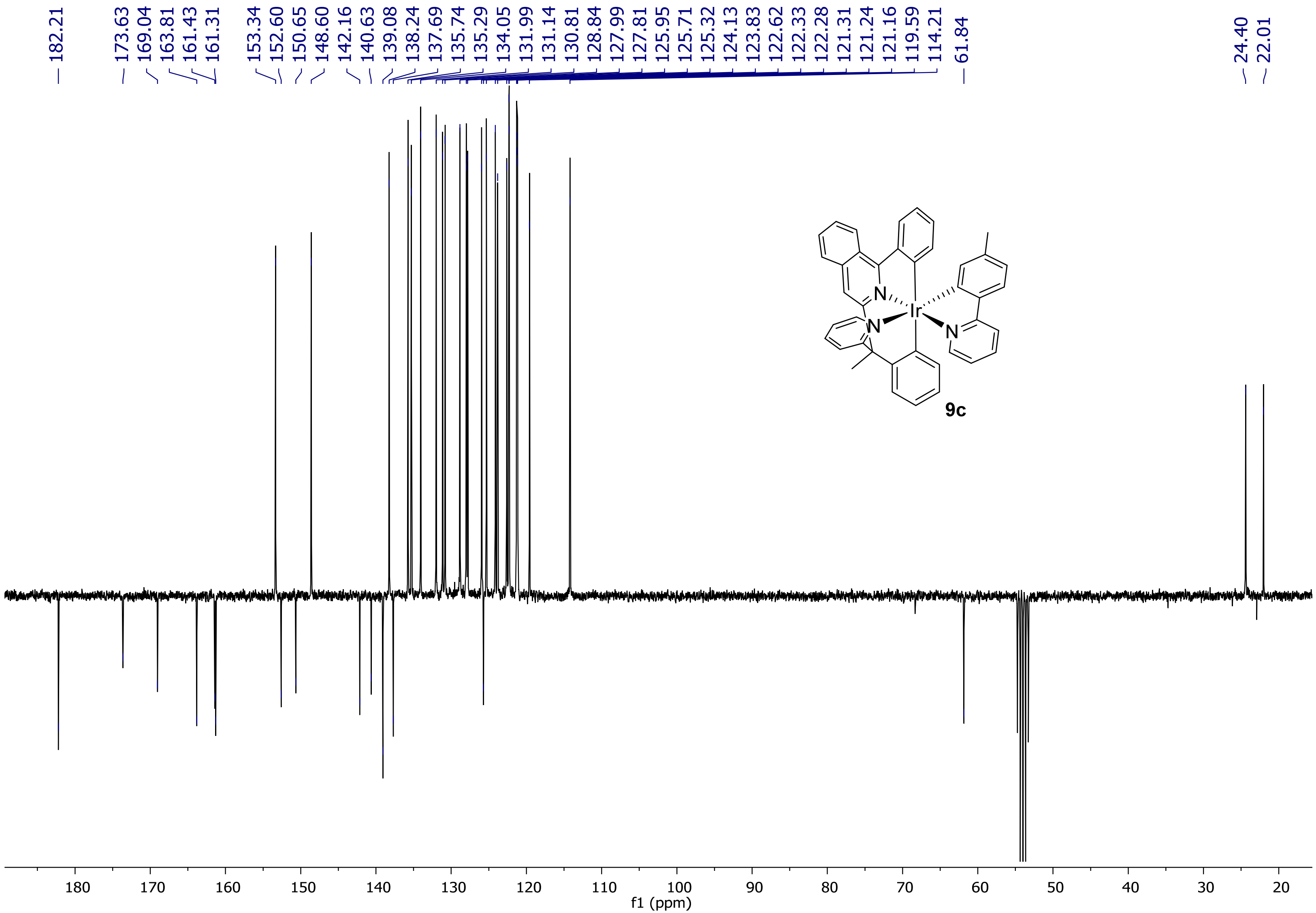

Figure S70. $^{13}C\{^{1}H\}$-APT NMR (75 MHz, CD_2Cl_2, 298 K) spectrum of complex **9c**.

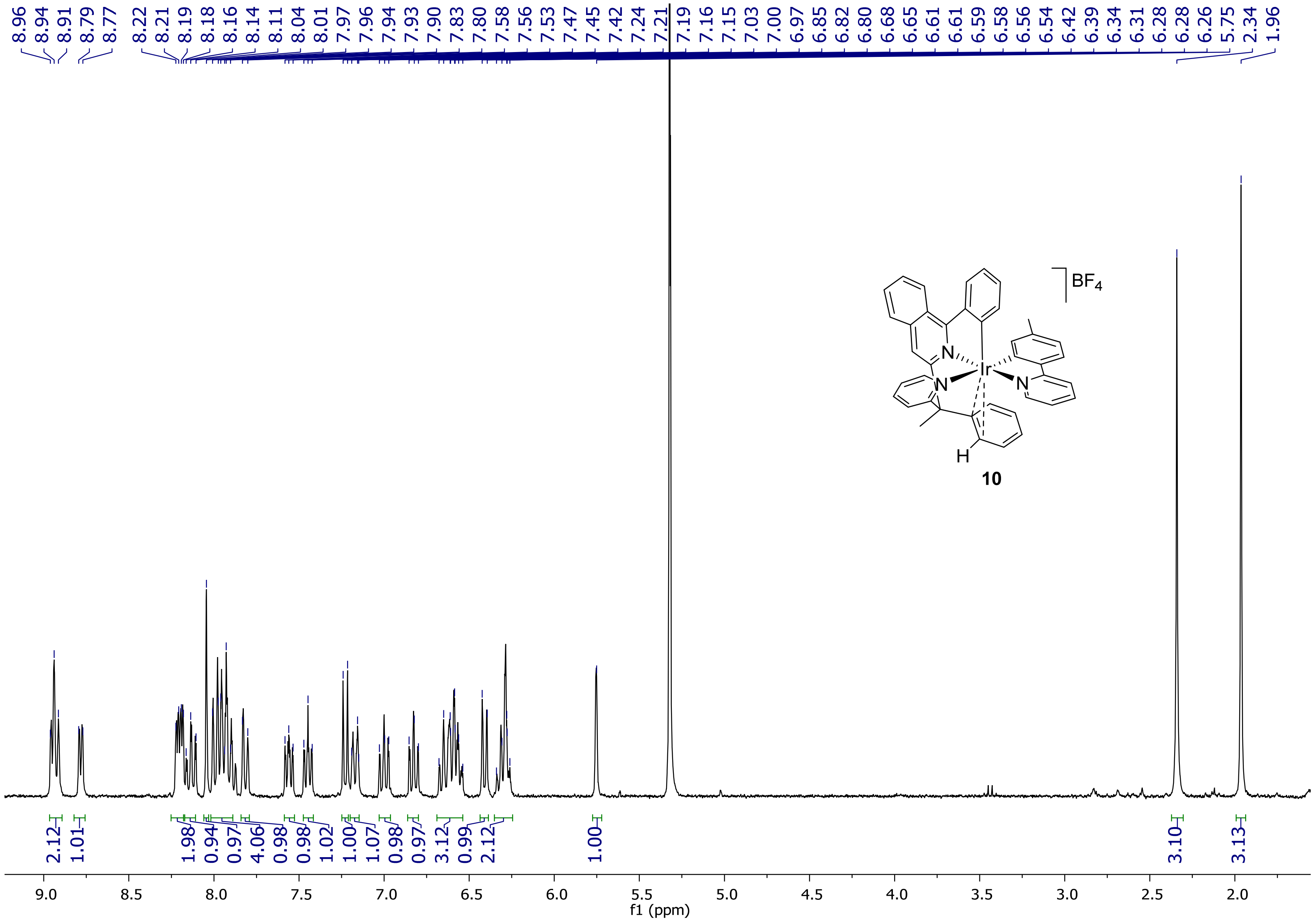

Figure S71. ^{1}H NMR (300 MHz, CD_2Cl_2, 298 K) spectrum of complex **10**.

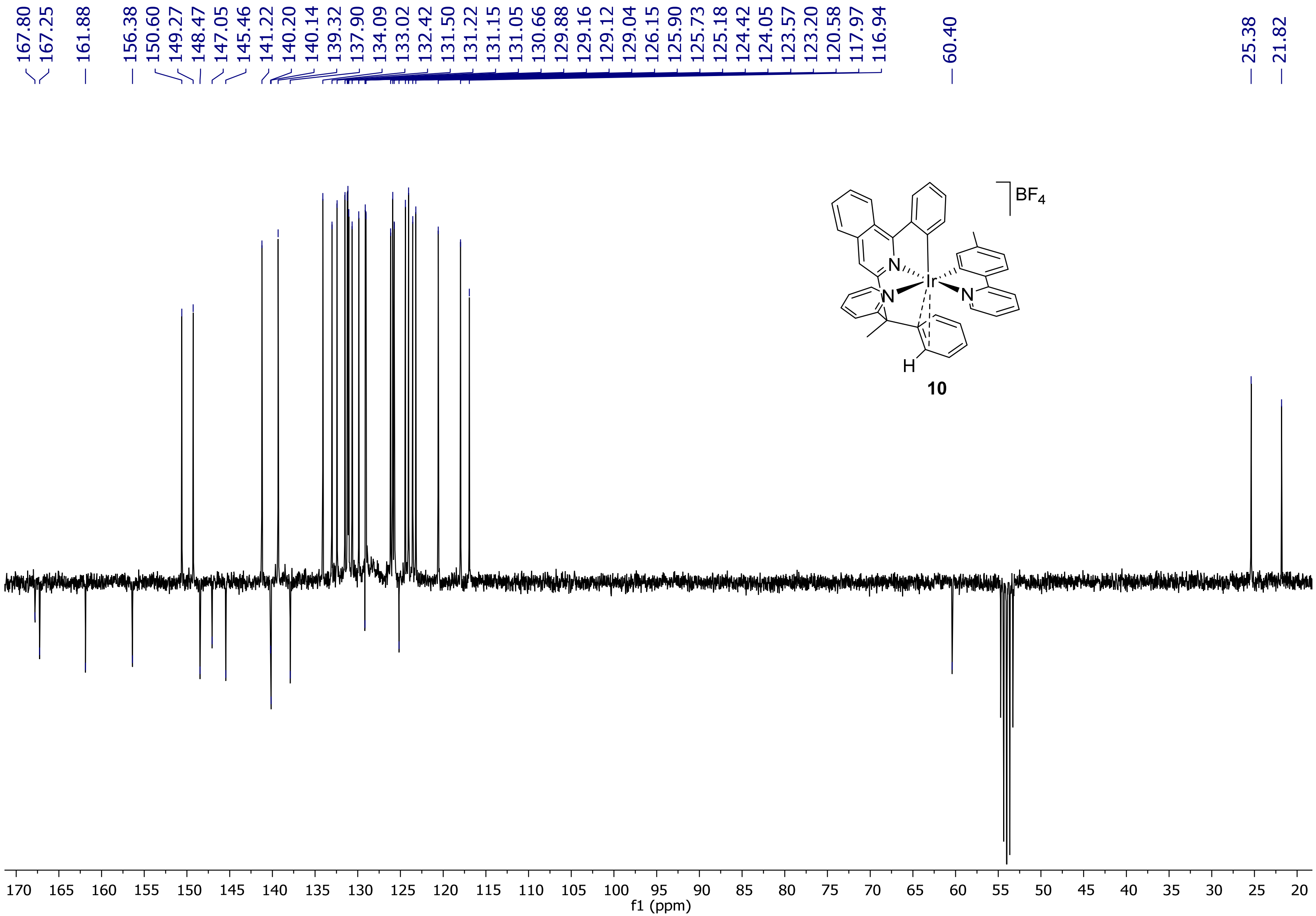

Figure S72 . $^{13}C\{^{1}H\}$-APT NMR (75 MHz, CD_2Cl_2, 298 K) spectrum of complex **10**.

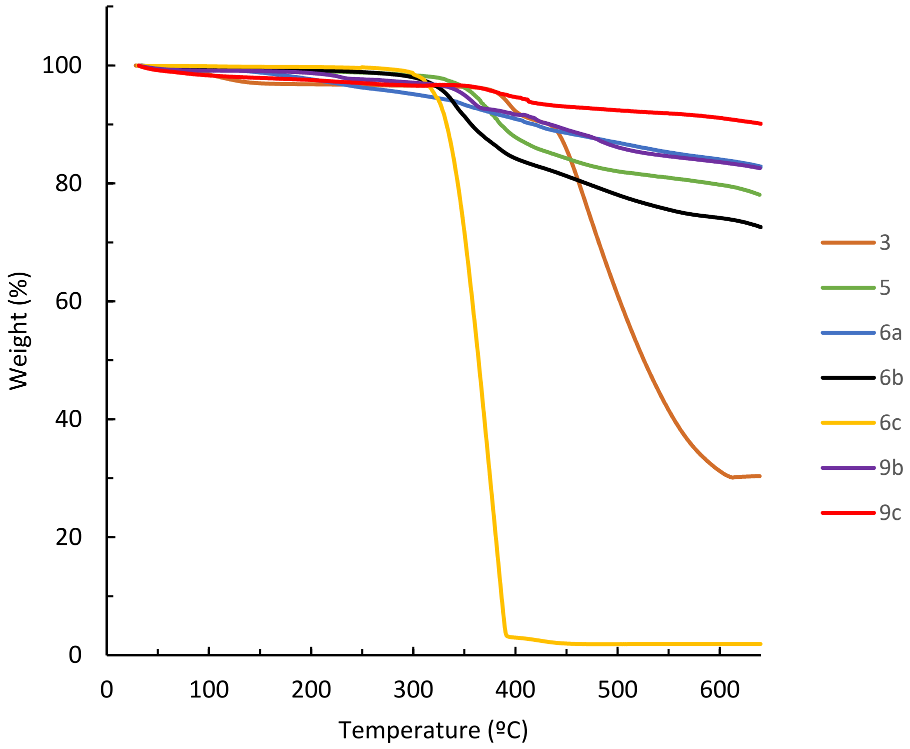

Figure S73. TGA curves of complexes **3**, **5**, **6a-c** and **9b**,**c**. The solids were heated under nitrogen at 10 ºC/min under nitrogen.

References

(1) Blessing, R. H. An Empirical Correction for Absorption Anisotropy. *Acta Crystallogr.* **1995**, *A51*, 33. SADABS: Area-detector absorption correction; Bruker- AXS, Madison, WI, 1996.

(2) SHELXL-2016/6. Sheldrick, G. M. A short history of SHELX. *Acta Cryst.* **2008**, *A64*, 112-122.

(3) (a) Lee, C.; Yang, W.; Parr, R. G. Development of the Colle-Salvetti correlation-energy formula into a functional of the electron density. *Phys. Rev. B* **1988**, *37*, 785–789. (b) Becke, A. D. Density-functional exchange-energy approximation with correct asymptotic behavior. *J. Chem. Phys.* **1993**, *98*, 5648–5652. (c) Stephens, P. J.; Devlin, F. J.; Chabalowski, C. F.; Frisch, M. J. *Ab Initio* Calculation of Vibrational Absorption and Circular Dichroism Spectra Using Density Functional Force Fields. *J. Phys. Chem.* **1994**, *98*, 11623–11627.

(4) Grimme, S.; Antony, J.; Ehrlich, S.; Krieg, H. A consistent and accurate *ab initio* parametrization of density functional dispersion correction (DFT-D) for the 94 elements H-Pu. *J. Chem. Phys.* **2010**, *132*, 154104.

(5) Gaussian 09, Revision D.01, Frisch, M. J.; Trucks, G. W.; Schlegel H. B.; Scuseria, G. E.; Robb, M. A.; Cheeseman, J. R.; Scalmani, G.; Barone, V.; Mennucci, B.; Petersson, G. A.; Nakatsuji, H.; Caricato, M.; Li, X.; Hratchian, H. P.; Izmaylov, A. F.; Bloino, J.; Zheng, G.; Sonnenberg, J. L.; Hada, M.; Ehara, M.; Toyota, K.; Fukuda, R.; Hasegawa, J.; Ishida, M.; Nakajima, T.; Honda, Y.; Kitao, O.; Nakai, H.; Vreven, T.; Montgomery, J. A.; Peralta, Jr., J. E.; Ogliaro, F.; Bearpark, M.; Heyd, J. J.; Brothers, E.; Kudin, K. N.; Staroverov, V. N.; Keith, T.; Kobayashi, R.; Normand, J.; Raghavachari, K.; Rendell, A.; Burant, J. C.; Iyengar, S. S.; Tomasi, J.; Cossi, M.; Rega, N.; Millam, J. M.; Klene, M.; Knox, J. E.; Cross, J. B.; Bakken, V.; Adamo, C.; Jaramillo, J.; Gomperts, R.; Stratmann, R. E.; Yazyev, O.; Austin, A. J.; Cammi, R.; Pomelli, C.; Ochterski, J. W.; Martin, R. L.; Morokuma, K.; Zakrzewski, V. G.; Voth, G. A.; Salvador, P.; Dannenberg, J. J.; Dapprich, S.; Daniels, A. D.; Farkas, O.; Foresman, J. B.; Ortiz, J. V.; Cioslowski, J.; Fox, D. J. Gaussian, Inc., Wallingford CT, 2013.

(6) Andrea, D.; Häußermann, U. M.; Dolg, M.; Stoll, H.; Preuss, H. Energy-adjusted *ab initio* pseudopotentials for the second and third row transition elements. *Theor. Chim. Acta* **1990**, *77*, 123–141.

(7) Ehlers, A. W.; Bohme, M.; Dapprich, S.; Gobbi, A.; Hollwarth, A.; Jonas, V.; Kohler, K. F.; Stegmann, R.; Veldkamp, A.; Frenking, G. A set of f-polarization functions for pseudo-potential basis sets of the transition metals SC-Cu, Y-Ag and La-Au. *Chem. Phys. Lett.* **1993**, *208*, 111–114.

(8) (a) Hehre, W. J.; Ditchfield, R.; Pople, J. A. Self-Consistent Molecular Orbital Methods. XII. Further Extensions of Gaussian-Type Basis Sets for Use in Molecular Orbital Studies of Organic Molecules. *J. Chem. Phys.* **1972**, *56*, 2257–2261. (b) Francl, M. M.; Pietro, W. J.; Hehre, W. J.; Binkley, J. S.; Gordon, M. S.; DeFrees, D. J.; Pople, J. A. Self-consistent molecular orbital methods. XXIII. A polarization-type basis set for second-row elements. *J. Chem. Phys.* **1982**, *77*, 3654–3665.

(9) Marenich, A. V.; Cramer, C. J.; Truhlar, D. G. Universal Solvation Model Based on Solute Electron Density and on a Continuum Model of the Solvent Defined by the Bulk Dielectric Constant and Atomic Surface Tensions. *J. Phys. Chem. B* **2009**, *113*, 6378–6396.

(10) O'Boyle, N. M.; Tenderholt, A. L.; Langner, K. M. cclib: A Library for Package-Independent Computational Chemistry Algorithms.*J. Comput. Chem.* **2008**, *29*, 839−845.
